# Supplementary material for: Does knowledge of liver fibrosis affect high-risk drinking behaviour (KLIFAD)? protocol for a feasibility randomised controlled trial
Source: BMJ Open. 2021 Nov 3;11(11):e054954. doi: 10.1136/bmjopen-2021-054954 (PMC8572412; doi:10.1136/bmjopen-2021-054954)
Supplement: Supplementary data [file bmjopen-2021-054954supp001.pdf]

SP-Definitions

The following definitions are relevant to the KLIFAD trial:

Recovery Definition
For the KLIFAD trial we adopted the following definition of "Recovery"

"A period of sustained abstinence from alcohol creating a deeply personal, unique process of change, a way of living a satisfying, hopeful and contributing life even with limitations caused by illness. A process involving the development of new meaning or purpose in one's life which maximises health and wellbeing and participation in the rights, roles and responsibilities of society"[1-4].

Recovery story
A story told by a person about their journey of recovery.

In KLIFAD we are using recovery stories which are primarily first-person lived experience accounts, which include elements of both adversity/struggle and of strength/success/survival related to AUD, and which refer to events or actions over a period. Some stories will include brief fragments presenting clinical perspectives on a case, provided by a clinician who worked with the narrator[5].

Story narrator
The person telling their own recovery story.

Story recipient
The person viewing, reading or listening to someone else's recovery story.

KLIFAD Library
A collection of recovery stories intended for use in the KLIFAD feasibility trial.

Alcohol misuse
The National Institute on Alcohol Abuse and Alcoholism (NIAAA) define alcohol misuse as "alcohol consumption that puts individuals at increased risk for adverse health and social consequences"[6]

Alcohol use disorders
The NIAAA define AUD as "a chronic relapsing brain disorder characterized by an impaired ability to stop or control alcohol use despite adverse social, occupational, or health consequences" [6].

Subhani M, *et al. BMJ Open* 2021; 11:e054954. doi: 10.1136/bmjopen-2021-054954

SP-Focus Group Guide WP1 V2.0

Focus group Guide participants
Work Package 1 (WP1)
Version 2.0 Date:14/10/2020

Study title: **Does knowledge of liver fibrosis affect high-risk drinking behaviour (KLIFAD)? A feasibility randomised controlled trial**

To begin

Welcome to the focus group session. Thanks for taking the time to join us to talk about liver disease screening.

You were invited here today because you attended a liver scan appointment and were given your level of risk for liver disease using a Fibroscan machine. We would like to understand how to provide the best experience for patients undergoing the scan. This includes how the person operating the Fibroscan machine discusses the scan itself and then delivers the results of the scan to patients. We will ask you to read through a script we have prepared to help operators talk through the scan and also a document that provides patients with their results.

Everyone's risk of liver disease may be different. Because everyone has very different life experiences, there are no wrong answers to these questions, but rather different points of view. Please feel free to share your point of view even if it differs from what others have said. Keep in mind that we're just as interested in negative comments as positive comments, and at times the negative comments are the most helpful.

Logistics

- Focus group will last about 2 hours
- Feel free to move around
- Where is the bathroom? Exit?
- Help yourself to refreshments

Ground Rules

- Hope that everyone feels comfortable enough to participate.
- Information provided in the focus group must be kept confidential
- Stay with the group and please don't have side conversations
- Turn off mobile phones if possible
- This is an opportunity to help contribute to the treatment of liver disease!

You've probably noticed the microphone. I'm tape recording the session because I don't want to miss any of your comments. People often say very helpful things in these discussions and I can't write fast enough to get them all down.

If you talk about anyone else during the focus group by name (such as a friend or member of staff) – then we will keep their name anonymous when we write up the results by providing them with a false name. Likewise (the participant) we will also keep your identity anonymous during the write-up by giving you a false name in any reports resulting from this study

Are you okay with this? Do you have any questions?

- Answer any questions they have
- If they do not want to participate, thank them for their time and escort them out of the venue. If they have participated via telephone or over video conferencing – finish the call.

Beginning the focus group

Start recording the interview on the Dictaphone.

Firstly, I want you to think back to your liver scan appointment.

1. Did you understand why you were undergoing a fibroscan and what the scan involved?
2. What was your experience of the scan? Was there anything about the way the operator conducted the scan or talked to you about the scan that you liked/disliked/found helpful?
3. After the scan, what information were you provided with? Including your results, any feedback from the scan operator, and any other information about liver disease?
    a. Was any of this difficult to understand? What information did you find most helpful?
4. Did the scan and/or scan results prompt you to make some changes to improve your liver health?
    a. If you received normal scan results, did you still want to make lifestyle changes?

Now I'd like us to spend the rest of the session today reviewing the documents in front of you. Please take some time to read through these documents and write any thoughts you have about the wording or how the information is presented on the document.

Provide participants with pens
Give participants approximately 10-15 minutes to read through script and fibroscan results

Let's review the operator script. Imagine you were receiving this information from a fibroscan operator.

1. Do you understand the information presented in the script?
    a. What did you like/dislike about the script? What information was helpful/unhelpful? Was anything unclear?
2. Was there any information you felt was missing or that you think would make a useful addition to the script?

   a. Do you have any suggested changes or improvements to the script?

Now let's review the fibroscan result documents. There are three different results a patient can receive, depending on their liver stiffness. Imagine you were receiving this information from a fibroscan operator.

1. Do you think the results made sense for each level of liver disease stiffness?
   a. Did you understand the information? What information was helpful/unhelpful? Was anything unclear?
2. How did the documents make you feel?
   a. Did anyone have a negative reaction/positive reaction?
3. Did you like the way the results were presented (e.g. graphically, visually)?
   a. What would you change? Would you prefer the results to be presented as a value, on a scale, on a graph etc.?
4. Would you feel confident knowing what your result was and how to go about making lifestyle changes from this information?
   a. If not, why and what could we include that would help improve your confidence? Do you think the results documents would need explaining further by the operator?
5. Does anyone have additional thoughts about a specific result document (normal, likely fibrosis, likely cirrhosis)?
   a. Do you think the information reflects the level of risk and need for behaviour change?
6. Is there any other information we should include in the results document?
   a. Do you have any suggested changes or improvements to the results?

Close

Okay, that reaches the end of the questions I wanted to ask today. Is there anything else you wanted to add or talk about that we didn't talk about today?

If you're okay to end the focus group there, I'll switch the Dictaphone off, thank you!

Debriefing

- Thank you for speaking to us.
- Provide participants with a sheet which outlines the range of services etc, go through it with them. If there is any particular service/resource that they have expressed an interest in – then signpost them to it.
    - If they have participated via telephone– a state that they can be sent this via email if this wish or it can be read out to them.
- Thank them again, and ask if they are feeling okay to leave the building/ or hang up/exit the call.

SP-Change model questionnaire (CMQ) V1.0

Change model questionnaire

Work package 1 (WP1) V1.0 26/10/2020

Study title: Does knowledge of liver fibrosis affect high-risk drinking behaviour (KLIFAD)? A feasibility randomised controlled trial

Your doctor may have asked you to cut down how much alcohol you are drinking. Please find the statement that best describes the way you feel right now about cutting down your alcohol use to the amount the research team recommends

- ☐ I am continuing to drink at the same level and right now I am not considering reducing how much I drink
- ☐ I am continuing to drink at the same level but and right now I am considering reducing how much I drink
- ☐ I am continuing to drink at the same level but I am planning to reduce how much I drink
- ☐ Right now I have reduced how much alcohol I drink, and have maintained this for less than six months
- ☐ Right now I have reduced how much alcohol I drink, and have maintained this for more than six months

SP- Qualitative interview guide

Qualitative interview Guide
Work package 3 (WP 3) Feasibility RCT

***Study title:* Does knowledge of liver fibrosis affect high-risk drinking behaviour (KLIFAD)? A feasibility randomised controlled trial**

To begin

- Go over the study information again with the participant:
  - Thank you for coming to/agreeing to take part in the interview today…
  - Explain what will happen:
  - 'You'll be asked brief questions about your experience of taking part in the KLIFAD study and some questions about how you felt about taking part in this study and how it might have had an impact on you".
  - There are no 'right' or 'wrong' answers – I am not here to judge you, but to listen to your experiences as everyone's experience is valuable.
  - You can tell us as little or as much information as you want to during this interview, it is kept confidential in the research team. We may use a transcription service, but they are required to sign a confidentiality agreement and identifiers are removed from the typed-up transcript.
  - You can pause or stop the interview at any time if you want a break, you feel uncomfortable or don't want to continue with the interview.
  - After the interview, I will provide you with information about services and resources – that you may find useful if you have any concerns about what you have told us.
- Are you okay with all this? Do you have any questions?
  - Answer any questions they have
  - If they do not want to participate, thank them for their time and escort them out of the venue. If they have participated via telephone or over video conferencing – finish the call.

- Note: We will ask our PPI group about whether to include clarification of specific terms at this point. For example, relapse or lapse or teetotal/sober etc to ensure we ask questions in the participant's preferred way of talking about their alcohol use.
- If you talk about anyone else during the interview by name (such as a friend or member of staff) – then we will keep their name anonymous when we write up the results by providing them with a false name. Likewise (the participant) we will also keep your identity anonymous during the write-up by giving you a false name in any reports resulting from this study
- If you are satisfied with this, please confirm that you still consent to take part.
  - They will have already consented to take part when they signed up. Check you have received this consent (if was by e-mail or post).
  - If unsatisfied and does not want to take part – thank them for their time and guide them out of the venue/end the call.

Subhani M, *et al. BMJ Open* 2021; 11:e054954. doi: 10.1136/bmjopen-2021-054954

Beginning the interview

Start recording the interview on the Dictaphone.

Here we can ask an introductory question to establish some rapport.

Your experience of the KLIFAD study

Q. Have you ever taken part in a research study before?

Q. Can you take me through what you remember about the KLIFAD study? (If they get into specifics of the results…. We'll touch on that later, for now, I'd like you to think about your experience of the scan process as a whole, for example how you felt about the scan or the staff who scanned you.)

Q. Overall, how do you feel about taking part in the KLIFAD study?

Follow up questions: If positive feedback: What did you particularly like?

If negative feedback: What did you not like/thought could be different?

Q. In regard to the fibroscan, did you understand why you were invited to have this scan? Did the staff give you enough information about the scan? Was there anything about the whole process you liked/didn't like?

Q. Where did you watch the stories? Did you watch it with anyone else? What was your response to them?

Your feelings about getting the KLIFAD study

Q. Can you tell me what you remember about your fibroscan scan result?

Follow-up questions: Can you remember the specific value, scale, what the value meant (potential liver disease etc)? Was the result explained clearly, did you understand it? Can

you think of ways to improve how we give people their scan results? Is there anything else you think would be helpful to know when you receive your scan result?

Q. Do you remember how you felt when you first got your fibroscan result? Explore their thoughts and feelings here by using reflection 'So, I'm hearing that you felt confused and a bit frightened'. Also can use follow-up questions if appropriate e.g., Can you talk a bit more about why you felt scared? Can you describe your feeling of relief? Etc.

Q. What did it feel like watch stories describing other people's experiences of receiving a fibroscan? Follow up questions: Which stories can you remember accessing? Can you describe any ways in which these made an immediate impact on you? Can you describe any ways in which these have made a longer-tem impact on you? Did you learn anything from the stories?

Q. Did you discuss the KLIFAD study with anyone?

Follow up questions: What part did you talk about? (Scan/story/both?). Who did you talk to about it? How did they feel about it? If they didn't talk to anyone about it, ask why they didn't

Q. Now that a bit of time has passed, how do you feel about taking part in the KLIFAD study?

Your use of alcohol since you took part in the KLIFAD study

Q. Can you talk about your use of alcohol at a few different time points? It may be hard to remember this far back so sometimes it's helpful to look at a calendar and plot out some key dates (e.g. birthdays, trips away) that can help you remember.

1. Your use of alcohol (if any) just before you had your fibroscan result
2. Your use of alcohol (if any) on the day or days after you had your fibroscan result
3. Your use of alcohol (if any) two weeks after you had your result
4. Your use of alcohol (if any) over the last month

Q. Do you think the KLIFAD study changed your use of alcohol in any way?

If yes: explore, how, why do they think it affected it. If no: invite them to talk about that.

Explore if they sought out additional supports e.g. AA

Follow-up: Had you thought about changing before taking part in this study?

Q. If yes to changes, what were your main reasons for making these changes?

Q. If no, tell me more about why you didn't want to or didn't feel able to make changes at that time.

Follow-up questions: Was there anything that helped you make the changes? Was there anything that was a barrier to making changes?

Close

Okay that reaches the end of the questions I wanted to ask you. Is there anything else you wanted to add or talk about that we didn't talk about today?

If you're okay to end the interview there, I'll switch the Dictaphone off, thank you!

Debriefing

- Thank you for speaking to us.
- How are you feeling – is there anything in the interview has troubled you or upset you?
- Provide participant with sheet which outlines range of services etc, go through it with them. If there is any particular service/resource that they have expressed an interest in – then signpost them to it.
    - If they have participated via telephone– state that they can be sent this via email if this wish or it can be read out to them.
- Thank them again, and ask if they are feeling okay to leave the building/ or hang up/exit the call.

1. Anthony WA. Recovery from mental illness: The guiding vision of the mental health service system in the 1990s. *Psychosocial Rehabilitation Journal.* 1993;16(4):11-23.
2. Rennick-Egglestone S, Morgan K, Llewellyn-Beardsley J, et al. Mental Health Recovery Narratives and Their Impact on Recipients: Systematic Review and Narrative Synthesis. *Canadian journal of psychiatry Revue canadienne de psychiatrie.* 2019;64(10):669-679.
3. Witkiewitz K, Wilson AD, Pearson MR, et al. Profiles of recovery from alcohol use disorder at three years following treatment: can the definition of recovery be extended to include high functioning heavy drinkers? *Addiction (Abingdon, England).* 2019;114(1):69-80.
4. <Policy report - A vision of recovery_ UKDPC recovery consensus group.pdf>.
5. Llewellyn-Beardsley J, Rennick-Egglestone S, Callard F, et al. Characteristics of mental health recovery narratives: Systematic review and narrative synthesis. *PloS one.* 2019;14(3):e0214678.
6. Alcoholism NIoAAa. Helping patients who drink too much: a clinician’s guide, updated 2005 edition. Rockville: National Institutes of Health. 2005.
