# Supplementary material for: Does knowledge of liver fibrosis affect high-risk drinking behaviour (KLIFAD)? protocol for a feasibility randomised controlled trial
Source: BMJ Open. 2021 Nov 3;11(11):e054954. doi: 10.1136/bmjopen-2021-054954 (PMC8572412; doi:10.1136/bmjopen-2021-054954)
Supplement: Supplementary data [file bmjopen-2021-054954supp002.pdf]

Nottingham Recovery Network

Assessment Form

Clean Slate
Criminal Justice Substance Misuse Service

OFFICE USE ONLY

Date received:<br>Referred by:<br>If SELF, how did they hear about the service:		Client Id:<br>Assessed by:<br>Assessment location:<br>Date:
Specific risk / need identified:	Probation<br>Y / N	

CLIENT DETAILS

GDPR: There is a privacy notice in each room - please read it

DRUG ☐ If drug(s) please state type:
DRUG & ALCOHOL ☐
ALCOHOL ☐

**Title:** Mr / Mrs / Ms / Miss / other (please state)<br>**First name:** **Surname:**<br>Prefers to be known as:<br>**Gender:** Male ☐ Female ☐ Other ☐ Not specified ☐<br>**Date of Birth:** **Age:**<br>**Address:**<br>**Postcode:**<br>**Home Tel:**<br>**Mobile Tel:**<br>**Email address:**	**G.P Name:**<br>Address:<br>Tel:<br>Currently receiving treatment? Y / N<br>Currently on prescribed medication? Y / N<br>Seen by GP in last month? Y / N<br>GP aware of substance misuse? Y / N<br>**Pharmacy current/preferred:**
**Permission to be contacted:** Home Visit ☐ Letter ☐ Telephone ☐ Text ☐ Email ☐	
**Emergency Contact:**	**Telephone:**
**Medication:** Current ☐ Recent Past ☐ Past ☐ None ☐<br>Type & Dosage – List<br>...<br>...<br>Prescribed by:	**Known Allergies:** Y / N<br>List:<br>..<br>..<br>..
**Barriers to Accessing Treatment? (include any disabilities)**	**Preferred Language:**<br>**Is an interpreter required** Y / N

NRN Assessment – Jan 2019 *1*
