# Supplementary material for: Does knowledge of liver fibrosis affect high-risk drinking behaviour (KLIFAD)? protocol for a feasibility randomised controlled trial
Source: BMJ Open. 2021 Nov 3;11(11):e054954. doi: 10.1136/bmjopen-2021-054954 (PMC8572412; doi:10.1136/bmjopen-2021-054954)
Supplement: Author's manuscript [file bmjopen-2021-054954.draft_revisions.pdf]

BMJ Open is committed to open peer review. As part of this commitment we make the peer review history of every article we publish publicly available.

When an article is published we post the peer reviewers' comments and the authors' responses online. We also post the versions of the paper that were used during peer review. These are the versions that the peer review comments apply to.

The versions of the paper that follow are the versions that were submitted during the peer review process. They are not the versions of record or the final published versions. They should not be cited or distributed as the published version of this manuscript.

BMJ Open is an open access journal and the full, final, typeset and author-corrected version of record of the manuscript is available on our site with no access controls, subscription charges or pay-per-view fees (http://bmjopen.bmj.com).

If you have any questions on BMJ Open's open peer review process please email info.bmjopen@bmj.com

BMJ Open

Does knowledge of liver fibrosis affect high-risk drinking behaviour (KLIFAD): Protocol for a feasibility randomised controlled trial

Journal:	*BMJ Open*
Manuscript ID	bmjopen-2021-054954
Article Type:	Protocol
Date Submitted by the Author:	01-Jul-2021
Complete List of Authors:	Subhani, Mohsan; University of Nottingham, Nottingham Digestive Diseases Biomedical Research Centre (NDDC); Nottingham University Hospitals NHS Trust, NIHR Nottingham Biomedical Research Centre<br>Jones, Katy; University of Nottingham School of Medicine, Division of Psychiatry & Applied Psychology<br>Sprange, Kirsty; University of Nottingham, Nottingham Clinical Trials Research Unit<br>Rennick-Egglestone, Stefan; University of Nottingham School of Health Sciences,<br>Knight, Holly; University of Nottingham,<br>Morling, Joanne; University of Nottingham<br>Enki, Doyo; University of Nottingham, Medical Statistics<br>Ryder, Stephen; Nottingham University Hospitals NHS Trust, Nottingham Digestive Diseases Biomedical Research Centre (NDDC), School of Medicine
Keywords:	Hepatology < INTERNAL MEDICINE, Gastroenterology < INTERNAL MEDICINE, PUBLIC HEALTH

SCHOLARONE™
Manuscripts

I, the Submitting Author has the right to grant and does grant on behalf of all authors of the Work (as defined in the below author licence), an exclusive licence and/or a non-exclusive licence for contributions from authors who are: i) UK Crown employees; ii) where BMJ has agreed a CC-BY licence shall apply, and/or iii) in accordance with the terms applicable for US Federal Government officers or employees acting as part of their official duties; on a worldwide, perpetual, irrevocable, royalty-free basis to BMJ Publishing Group Ltd ("BMJ") its licensees and where the relevant Journal is co-owned by BMJ to the co-owners of the Journal, to publish the Work in this journal and any other BMJ products and to exploit all rights, as set out in our licence.

The Submitting Author accepts and understands that any supply made under these terms is made by BMJ to the Submitting Author unless you are acting as an employee on behalf of your employer or a postgraduate student of an affiliated institution which is paying any applicable article publishing charge ("APC") for Open Access articles. Where the Submitting Author wishes to make the Work available on an Open Access basis (and intends to pay the relevant APC), the terms of reuse of such Open Access shall be governed by a Creative Commons licence – details of these licences and which Creative Commons licence will apply to this Work are set out in our licence referred to above.

Other than as permitted in any relevant BMJ Author's Self Archiving Policies, I confirm this Work has not been accepted for publication elsewhere, is not being considered for publication elsewhere and does not duplicate material already published. I confirm all authors consent to publication of this Work and authorise the granting of this licence.

For peer review only

Does knowledge of liver fibrosis affect high-risk drinking behaviour (KLIFAD): Protocol for a feasibility randomised controlled trial

Mohsan Subhani[1,2], Katy A Jones[4], Kirsty Sprange[5], Stefan Rennick-Egglestone[6], Holly Knight[3], Joanne R Morling[1,2,3], Doyo G Enki[7], Andrew Wragg[2], Stephen D Ryder[1,2]

Affiliations

[1]Nottingham Digestive Diseases Biomedical Research Centre (NDDC), School of Medicine, University of Nottingham

[2]NIHR Nottingham Biomedical Research Centre, Nottingham University Hospitals NHS Trust and the University of Nottingham, Nottingham, UK

[3]Division of Epidemiology and Public Health, University of Nottingham, Nottingham, UK

[4]Academic Unit of Mental Health and Clinical Neuroscience, School of Medicine, C24 Institute of Mental Health, University of Nottingham

[5]Nottingham Clinical Trials Unit, University of Nottingham

[6]School of Health Sciences, Institute of Mental Health, University of Nottingham

[7]School of Medicine, University of Nottingham

Acronym

KLIFAD

Corresponding Author

Mohsan Subhani

Email: mohsan.subhani@nottingham.ac.uk Telephone: 0044 7572612429

Research reference numbers

IRAS Number:	273765
SPONSOR Number:	124481/2020
FUNDER Number:	RfPB NIHR201146
ISRCTN	16922410
Protocol version	V1.4

Sponsor

Nottinghamshire Healthcare NHS Foundation Trust

research@nottshc.nhs.uk

Funder

National Institute for Health Research

Scheme: Research for Patient Benefit (RfPB)

For peer review only - http://bmjopen.bmj.com/site/about/guidelines.xhtml

Abbreviations

ARLD	Alcohol-related liver disease
ARVS	Alcohol recovery video stories
AUD	Alcohol use disorders
AUDIT	Alcohol Use Disorder Identification Test
BRC	Biomedical Research Centre
CONSORT	Consolidated Standards of Reporting Trials
COREQ	Consolidated Criteria for Reporting Qualitative Studies
GCP	Good Clinical Practice
Kpa	Kilopascal
NDTMS	National Drug Treatment Monitoring System
NDDC	Nottingham Digestive Diseases Centre
NHS	National Health Service
NICE	National Institute of Clinical Excellence
NRN	Nottingham Recovery Network
NUH	Nottingham University Hospital
PIS	Patient information sheet
PPI	Patient and public involvement
RCT	Randomised Controlled Trial
REC	Research Ethics Committee
SADQ	Severity of Alcohol Dependence Questionnaire
UK	United Kingdom
WoSRES	West of Scotland Research Ethics Service
WP	Work package

For peer review only

For peer review only - http://bmjopen.bmj.com/site/about/guidelines.xhtml

Abstract

Introduction

Heavy drinkers in contact with alcohol services do not routinely have access to testing to establish the severity of potential liver disease. FibroScan can provide this information. A recent systematic review suggested providing feedback to patients based on markers of liver injury is an effective way to reduce harmful alcohol intake. This randomised control trial aims to establish the feasibility of conducting a larger national trial to test the effectiveness of FibroScan advice and alcohol recovery video stories in changing high-risk drinking behaviour in community alcohol services common to United Kingdome (UK) practice.

Methods and analysis

The feasibility trial consists of three work packages (WP). **WP1:** To draft a standardised script for FibroScan operators to deliver liver disease-specific advice to eligible participants having FibroScan. **WP2:** To create a video library of alcohol recovery video stories for use in the feasibility RCT (WP3). **WP3:** To test the feasibility of the trial design, including the FibroScan script and video stories developed in WP1 and WP2 in a 1:1 randomised trial in community alcohol services. Semi-structured interviews will be conducted at six months follow up for qualitative evaluation. Outcomes will be measures of the feasibility of conducting a later larger RCT related to participant recruitment and follow-up, intervention delivery, including the use of the KLIFAD FibroScan scripts and videos, clinical outcomes and the acceptability and experience of the intervention and trial-related procedures. Data analysis will primarily be descriptive to address the feasibility aims of the trial. All proposed analyses will be documented in a Statistical Analysis Plan.

Ethics and dissemination

The trial received favourable ethical approval from the West of Scotland Research Ethics Service (WoSRES) on 20th January 2021, REC reference: 20/WS/0179. Results will be submitted for publication to a peer-reviewed journal.

Trial registration number

ISRCTN16922410, Pre-results

Keywords: Alcohol. Fibroscan. Alcohol related liver disease. Alcohol recovery stories.

For peer review only - http://bmjopen.bmj.com/site/about/guidelines.xhtml

Strengths and limitations of the trial

- Integration of non-invasive testing to establish the severity of potential liver disease into community alcohol settings.
- Outcomes will help to understand if normal FibroScan results provide false reassurance to participants.
- Creation of alcohol recovery stories videos to use as peer support.
- Due to the nature of the trial intervention, blinding is not possible.
- Single centre study could limit generalisation.

For peer review only

For peer review only - http://bmjopen.bmj.com/site/about/guidelines.xhtml

Introduction

Alcohol-related liver disease (ARLD) is the most common cause of cirrhosis in UK, and mortality from ARLD has risen significantly in the last three decades. It is now the second most common cause of working life years lost in men and fifth in women [1,2]. Europe has one of the highest prevalence of Alcohol Use Disorders (AUD) involving 14.8% of men and 3.5% of women[2]. Around 25% of the United Kingdom (UK) population drink above the recommended level, and 10% are harmful drinkers; the total per capita pure alcohol intake (age ≥15year) is 11.4 litres/annum averaging 175 grams of alcohol per week per person [3]. Approximately 20-30% of lifelong drinkers develop liver cirrhosis, and the risk is even higher (35%) among harmful drinkers [4,5].

ARLD causes no symptoms in its earlier stages; indeed, patients are often unaware they have serious physical health problems until they present with the complications of cirrhosis for example; ascites (building fluid into abdomen), jaundice, encephalopathy (altered mental state), variceal bleed (bleeding from upper gastrointestinal tract) and liver failure . When the opportunity for treatment and recovery of liver health are significantly reduced [1,5,6]. It is estimated that the cost to the UK of alcohol on health is £3.5 billion per year [3,7], consuming 3.6% of the National Health Service (NHS) annual budget [8]. In England, in 2018 there were 5,698 alcohol-specific deaths, the alcohol-specific age-standardised death rate/100,000 was 11.9 (male=16.4 female=7.6), Nottingham has one of the highest (total=18.6, male=26.8, female 10.2) alcohol-specific age-standardised death rate/100,000 in the country [9]. A recent study from the United States (US) predicted a 75% increase in age-standardised annual mortality and a 65% increase in decompensated cirrhosis due to ARLD over the next two decades[10].

Systematic reviews of Randomised Controlled Trials (RCTs) have established that delivering brief advice about alcohol to harmful drinkers helps them reduce their alcohol consumption[11,12]. Most studies were conducted in primary care settings where the prevalence of liver disease is likely to be markedly lower than in specialist alcohol treatment services. In alcohol services, where high levels of physical and psychological dependence on alcohol are frequent, National Institute of Clinical Excellence (NICE) guidelines state adults with high levels of alcohol dependency should be assessed and offered intensive structured community-based interventions (with or without medical therapy) as these provide the best chance of achieving and maintaining abstinence from alcohol[13]. Most clinical services in the UK are based on these principles. Individual programmes vary by locality with many of these services delivered by non-NHS providers. Despite brief advice and other alcohol-related interventions delivered in clinical practice for over two decades, mortality and morbidity due to alcohol misuse continue to rise in the UK[3]. There is a pressing need to optimise existing interventions to reduce harmful alcohol intake and examine effective alternative options.

Early diagnosis of liver fibrosis provides an opportunity to intervene and reduce or stop alcohol intake. This is known to be the most effective way of preventing liver disease progression [14]. FibroScan has been used in primary care (General Practice) settings to detect liver disease in populations identified as having liver disease risk (heavy drinkers and those with type 2 diabetes). These studies showed that screening asymptomatic individuals based on risk for liver disease doubled the rates of liver cirrhosis diagnosis in the primary care populations studied[15,16]. Moreover, a recent systematic review suggested providing feedback to patients based on markers of liver injury is an effective way to reduce harmful alcohol intake[17]. The addition of recovery stories helps one's mental health illness and addiction recovery [18,19]. The

For peer review only - http://bmjopen.bmj.com/site/about/guidelines.xhtml

peer support from people who have recovered from alcohol misuse had been proven beneficial in modifying high risk drinking behaviour[20].

This trial aims to investigate the feasibility and acceptability of conducting an RCT in community specialist alcohol services settings run by Nottingham Recovery Network (NRN) and to test the acceptability of trial interventions (FibroScan and Alcohol Recovery Video Stories, ARVS).

The objectives of this trial are.

1. To establish a standardised script framework for FibroScan operators to deliver liver disease-specific advice to participants having a FibroScan.
2. To develop a collection of video stories describing how patients have responded to receiving a FibroScan score.
3. To test the intervention (FibroScan plus feedback and ARVS) in a feasibility randomised control trial.
4. To perform a qualitative evaluation of objectives 1-3 to inform a large-scale RCT.
5. Determine the feasibility of recruitment and randomisation to a large-scale RCT.
6. Refine the eligibility criteria for a future large-scale RCT.
7. Determine the acceptability of randomisation to patients and healthcare workers.
8. Determine the relevance and acceptability of the trial intervention to patients and healthcare workers.
9. Determine the acceptability of the trial procedures to patients and healthcare workers.
10. Assess the ability of community alcohol services to deliver the intervention.
11. Assess training and support needs for community alcohol services keyworkers for delivering the intervention.
12. Assess follow-up and outcome completion rates.
13. To develop change model for FibroScan plus feedback and ARVS

Methods and analysis:

KLIFAD is a parallel design feasibility RCT. The trial will be conducted in a single centre in the UK, carried out at three community alcohol services in Nottingham (the Wellbeing Hub, Edwin House and the Primary Care Alcohol Clinic run by the Nottingham Recovery Network) hosted by Framework and Nottingham Recovery Network (NRN) and working in partnership with Nottinghamshire NHS Foundation Trust. The KLIFAD trial consists of three work packages (WP) (Figure 1).

Work Package 1 (WP1)

WP1 aims to design a standardised script framework for FibroScan operators to deliver liver disease-specific advice to participants having FibroScan as part of the feasibility RCT (WP3). A prototype script for FibroScan has been created in consultation with the existing KLIFAD Patient Public Involvement (PPI) group covering three ranges of FibroScan scores, normal ≤7 Kilopascal (kPa), intermediate fibrosis 8-15 kPa and advance fibrosis ≥15 kPa. The sample of these scripts are provided in supplementary material (SP) and the trial flow chart in Figure 2.

We will organise separate participant and FibroScan operator focus groups to collect feedback on the prototype scripts. The participant focus group will allow us to investigate the key messages to be included in the script and feedback, as well as considering how best to present

For peer review only - http://bmjopen.bmj.com/site/about/guidelines.xhtml

the FibroScan results (e.g., graphically, in the text). The FibroScan operator focus group to investigate implementations in clinical practice. In addition, to evaluate the stages of change, a validated readiness to change model will be piloted[21].

Each focus group will include 5-8 participants and will last for a maximum of 2 hours. Depending upon the latest Covid-19 guidelines the focus group will be either virtual or face-to-face. A topic guide will be used (SP-Focus Group Guide WP1 V2.0). We aim to arrange two participant focus groups and one FibroScan operator focus group. The focus groups will be facilitated by two members of the research team. Examples of questions include:

a) If you were a participant in the trial, would the script make sense to you?
b) Are there any parts of the script that you do not understand, and if so, why?
c) What is the best way to present the results of the FibroScan?

Eligible participants (Table 1) will be identified and recruited through multiple channels. For example, via existing patient forums at all three recruitment settings, the KLIFAD PPI group, by offering information to patients self-presenting to any of the study treatment settings, snowball methods, and via Black, Asian and minority ethnicity/Framework PPI groups. The focus group meeting will be recorded and transcribed verbatim either by automated software or an independent sponsor approved transcriber. After the first participant focus group the FibroScan script will be edited considering feedback and a second focus group will then be held to review iterated scripts. The final scripts will be sent via email to participants of focus groups for any final thoughts. We will then organise a FibroScan operator focus group of key alcohol workers working at any of the recruitment settings who are willing to give informed consent, to discuss any specific implementation issues.

After the focus group, we will collect participant feedback on the change model (SP-Change model questionnaire (CMQ) V1.0) to get an initial sense of the applicability of readiness to change following discussion about the scripts.

For peer review only - http://bmjopen.bmj.com/site/about/guidelines.xhtml

Work-package-1	
**Inclusion criteria**	**Exclusion criteria**
A person age ≥18 years	Other primary substance misuse even where alcohol is a factor
Primary problem of alcohol misuse	Lacks capacity to confirm consent
Had fibroscan in past	
**Work-package-2**	
**Inclusion criteria**	**Exclusion criteria**
A person age ≥18 years	Lacks capacity to confirm consent
Primary problem of alcohol misuse	
Had fibroscan in past	
A with lived experience of alcohol problems	
A person Willing to consent to the recording and public use of video recording	
**Work-package-3 the randomisation phase**	
**Inclusion criteria**	**Exclusion criteria**
A person age ≥18 years	Other primary substance misuse even where alcohol is a factor
Primary problem of alcohol misuse	Lacks capacity to confirm consent
	Referrals from driving offences and student referrals[a]
	Out of area clients at Edwin house[b]
	Participants unable to comply with study procedures

Table 1: KLIFAD trial eligibility criteria

[a]As these individuals are essentially not self-presenting, may have different motivation and have lower overall levels of alcohol use and so are substantially lower risk of having liver disease

[b]In whom we cannot obtain follow up data due to lack of follow up availability

For peer review only - http://bmjopen.bmj.com/site/about/guidelines.xhtml

Work Package Two (WP2)

WP2 aims to create a video library of ARVS from people with a history of alcohol misuse. These ARVS will be used in the feasibility RCT (WP3).

Receiving mental health recovery stories can provide benefits to some people experiencing mental health distress [18,22,23], and the effectiveness of mental health recovery stories as an intervention to increase quality of life has been examined in a clinical trial[24]. However, equivalent evidence is not available for the impact of ARVS. So that we can explore the impact of stories of recovery from alcohol misuse, in WP2 we will develop a set of recovery stories from participants who have successfully overcome their alcohol misuse. These videos will be peer-reviewed by the KLIFAD PPI group which will include input from Nottingham University Hospitals NHS Trust (NUH) Black, Asian and minority Ethnic patient and public involvement Group. Based on feedback the videos will then be edited ready for use in the feasibility RCT (WP3). All edits will be agreed upon with the story narrators.

For each narrator, we will follow their preference to create either:

- A recovery story that starts with an open-ended question where narrators have the liberty to tell their story without interruption *or*
- A recovery story in which the participant is asked a set of standard questions.

Drinking history and last FibroScan reading will be recorded at the start. Eligible participants (Table 1) will be recruited through the channels used in WP1. Those who took part in WP1 will also be invited to take part in WP2. A purposive sample based on demographic and liver disease severity of 6-9 individuals will be selected[25]. We will arrange a meeting with the KLIFAD PPI group to discuss what makes a video impactful. The outline of WP2 is given in Figure 2.

The ARVS will be recorded either at NDDC Biomedical Research Centre Nottingham University Hospital, the University of Nottingham, or the participant's usual place of residence. Each video will be of 2-5-minute duration. Videos will be titled based on FibroScan score (low-risk, medium and high-risk score). Videos will be subtitled and depending on the final video format after the feedback we envisage adding a photograph of the storyteller and a short-associated text on the title page. The video stories will be brought together in a single tablet computer-based package from which the participant will be able to choose their most preferred video after receiving a FibroScan score. Collaborative work between a clinician and patient can make a significant impact on the recovery process [26] and hence in some videos, and with consent by narrators, we will include sections of a video narrated by a clinician the narrator has worked with.

All video stories recorded as part of the KLIFAD trial will have peer review by the study team and KLIFAD/Black, Asian and ethnic minority PPI groups. The videos will be shown in the same format that they would be used in WP3.

Work Package 3 (WP3)- Feasibility RCT

A feasibility RCT of parallel groups (1:1) will compare usual care (assessment and entry into an alcohol reduction programme which does not include information on liver disease severity) to usual care plus feedback from the FibroScan and ARVS. The eligibility for WP3 is provided in Table 1 and the attached flow chart (Figure 3).

For peer review only - http://bmjopen.bmj.com/site/about/guidelines.xhtml

Intervention Group

Participants randomised to the intervention arm will receive a FibroScan, feedback on FibroScan results and watch ARVS immediately after. The ARVS will be made available should a participant wish to watch them later.

Control group

Participants randomised to the control arm will continue with standard treatment (usual care) provided at the three treatment settings. The participants in this arm will be offered FibroScan at 6 months.

As part of standard treatment, the recruitment settings provide different types of interventions to participants in line with the National Drug Treatment Monitoring System Dataset (NDTMS) and Public health England (PHE) guidelines [27]. Existing treatment programmes can run for up to 12-weeks.

For adult drug and alcohol services there are three main categories of standard intervention (usual care) delivered by the NRN:

a) Psychological: which includes motivational interventions, family and social network interventions, and cognitive and behavioural based relapse prevention interventions (substance misuse specific).
b) Recovery Support: which includes 12 step work and counselling.
c) Pharmacological: which involves prescribing medication for drug and/or alcohol relapse prevention support. For example, naltrexone, acamprosate, disulfiram as part of alcohol or opioid relapse prevention therapy and Chlordiazepoxide for acute alcohol withdrawal.

Specific treatment programmes are started after an initial assessment and based on the participant's needs. The duration of contact with services varies, most participants stay with services for 12 weeks, some get discharged early, and a few stays longer than six months.

Methods

Sample size

As this is a feasibility study, a formal sample size calculation for between-group comparisons of a primary outcome is not appropriate. However, we aim to approach 40 eligible participants per month. Assuming a 50% consent rate we anticipate randomising 20 participants per month (10 per month per arm) for a recruitment period of six months we estimate a drop-out rate of 20% to within a 95% confidence interval of ±7%. Assuming a non-differential follow-rate of 80%, this target sample size should provide follow-up outcome data on a minimum of 48 participants in each of the two arms.

Randomisation

The participants will be individually allocated on a 1:1 ratio using minimisation with a probabilistic element. The minimisation variables will be age, gender, ethnicity, and severity of alcohol misuse based on the Severity of Alcohol Dependence Questionnaire (SADQ) score.

Schedule of visits

Baseline

The baseline visits will be on the day when the participant starts standard treatment at any recruitment setting. At this visit written informed consent will be given by participants and

For peer review only - http://bmjopen.bmj.com/site/about/guidelines.xhtml

For peer review only

participants will be randomised to the intervention or control group. Participants in both arms will have an initial detailed assessment (SP-NRN assessment form Supplementary Material) as part of their standard care. This includes the collection of baseline demographic and clinical data (e.g., age, gender, ethnicity). Participants randomised to the control arm will continue with usual care while participants randomised to the intervention arm will have the usual care and FibroScan followed by standardised script feedback with ARVS watched immediately after the FibroScan result.

Three months

This visit will be part of usual care no research specific activity will be carried out. The research data will be extracted from routinely collected data from three treatment settings.

Six months

This will be a telephone consultation or in-person appointment by the research team. Participants in the control arm will be offered a FibroScan after completion of outcomes. The six-month follow up is specifically to cover those who were lost to follow up at NRN from the treatment programme.

A detailed schedule of the visits is given in Table 2.

For peer review only

For peer review only - http://bmjopen.bmj.com/site/about/guidelines.xhtml

Study Activity	Baseline visit	3[a] Months	6[b] months
**Control group**			
Date & Time	Yes	Yes	Yes
Baseline consent	Yes	-	-
Fibroscan + Feedback	-	-	Yes
Watching video stories	-	-	Yes
Qualitative interview	-	-	Yes
Demographics	Yes	-	-
AUDIT score	Yes	Yes	Yes
SADQ score	Yes	Yes	Yes
Self-reported alcohol intake[c]	Yes	Yes	Yes
Breath alcohol test	Yes	Yes	Yes
Substance misuse other than alcohol	Yes	Yes	Yes
Data on feasibility outcomes	Yes	Yes	Yes
**Intervention group**			
Date & Time	Yes	Yes	Yes
Baseline consent	Yes	-	-
Fibroscan + Feedback	Yes	-	-
Watching video stories	Yes	-	-
Qualitative interview	-	-	Yes
Demographics	Yes	-	-
AUDIT score	Yes	Yes	Yes
SADQ score	Yes	Yes	Yes
Self-reported alcohol intake	Yes	Yes	Yes
Breath alcohol test	Yes	Yes	Yes
Substance misuse other than alcohol	Yes	Yes	Yes
Data on feasibility outcomes	Yes	Yes	Yes

Table 2: Work package 3 (feasibility RCT) schedule of visits and variables for data
(Alcohol Use Disorder Identification Test- AUDIT, Severity of alcohol dependence questionnaire-SADQ)

[a]3-months visit: this will be routine visit no trial-specific procedure will be carried out

[b]6 -months visit: will be a telephone consultation and/or if possible/required in person

The participant in the control group will be offered a Fibroscan at 6 months if they attend it will be in-person appointment

[c]Self-reported alcohol intake in gram and unites per week

For peer review only - http://bmjopen.bmj.com/site/about/guidelines.xhtml

Data collection

At Baseline, three and six months, the following data will be collected (Table 2)

- Demographics (including address, email address and contact number) This will be archived and kept separate from the main database.
- Alcohol Use Disorder Identification Test (AUDIT) scores.
- Severity of Alcohol Dependence Questionnaire (SADQ) scores.
- Self-reported alcohol intake (gram and unit per week).
- Substance misuse other than alcohol.
- Breath alcohol testing where participants are still attending.
  Breath alcohol testing is a strength of this study; most studies have relied on self-reporting of alcohol intake. This means we can correlate breath alcohol readings with self-reporting, providing substantial additional information.
- Data on feasibility outcomes (e.g., screening rate, recruitment rate, retention rate).

All the above measurements are part of routine outcomes data collected by all three recruitment settings, apart from the six-month data collected for those who are no longer in a treatment programme at six months. All three services included in this trial record all the above outcomes as part of the 12-week programme standard data set and report these to commissioners. Follow-up data is obtained at every attendance and includes the above dataset and breath alcohol testing.

Qualitative data

We will conduct one-to-one semi-structured interviews to evaluate participant's experiences of being part of the trial (e.g., "Overall, how do you feel about taking part in the KLIFAD study?") and any changes they may have made to their lives (e.g., "Do you think the KLIFAD study changed your use of alcohol in any way?"). The preliminary qualitative interview schedule topic guide is provided in supplementary material (SP- semi-structure interview). It will be piloted before use by the PPI group to check structure and wording of questions. A readiness to change model used in WP1 will also be piloted. Focus groups and interviews will be audio-recorded and transcribed by an independent transcriber approved by the sponsor for thematic analysis.

Health economics

Routine NHS data which is collected for the standard care 12-week treatment programmes will be used together with resources utilisation derived from the NHS digital linked data to derive healthcare costs and the potential benefits of the intervention.

Outcomes

The outcomes are designed to assess the feasibility and acceptability of the KLIFAD intervention and research processes to help inform a future large-scale RCT. The following outcomes will be reported:

- Recruitment rate.
- Retention rate.
- Consent rate.
- Acceptability of the intervention (FibroScan and ARVS).
- The willingness of participants to be randomised to trial arms.
- Acceptability of the intervention to patients.

For peer review only - http://bmjopen.bmj.com/site/about/guidelines.xhtml

- Participant adherence.
- Feasibility of outcome measures.

These feasibility outcomes will enable the study team to:

- Determine the best primary endpoint for the future definitive trial.
- Provide sample size estimates for the future definitive trial.
- Record ARVS which will contribute to the video library used in later large-scale RCT.

Statistical and data analysis plan

The analyses of the quantitative data will be in line with Consolidated Standards of Reporting Trials (CONSORT) guidelines for pilot and feasibility trials [28]. The primary descriptive analyses will be on an intention-to-treat basis (that is, participants are analysed in the group to which they were originally allocated). Data will be summarized using frequency (%), mean (SD) or median (IQR) depending on the distribution of the data. Summary measures will be presented along with their 95% confidence intervals whenever appropriate. Results of the data analysis will be presented using appropriate tables and graphs.

The study is not powered to investigate statistical significance between the two arms and as such no formal hypothesis testing will be undertaken for this study. As this is a feasibility study, no subgroup analysis is planned. However, the results of the feasibility variables will be presented by categories of different variables (age, gender, ethnicity, severity of alcohol misuse).

Different techniques will be followed to maximize the completeness of data collection (for example via staff training). The level of missing data will be assessed. This is especially useful for the proposed primary outcome variables. An interim analysis is not planned for this study, but the progress of the study will be reported to the oversight committee who can assess any concerns.

Thematic analysis of qualitative data will be conducted following Braun and Clarke's standard methods[29]. Care will be taken to integrate updated guidelines about thematic analysis including a transparent appreciation of researcher reflexivity[29]. If the trial management group feel the analysis requires external validity, a sample of transcripts identified by a random number generator with the codebook will be given to a researcher independent of the study. This will allow us to calculate the % agreement and Cohen's Kappa value (using criteria by Cohen, 1960)[30]. The Consolidated Criteria for Reporting Qualitative Studies (COREQ) will be used to ensure thorough and explicit reporting of qualitative data in reports and manuscripts for publication [31].

11Ethics and dissemination

Ethical approval

The trial received favourable ethical approval from the West of Scotland Research Ethics Service (WoSRES) on 20th January 2021, REC reference: 20/WS/0179.

Informed consent

All participants will provide a written or online (e-consent) informed consent before any research activities are initiated. A PIS written in plain language will be provided and it will be

For peer review only - http://bmjopen.bmj.com/site/about/guidelines.xhtml

ensured the participant has understood the trial information and had enough time to make an informed decision. The Site Investigator will be available to answer any questions about study participation.

Data handling and record-keeping

In compliance with the ICH/Good Clinical Practice guidelines, regulations and following the Nottinghamshire Healthcare NHS Foundation Trust SOPS, the Chief or local Principal Investigator will maintain all records and documents regarding the conduct of the study. These will be retained for at least 24 months or for longer if required. If the responsible investigator is no longer able to maintain the study records, a second person will be nominated to take over this responsibility. The routinely collected clinical data will be treated in the same way as other clinical case records are treated in the NHS following Nottinghamshire Healthcare NHS Foundation Trust's, the Government's, and funders' policies.

The Trial Master File and trial documents held by the Chief Investigator on behalf of the Sponsor shall be finally archived at secure archive facilities at the Nottingham Digestive Diseases Biomedical Research Centre (NDDC) at Nottingham University Hospital NHS Trust (NUHT). This archive shall include all trial databases and associated meta-data encryption codes.

An index will be created for the CRF and paper trial data before it gets stored. All online and IT-based data will be password protected and access will only be granted to people directly involved in trial and data analysis. All patient identifiable data will be anonymised with trial-specific participant number.

The information will be copied to the research database (REDCAP cloud) run by the NUHT. We will delete any information that identifies participant by the end of the KLIFAD study (currently expected October 2022). Moreover, we will ensure data security by following the UK data protection laws.

1Participant safety

There is a risk that being given a normal FibroScan result may provide false reassurance and encourage the participant to maintain their current level of harmful drinking or encourage them to drink more. It is also possible that a high reading will generate anxiety. The study is designed to minimise these risks by providing scripted feedback (WP1) and watching ARVS (WP2).

Cirrhosis diagnosis and FibroScan: It is anticipated that a small number of people will be identified who have previously unknown cirrhosis and so would be at risk of complications of liver disease. This will be mitigated by offering onward referral to out-patient Hepatology for all participants with a FibroScan reading >15KpA. This will be via contact with the participant's GP and would follow the current NUHT the Nottinghamshire adult liver disease stratification pathway for referral[32]. Some mitigation of this risk will be done via the feedback included in this trial which covers cirrhosis.

We cannot foresee any potential risks except possible emotional distress during participation in a focus group or semi-structured interview. Participants can choose to skip any question

For peer review only - http://bmjopen.bmj.com/site/about/guidelines.xhtml

that they prefer not to answer. If distress occurs during the study visit, we will ask the participant to take a break to recover or they can terminate the process. We do not expect that the study will cause any discomfort or pose any disadvantages, however, contact details for the study team are provided should the participant have any questions before, during, or after taking part. We have also provided a list of locally relevant support services at the end of each patient information sheet, which participant can consult.

Patient and public involvement (PPI)

The trail had dedicated PPI group and had considerable regular input from PPI group at every stage.

Dissemination

The results of the feasibility trial will be submitted for publication to a peer-reviewed journal and presented at relevant conferences. A separate manuscript on the qualitative aspect of the study will be written as well. This work is part of a PhD for the lead author (MS) who will present and submit data as a PhD Thesis to the University of Nottingham. The work will also be made available to study participants via the NDDC Biomedical Research Unit website.

Conclusions

At present there is no provision of testing to establish the severity of potential liver disease in specialist alcohol services. This randomised control trial aims to establish the feasibility of conducting a larger national trial to test the effectiveness of FibroScan informed advice and alcohol recovery video stories in changing high-risk drinking behaviour in community alcohol services common to UK practice.

For peer review only

References

1. Williams R, Aithal G, Alexander GJ, et al. Unacceptable failures: the final report of the Lancet Commission into liver disease in the UK. *Lancet.* 2020;395(10219):226-239.
2. World Helath Organisation (WHO). 2018 WHO global stats report on alcohol and health.pdf. 2018.
3. Williams R, Aspinall R, Bellis M, et al. Addressing liver disease in the UK: a blueprint for attaining excellence in health care and reducing premature mortality from lifestyle issues of excess consumption of alcohol, obesity, and viral hepatitis. *Lancet.* 2014;384(9958):1953-1997.
4. Osna NA, Donohue TM, Jr., Kharbanda KK. Alcoholic Liver Disease: Pathogenesis and Current Management. *Alcohol research : current reviews.* 2017;38(2):147-161.
5. British Liver Trust. 2019 British liver trust The-alarming-impact-of-liver-disease-FINAL-June-.pdf. 2019
6. Parker R. The natural history of alcohol-related liver disease. *Current Opinion in Gastroenterology.* 2020;36(3).
7. Public Health England (PHE). 2019 Statistics on Alcohol, England 2019 [PAS] - NHS Digital.pdf. 2019.
8. House of Commons Health Committee. Government's Alcohol Strategy. Third Report of Session.pdf. 2012.
9. Public Health England (PHE). Statistics on Alcohol, England 2020. 2020.
10. Julien J, Ayer T, Bethea ED, Tapper EB, Chhatwal J. Projected prevalence and mortality associated with alcohol-related liver disease in the USA, 2019–40: a modelling study. *The Lancet Public Health.* 2020;5(6):e316-e323.
11. O'Donnell A, Anderson P, Newbury-Birch D, et al. The impact of brief alcohol interventions in primary healthcare: a systematic review of reviews. *Alcohol and alcoholism (Oxford, Oxfordshire).* 2014;49(1):66-78.
12. Kaner EF, Beyer F, Dickinson HO, et al. Effectiveness of brief alcohol interventions in primary care populations. *The Cochrane database of systematic reviews.* 2007(2):Cd004148.
13. The National Institute fro Helath and Care Excellence (NICE). Alcohol-use disorders: prevention. 2010.
14. Roberts SE, John A, Brown J, Napier DJ, Lyons RA, Williams JG. Early and late mortality following unscheduled admissions for severe liver disease across England and Wales. *Aliment Pharmacol Ther.* 2019;49(10):1334-1345.
15. Harman DJ, Ryder SD, James MW, et al. Direct targeting of risk factors significantly increases the detection of liver cirrhosis in primary care: a cross-sectional diagnostic study utilising transient elastography. *BMJ Open.* 2015;5(4):e007516.
16. Harman DJ, Ryder SD, James MW, et al. Obesity and type 2 diabetes are important risk factors underlying previously undiagnosed cirrhosis in general practice: a cross-sectional study using transient elastography. *Aliment Pharmacol Ther.* 2018;47(4):504-515.
17. Subhani M, Knight H, Ryder S, Morling JR. Does Advice Based on Biomarkers of Liver Injury or Non-Invasive Tests of Liver Fibrosis Impact High-Risk Drinking Behaviour: A Systematic Review With Meta-analysis. *Alcohol and Alcoholism.* 2021.
18. Rennick-Egglestone S, Ramsay A, McGranahan R, et al. The impact of mental health recovery narratives on recipients experiencing mental health problems: Qualitative analysis and change model. *PloS one.* 2019;14(12):e0226201-e0226201.
19. White WL. Addiction recovery: its definition and conceptual boundaries. *Journal of substance abuse treatment.* 2007;33(3):229-241.
20. Tracy K, Wallace SP. Benefits of peer support groups in the treatment of addiction. *Subst Abuse Rehabil.* 2016;7:143-154.
21. Zimmerman GL, Olsen CG, Bosworth MF. A 'stages of change' approach to helping patients change behavior. *Am Fam Physician.* 2000;61(5):1409-1416.
22. Rennick-Egglestone S, Morgan K, Llewellyn-Beardsley J, et al. Mental Health Recovery Narratives and Their Impact on Recipients: Systematic Review and

For peer review only - http://bmjopen.bmj.com/site/about/guidelines.xhtml

Narrative Synthesis. *Canadian journal of psychiatry Revue canadienne de psychiatrie.* 2019;64(10):669-679.
23. Ng F, Charles A, Pollock K, et al. The mechanisms and processes of connection: developing a causal chain model capturing impacts of receiving recorded mental health recovery narratives. *BMC psychiatry.* 2019;19(1):413-413.
24. Rennick-Egglestone S, Elliott R, Smuk M, et al. Impact of receiving recorded mental health recovery narratives on quality of life in people experiencing psychosis, people experiencing other mental health problems and for informal carers: Narrative Experiences Online (NEON) study protocol for three randomised controlled trials. *Trials.* 2020;21(1):661.
25. Charles Teddlie FY. Mixed Methods Sampling A Typology With Examples. *Journal of Mixed Methods Research.* 2007;1(1).
26. STOTLAND NL, MATTSON MG, BERGESON S. The Recovery Concept: Clinician and Consumer Perspectives. *Journal of Psychiatric Practice®.* 2008;14:45-54.
27. Public Health England (PHE). The international evidence on the prevention of drug and alcohol use Summary and examples of implementation in England. https://assets.publishing.service.gov.uk/government/uploads/system/uploads/attachment_data/file/774743/Preventing_drug_and_alcohol_misuse__international_evidence_and_implementation_examples.pdf. Published 2015. Accessed.
28. CONSORT. Consort Statement
29. Braun V, Clarke V. Reflecting on reflexive thematic analysis. *Qualitative Research in Sport, Exercise and Health.* 2019;11(4):589-597.
30. Cohen J. A Coefficient of Agreement for Nominal Scales. *Educational and Psychological Measurement.* 1960;20(1):37-46.
31. Tong A, Sainsbury P, Craig J. Consolidated criteria for reporting qualitative research (COREQ): a 32-item checklist for interviews and focus groups. *International Journal for Quality in Health Care.* 2007;19(6):349-357.
32. Chalmers J, Wilkes E, Harris R, et al. The Development and Implementation of a Commissioned Pathway for the Identification and Stratification of Liver Disease in the Community. *Frontline Gastroenterol.* 2020;11(2):86-92.

For peer review only

Authors' contributions

Mohsan Subhani: Written initial draft of the protocol, implemented changes and drafted final version of protocol and manuscript.

Katy Jones: Reviewed protocol and manuscript, provided specialist input for qualitative aspects of the protocol and contributed to the final manuscript.

Kirsty Sprange: Reviewed protocol and manuscript and contributed to the final manuscript.

Stefan Rennick-Egglestone: Reviewed protocol and manuscript, provided specialist input for work-package-2 of protocol and contributed to final manuscript

Holly Knight: Reviewed protocol and manuscript, provided specialist input for work-package-1 of protocol and contributed to final manuscript

Joanne R Morling: Reviewed protocol and manuscript, provided specialist input for health economics of protocol and contributed to final manuscript

Doyo G Enki: Statistical support, reviewed final manuscript

Andrew Wragg: Patient and public involvement coordinator, reviewed final manuscript

Stephen D Ryder: Chief investigator and senior author.

Funding statement

This work was supported by "National Institute for Health Research (NIHR)" grant number [RfPB NIHR201146].

Data sharing statement

The anonymised data that will support the findings of this study will be available from the corresponding author, [MS], upon reasonable request.

Competing interests' statement

No competing interests from any author

For peer review only

Figure Legends

Figure 1: The KLIFAD trial timeline and flow chart (RCT-randomised control trial, GCP-good clinical practice, AUDIT-alcohol use disorder identification test, SADQ-severity of alcohol dependence questionnaire). The trial has three work packages (WP), WP3 is in randomised feasibility trial.

Figure 2: Flow diagram for; Work-package 1 to create fibroscan scripted feedback and Work-package 2 to create alcohol recovery videos stories

Figure 3: Flow diagram for work-package-3 the randomised control trial flow chart (RCT-randomised control trial, GCP-good clinical practice, AUDIT-alcohol use disorder identification test, SADQ-severity of alcohol dependence questionnaire). Work-package 3 is feasibility randomised control trial.

For peer review only

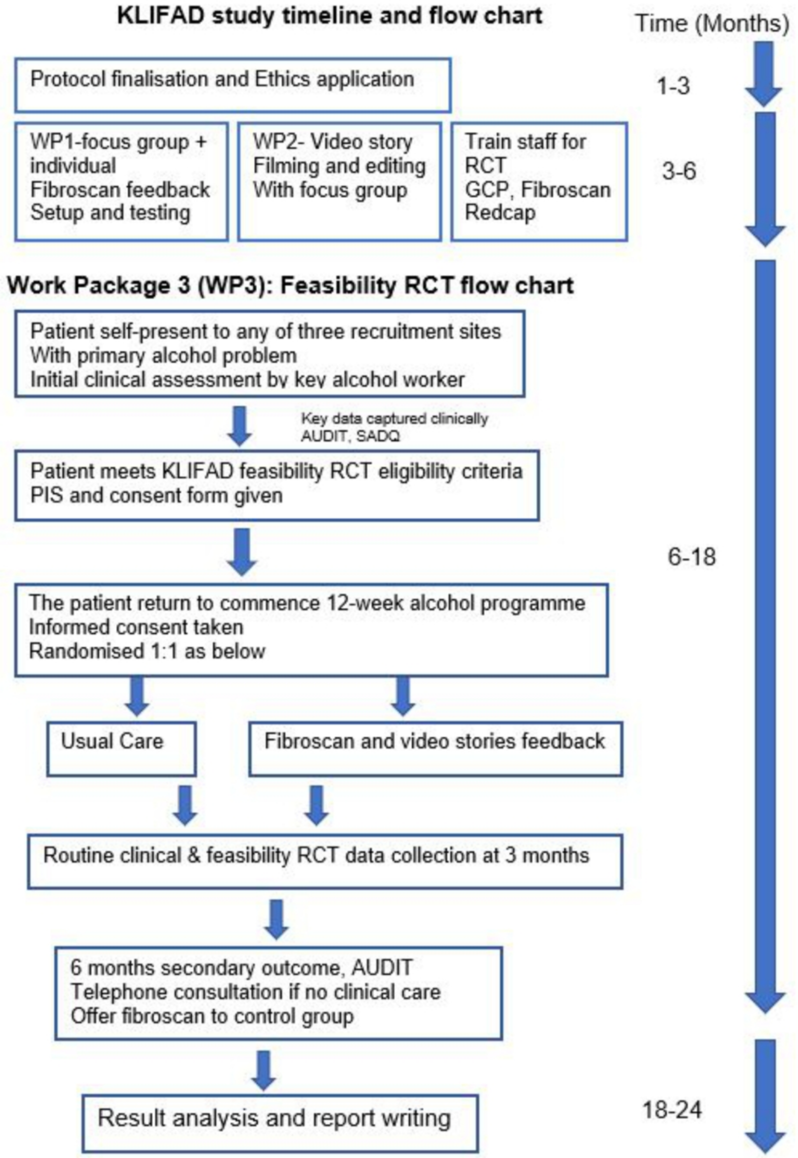

Figure 1: The KLIFAD trial timeline and flow chart (RCT-randomised control trial, GCP-good clinical practice, AUDIT-alcohol use disorder identification test, SADQ-severity of alcohol dependence questionnaire). The trial has three work packages (WP), WP3 is in randomised feasibility trial.

149x197mm (300 x 300 DPI)

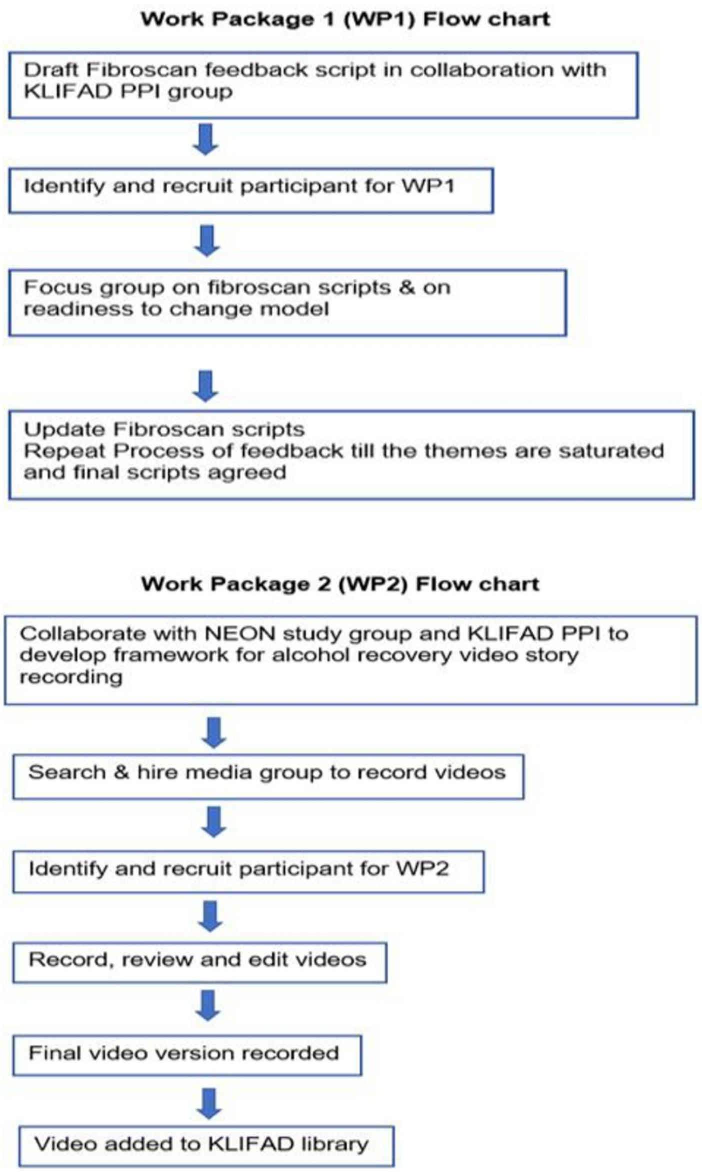

Figure 2: Flow diagram for; Work-package 1 to create fibroscan scripted feedback and Work-package 2 to create alcohol recovery videos stories

147x216mm (300 x 300 DPI)

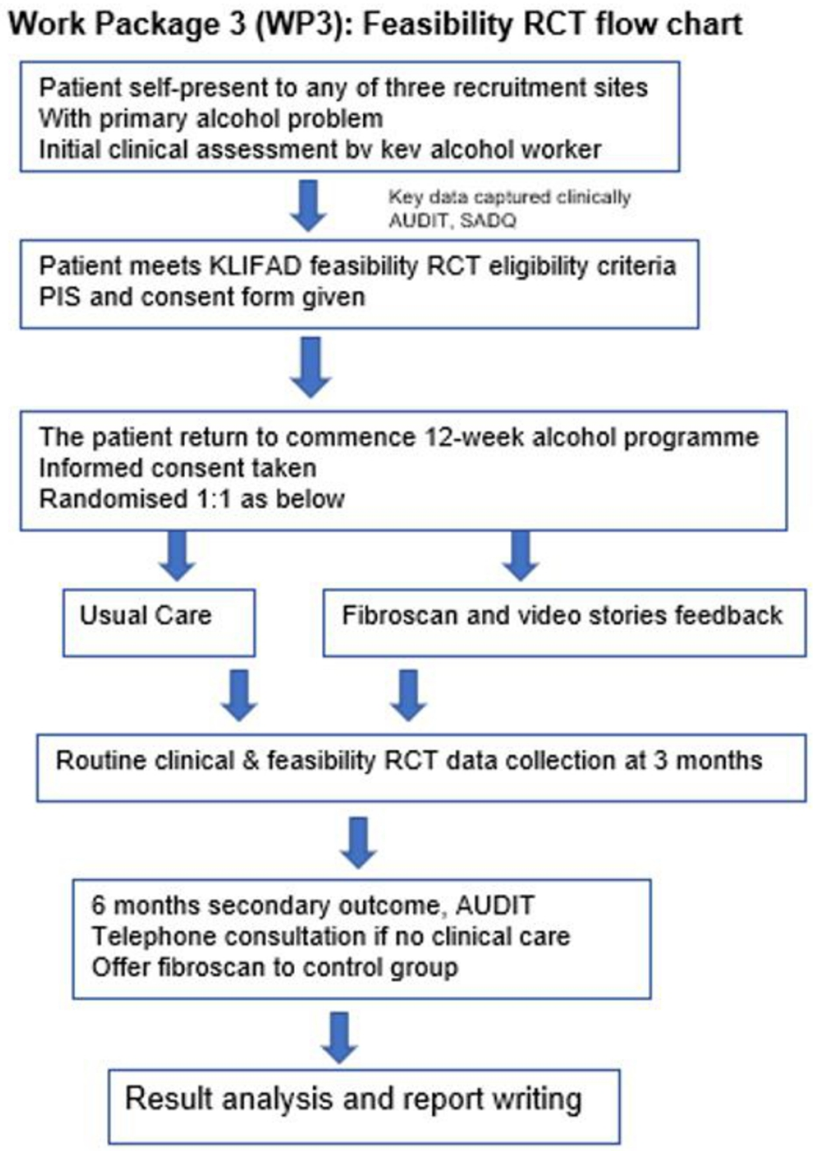

Figure 3: Flow diagram for work-package-3 the randomised control trial flow chart (RCT-randomised control trial, GCP-good clinical practice, AUDIT-alcohol use disorder identification test, SADQ-severity of alcohol dependence questionnaire). Work-package 3 is feasibility randomised control trial.

149x202mm (300 x 300 DPI)

Definitions

The following definitions are relevant to the KLIFAD trial:

Recovery Definition

For the KLIFAD trial we adopted the following definition of "Recovery"

"A period of sustained abstinence from alcohol creating a deeply personal, unique process of change, a way of living a satisfying, hopeful and contributing life even with limitations caused by illness. A process involving the development of new meaning or purpose in one's life which maximises health and wellbeing and participation in the rights, roles and responsibilities of society"[1-4].

Recovery story

A story told by a person about their journey of recovery.

In KLIFAD we are using recovery stories which are primarily first-person lived experience accounts, which include elements of both adversity/struggle and of strength/success/survival related to AUD, and which refer to events or actions over a period of time. Some stories will include brief fragments presenting clinical perspectives on a case, provided by a clinician who worked with the narrator[5].

Story narrator

The person telling their own recovery story.

Story recipient

The person viewing, reading or listening to someone else's recovery story.

KLIFAD Library

A collection of recovery stories intended for use in the KLIFAD feasibility trial.

Alcohol misuse

The National Institute on Alcohol Abuse and Alcoholism (NIAAA) define alcohol misuse as "alcohol consumption that puts individuals at increased risk for adverse health and social consequences"[6]

Alcohol use disorders

The NIAAA define AUD as "a chronic relapsing brain disorder characterized by an impaired ability to stop or control alcohol use despite adverse social, occupational, or health consequences" [6].

For peer review only - http://bmjopen.bmj.com/site/about/guidelines.xhtml

SP-Focus Group Guide WP1 V2.0

Focus group Guide participants

Work Package 1 (WP1)

Version 2.0 Date:14/10/2020

Study title: **Does knowledge of liver fibrosis affect high-risk drinking behaviour (KLIFAD)? A feasibility randomised controlled trial**

To begin

Welcome to the focus group session. Thanks for taking the time to join us to talk about liver disease screening.

You were invited here today because you attended a liver scan appointment and were given your level of risk for liver disease using a Fibroscan machine. We would like to understand how to provide the best experience for patients undergoing the scan. This includes how the person operating the Fibroscan machine discusses the scan itself and then delivers the results of the scan to patients. We will ask you to read through a script we have prepared to help operators talk through the scan and also a document that provides patients with their results.

Everyone's risk of liver disease may be different. Because everyone has very different life experiences, there are no wrong answers to these questions, but rather different points of view. Please feel free to share your point of view even if it differs from what others have said. Keep in mind that we're just as interested in negative comments as positive comments, and at times the negative comments are the most helpful.

Logistics

- Focus group will last about 2 hours
- Feel free to move around
- Where is the bathroom? Exit?
- Help yourself to refreshments

Ground Rules

- Hope that everyone feels comfortable enough to participate.
- Information provided in the focus group must be kept confidential
- Stay with the group and please don't have side conversations
- Turn off mobile phones if possible
- This is an opportunity to help contribute to the treatment of liver disease!

You've probably noticed the microphone. I'm tape recording the session because I don't want to miss any of your comments. People often say very helpful things in these discussions and I can't write fast enough to get them all down.

For peer review only - http://bmjopen.bmj.com/site/about/guidelines.xhtml

If you talk about anyone else during the focus group by name (such as a friend or member of staff) – then we will keep their name anonymous when we write up the results by providing them with a false name. Likewise (the participant) we will also keep your identity anonymous during the write-up by giving you a false name in any reports resulting from this study

Are you okay with this? Do you have any questions?

- Answer any questions they have
- If they do not want to participate, thank them for their time and escort them out of the venue. If they have participated via telephone or over video conferencing – finish the call.

Beginning the focus group

Start recording the interview on the Dictaphone.

Firstly, I want you to think back to your liver scan appointment.

1. Did you understand why you were undergoing a fibroscan and what the scan involved?
2. What was your experience of the scan? Was there anything about the way the operator conducted the scan or talked to you about the scan that you liked/disliked/found helpful?
3. After the scan, what information were you provided with? Including your results, any feedback from the scan operator, and any other information about liver disease?
    a. Was any of this difficult to understand? What information did you find most helpful?
4. Did the scan and/or scan results prompt you to make some changes to improve your liver health?
    a. If you received normal scan results, did you still want to make lifestyle changes?

Now I'd like us to spend the rest of the session today reviewing the documents in front of you. Please take some time to read through these documents and write any thoughts you have about the wording or how the information is presented on the document.

Provide participants with pens
Give participants approximately 10-15 minutes to read through script and fibroscan results

Let's review the operator script. Imagine you were receiving this information from a fibroscan operator.

1. Do you understand the information presented in the script?
    a. What did you like/dislike about the script? What information was helpful/unhelpful? Was anything unclear?
2. Was there any information you felt was missing or that you think would make a useful addition to the script?

For peer review only - http://bmjopen.bmj.com/site/about/guidelines.xhtml

a. Do you have any suggested changes or improvements to the script?

Now let's review the fibroscan result documents. There are three different results a patient can receive, depending on their liver stiffness. Imagine you were receiving this information from a fibroscan operator.

1. Do you think the results made sense for each level of liver disease stiffness?
    a. Did you understand the information? What information was helpful/unhelpful? Was anything unclear?
2. How did the documents make you feel?
    a. Did anyone have a negative reaction/positive reaction?
3. Did you like the way the results were presented (e.g. graphically, visually)?
    a. What would you change? Would you prefer the results to be presented as a value, on a scale, on a graph etc.?
4. Would you feel confident knowing what your result was and how to go about making lifestyle changes from this information?
    a. If not, why and what could we include that would help improve your confidence? Do you think the results documents would need explaining further by the operator?
5. Does anyone have additional thoughts about a specific result document (normal, likely fibrosis, likely cirrhosis)?
    a. Do you think the information reflects the level of risk and need for behaviour change?
6. Is there any other information we should include in the results document?
    a. Do you have any suggested changes or improvements to the results?

Close

Okay, that reaches the end of the questions I wanted to ask today. Is there anything else you wanted to add or talk about that we didn't talk about today?

If you're okay to end the focus group there, I'll switch the Dictaphone off, thank you!

Debriefing

- Thank you for speaking to us.
- Provide participants with a sheet which outlines the range of services etc, go through it with them. If there is any particular service/resource that they have expressed an interest in – then signpost them to it.
    - If they have participated via telephone– a state that they can be sent this via email if this wish or it can be read out to them.
- Thank them again, and ask if they are feeling okay to leave the building/ or hang up/exit the call.

For peer review only - http://bmjopen.bmj.com/site/about/guidelines.xhtml

SP-Change model questionnaire (CMQ) V1.0

Change model questionnaire

Work package 1 (WP1) V1.0 26/10/2020

Study title: Does knowledge of liver fibrosis affect high-risk drinking behaviour (KLIFAD)? A feasibility randomised controlled trial

Your doctor may have asked you to cut down how much alcohol you are drinking. Please find the statement that best describes the way you feel right now about cutting down your alcohol use to the amount the research team recommends

- ☐ I am continuing to drink at the same level and right now I am not considering reducing how much I drink
- ☐ I am continuing to drink at the same level but and right now I am considering reducing how much I drink
- ☐ I am continuing to drink at the same level but I am planning to reduce how much I drink
- ☐ Right now I have reduced how much alcohol I drink, and have maintained this for less than six months
- ☐ Right now I have reduced how much alcohol I drink, and have maintained this for more than six months

For peer review only

Qualitative interview Guide

Work package 3 (WP 3) Feasibility RCT

Study title: **Does knowledge of liver fibrosis affect high-risk drinking behaviour (KLIFAD)? A feasibility randomised controlled trial**

To begin

- Go over the study information again with the participant:
    - Thank you for coming to/agreeing to take part in the interview today…
    - Explain what will happen:
    - 'You'll be asked brief questions about your experience of taking part in the KLIFAD study and some questions about how you felt about taking part in this study and how it might have had an impact on you".
    - There are no 'right' or 'wrong' answers – I am not here to judge you, but to listen to your experiences as everyone's experience is valuable.
    - You can tell us as little or as much information as you want to during this interview, it is kept confidential in the research team. We may use a transcription service, but they are required to sign a confidentiality agreement and identifiers are removed from the typed-up transcript.
    - You can pause or stop the interview at any time if you want a break, you feel uncomfortable or don't want to continue with the interview.
    - After the interview, I will provide you with information about services and resources – that you may find useful if you have any concerns about what you have told us.
- Are you okay with all this? Do you have any questions?
    - Answer any questions they have
    - If they do not want to participate, thank them for their time and escort them out of the venue. If they have participated via telephone or over video conferencing – finish the call.

- Note: We will ask our PPI group about whether to include clarification of specific terms at this point. For example, relapse or lapse or teetotal/sober etc to ensure we ask questions in the participant's preferred way of talking about their alcohol use.
- If you talk about anyone else during the interview by name (such as a friend or member of staff) – then we will keep their name anonymous when we write up the results by providing them with a false name. Likewise (the participant) we will also keep your identity anonymous during the write-up by giving you a false name in any reports resulting from this study
- If you are satisfied with this, please confirm that you still consent to take part.
    - They will have already consented to take part when they signed up. Check you have received this consent (if was by e-mail or post).
    - If unsatisfied and does not want to take part – thank them for their time and guide them out of the venue/end the call.

For peer review only - http://bmjopen.bmj.com/site/about/guidelines.xhtml

Beginning the interview

Start recording the interview on the Dictaphone.

Here we can ask an introductory question to establish some rapport.

Your experience of the KLIFAD study

Q. Have you ever taken part in a research study before?

Q. Can you take me through what you remember about the KLIFAD study? (If they get into specifics of the results…. We'll touch on that later, for now, I'd like you to think about your experience of the scan process as a whole, for example how you felt about the scan or the staff who scanned you.)

Q. Overall, how do you feel about taking part in the KLIFAD study?

Follow up questions: If positive feedback: What did you particularly like?

If negative feedback: What did you not like/thought could be different?

Q. In regard to the fibroscan, did you understand why you were invited to have this scan? Did the staff give you enough information about the scan? Was there anything about the whole process you liked/didn't like?

Q. Where did you watch the stories? Did you watch it with anyone else? What was your response to them?

Your feelings about getting the KLIFAD study

Q. Can you tell me what you remember about your fibroscan scan result?

Follow-up questions: Can you remember the specific value, scale, what the value meant (potential liver disease etc)? Was the result explained clearly, did you understand it? Can you think of ways to improve how we give people their scan results? Is there anything else you think would be helpful to know when you receive your scan result?

For peer review only - http://bmjopen.bmj.com/site/about/guidelines.xhtml

For peer review only

Q. Do you remember how you felt when you first got your fibroscan result? Explore their thoughts and feelings here by using reflection 'So, I'm hearing that you felt confused and a bit frightened'. Also can use follow-up questions if appropriate e.g., Can you talk a bit more about why you felt scared? Can you describe your feeling of relief? Etc.

Q. What did it feel like watch stories describing other people's experiences of receiving a fibroscan? Follow up questions: Which stories can you remember accessing? Can you describe any ways in which these made an immediate impact on you? Can you describe any ways in which these have made a longer-tem impact on you? Did you learn anything from the stories?

Q. Did you discuss the KLIFAD study with anyone?

Follow up questions: What part did you talk about? (Scan/story/both?). Who did you talk to about it? How did they feel about it? If they didn't talk to anyone about it, ask why they didn't

Q. Now that a bit of time has passed, how do you feel about taking part in the KLIFAD study?

Your use of alcohol since you took part in the KLIFAD study

Q. Can you talk about your use of alcohol at a few different time points? It may be hard to remember this far back so sometimes it's helpful to look at a calendar and plot out some key dates (e.g. birthdays, trips away) that can help you remember.

1. Your use of alcohol (if any) just before you had your fibroscan result
2. Your use of alcohol (if any) on the day or days after you had your fibroscan result
3. Your use of alcohol (if any) two weeks after you had your result
4. Your use of alcohol (if any) over the last month

Q. Do you think the KLIFAD study changed your use of alcohol in any way?

If yes: explore, how, why do they think it affected it. If no: invite them to talk about that.

Explore if they sought out additional supports e.g. AA

Follow-up: Had you thought about changing before taking part in this study?

Q. If yes to changes, what were your main reasons for making these changes?

Q. If no, tell me more about why you didn't want to or didn't feel able to make changes at that time.

For peer review only - http://bmjopen.bmj.com/site/about/guidelines.xhtml

Follow-up questions: Was there anything that helped you make the changes? Was there anything that was a barrier to making changes?

Close

Okay that reaches the end of the questions I wanted to ask you. Is there anything else you wanted to add or talk about that we didn't talk about today?

If you're okay to end the interview there, I'll switch the Dictaphone off, thank you!

Debriefing

- Thank you for speaking to us.
- How are you feeling – is there anything in the interview has troubled you or upset you?
- Provide participant with sheet which outlines range of services etc, go through it with them. If there is any particular service/resource that they have expressed an interest in – then signpost them to it.
    - If they have participated via telephone– state that they can be sent this via email if this wish or it can be read out to them.
- Thank them again, and ask if they are feeling okay to leave the building/ or hang up/exit the call.

For peer review only

For peer review only - http://bmjopen.bmj.com/site/about/guidelines.xhtml

1. Anthony WA. Recovery from mental illness: The guiding vision of the mental health service system in the 1990s. *Psychosocial Rehabilitation Journal.* 1993;16(4):11-23.
2. Rennick-Egglestone S, Morgan K, Llewellyn-Beardsley J, et al. Mental Health Recovery Narratives and Their Impact on Recipients: Systematic Review and Narrative Synthesis. *Canadian journal of psychiatry Revue canadienne de psychiatrie.* 2019;64(10):669-679.
3. Witkiewitz K, Wilson AD, Pearson MR, et al. Profiles of recovery from alcohol use disorder at three years following treatment: can the definition of recovery be extended to include high functioning heavy drinkers? *Addiction (Abingdon, England).* 2019;114(1):69-80.
4. <Policy report - A vision of recovery_ UKDPC recovery consensus group.pdf>.
5. Llewellyn-Beardsley J, Rennick-Egglestone S, Callard F, et al. Characteristics of mental health recovery narratives: Systematic review and narrative synthesis. *PloS one.* 2019;14(3):e0214678.
6. Alcoholism NIoAAa. Helping patients who drink too much: a clinician’s guide, updated 2005 edition. Rockville: National Institutes of Health. 2005.

For peer review only

Participant Consent Form
Work Package 3 (WP 3) Feasibility RCT

Version 2.2 Date:24/12/2020

Does knowledge of liver fibrosis affect high-risk drinking behaviour (KLIFAD)? A feasibility randomized controlled trial

Chief Investigator: Professor Stephen Ryder

For peer review only

Please initial each box

1. I confirm that I have read and understood the participant information sheet dated ____________ (version _______) for the above study and have had the opportunity to ask questions. ☐
2. I understand that my participation is voluntary and that I am free to withdraw at any time without my medical care or legal rights being affected. ☐
3. I understand that my medical records may be looked at by authorised individuals from the Sponsor for the study and the UK Regulatory Authority in order to check that the study is being carried out correctly. ☐
4. I understand that should I withdraw from the above study, the data collected from me up to that point will be used in analysing the results of the trial. ☐
5. I consent to the storage, including electronic, of my personal information for this study. I understand that any information that could identify me will be kept strictly confidential and that no personal information will be included in the study report or other publication. ☐
6. I agree that my GP, or if required any other doctor treating me, will be notified of my participation in this study and of my fibroscan results if they shows advance fibrosis ☐
7. I understand I will be offered a voluntary video story recording at end of the study. ☐
8. I consent to access my medical and mental health record via NHS digital services as part of the study ☐
9. I agree to the storage of my anonymized data and that this may be kept for future research papers related to this study and for the completion of the study ☐
10. I agree to take part in the study ☐

11

Name of the participant *(Print)* date (dd/mm/yyyy) Participant
s signature

Name of person taking consent *(Print)* date (dd/mm/yyyy) Signature

For peer review only

Participant Consent Form
Qualitative Interview WP3

Version 2.2 Date:24/12/2020

Does knowledge of liver fibrosis affect high-risk drinking behaviour (KLIFAD) A feasibility randomized controlled trial

Chief Investigator: Professor Stephen Ryder

Please initial each box

10. I confirm that I have read and understood the PIS Qualitative interview WP 3dated ____________ (version _______) for the above study and have had the opportunity to ask questions. ☐

11. I understand that my participation is voluntary and that I am free to withdraw at any time without my medical care or legal rights being affected. In addition, should I not wish to answer any question, I am free to decline. ☐

12. I understand that my study and medical records may be looked at by authorised individuals from the Sponsor for the study and the UK Regulatory Authority in order to check that the study is being carried out correctly. ☐

13. I understand that should I withdraw from the above study, the data collected from me up to that point will be used in analysing the results of the trial. ☐

14. consent to the storage, including electronic, of personal information for this study. I understand that any information that could identify me will be kept strictly confidential and that no personal information will be included in the study report or other publication. ☐

15. understand that audio recordings will be used only for analysis and that extracts from the interview, from which I would not be personally identified, may be used in any conference presentation, report or journal article developed as a result of the research. I understand that no other use will be made of the recording without my written permission and that no one outside the research team will be allowed access to the original recording. ☐ ☐

16. understand a NHS approved professional transcription service can be used to transcribe the interveiew audio recoding. ☐

17. understand that if I tell the researcher anything that could cause me or someone else harm, the researcher may have to share this with the relevant healthcare professional. ☐

For peer review only

18. agree to take part in the interview that will be audio recorded (typed up recordings will be anonymised).

Name of the participant *(Print)* date (dd/mm/yyyy) Participant signature

Name of person taking consent *(Print)* date (dd/mm/yyyy) Signature

For peer review only

Participant Consent Form

Work package 1(WP 1) Focus group

Version 2.2 Date:22/12/2020

Does knowledge of liver fibrosis affect high-risk drinking behaviour? (KLIFAD): A feasibility randomized controlled trial

Chief Investigator: Professor Stephen Ryder

Please initial each box

1. I confirm that I have read and understood the participant information sheet Work package 1(WP 1) Focus group dated ____________ (version _______) for the above study and have had the opportunity to ask questions.
2. I understand that my participation is voluntary and that I am free to withdraw at any time and without my medical care or legal rights being affected. In addition, should I not wish to answer any question or questions, I am free to decline.
3. I understand that my study and medical records may be looked at by authorised individuals from the Sponsor for the study and the UK Regulatory Authority in order to check that the study is being carried out correctly.
4. I understand that should I withdraw from the above study, the data collected from me up to that point will be used in analysing the results of the trial.
5. I agree to participate in a Focus group, and I understand that the focus group will be audio recorded. I agree with the audio recording and understand that my responses will be kept strictly confidential.
6. I understand that audio recordings will be used only for analysis and that extracts from the interview, from which I would not be personally identified, may be used in any conference presentation, report or journal article developed as a result of the research. I understand that no other use will be made of the recording without my written permission and that no one outside the research team will be allowed access to the original recording.
7. I understand a NHS approved professional transcription service can be used to transcribe the the focus group audio recoding.
8. I agree to the storage of my anonymized data and that this may be kept for future research papers related to this study and for the completion of the study.

For peer review only - http://bmjopen.bmj.com/site/about/guidelines.xhtml

9. I understand that I will be offered the opportunity of making a voluntary video story recording

10. I agree to take part in the study

Name of the participant *(Print)* date (dd/mm/yyyy) Participant signature

Name of person taking consent *(Print)* date (dd/mm/yyyy) Signature

For peer review only

For peer review only - http://bmjopen.bmj.com/site/about/guidelines.xhtml

Participant Consent Form

Work package 1(WP 1) Key Alcohol Worker Focus group

Version 1.0 Date:22/12/2020

Does knowledge of liver fibrosis affect high-risk drinking behaviour? (KLIFAD): A feasibility randomized controlled trial

Chief Investigator: Professor Stephen Ryder

Please initial each box

11. I confirm that I have read and understood the participant information sheet Work package 1(WP 1) Key Alcohol Worker Focus group dated ____________ (version _______) for the above study and have had the opportunity to ask questions.

12. I understand that my participation is voluntary and that I am free to withdraw at any time and without my medical care, legal or employment rights being affected.

13. I understand that should I not wish to answer a question, and I am free to decline.

14. I understand that should I decide to withdraw from the above study, the data collected from me up to that point will be used in analyzing the results of the study

15. I agree to participate in a Focus group, and I understand that the focus group will be audio recorded. I agree with the audio recording and understand that my responses will be kept strictly confidential.

16. I understand that my name will not be linked with the research materials and will not be identified or identifiable in the report or reports that result from the research.

17. I understand that audio recordings will be used only for analysis and that extracts from the interview, from which I would not be personally identified, may be used in any conference presentation, report or journal article developed as a result of the research. I understand that no other use will be made of the recording without my written permission and that no one outside the research team will be allowed access to the original recording.

18. I agree to the storage of my anonymized data and that this may be kept for future research papers related to this study and for the completion of the study.

19. I agree to take part in the study

For peer review only - http://bmjopen.bmj.com/site/about/guidelines.xhtml

Name of the participant *(Print)* date (dd/mm/yyyy) Participant signature

Name of person taking consent *(Print)* date (dd/mm/yyyy) Signature

For peer review only

For peer review only - http://bmjopen.bmj.com/site/about/guidelines.xhtml

Participant Consent Form
Work package 2 (WP 2)Video recording

Version 2.2 Date:22/12/2020

Does knowledge of liver fibrosis affect high-risk drinking behaviour (KLIFAD)? A feasibility randomized controlled trial

Chief Investigator: Professor Stephen Ryder

Please initial each box

1. I confirm that I have read and understood the participant information sheet Work package 2 (WP 2) Video recording dated ____________ (version _______) for the above study and have had the opportunity to ask questions. ☐

2. I understand that my participation is voluntary and that I am free to withdraw at any time without my medical care or legal rights being affected. In addition, should I not wish to answer any particular question or questions, I am free to decline. ☐

3. I understand that my study and medical records may be looked at by authorised individuals from the Sponsor for the study and the UK Regulatory Authority in order to check that the study is being carried out correctly. ☐

4. I understand that should I withdraw from the above study, the data collected from me up to that point will be used in analysing the results of the trial . ☐

5. I understand that any information that could identify me will be kept strictly confidential Only anonymised information will be used for analysis for this study and may be used in any conference presentation, report or journal article developed as a result of the research from which I would not be personally identified, I understand that no other use will be made of the recording without my written permission. ☐

6. I understand that my identity cannot be hidden in the video recording and that there is a risk of my story becoming openly accessible to other people. ☐

7. I agree to the storage of my anonymized data and that this may be kept for future research papers related to this study and for the completion of the study ☐

8. I consent to participate in recovery video recording. ☐

9. I agree to take part in the study ☐

For peer review only - http://bmjopen.bmj.com/site/about/guidelines.xhtml

Name of participant *(Print)* date (dd/mm/yyyy) Participant signature

Name of person taking consent *(Print)* date (dd/mm/yyyy) Signature

For peer review only - http://bmjopen.bmj.com/site/about/guidelines.xhtml

Nottingham Recovery Network

Assessment Form

Clean Slate
Criminal Justice Substance Misuse Service

OFFICE USE ONLY

Date received:<br>Referred by:<br>If SELF, how did they hear about the service:		Client Id:<br>Assessed by:<br>Assessment location:<br>Date:
Specific risk / need identified:	Probation<br>Y / N	

CLIENT DETAILS

GDPR: There is a privacy notice in each room - please read it

DRUG ☐ If drug(s) please state type:
DRUG & ALCOHOL ☐
ALCOHOL ☐

**Title:** Mr / Mrs / Ms / Miss / other (please state)	**G.P Name:**
**First name:** **Surname:**	Address:
Prefers to be known as:	
**Gender:** Male ☐ Female ☐ Other ☐ Not specified ☐	
**Date of Birth:** **Age:**	Tel:
**Address:**	Currently receiving treatment? Y / N<br>Currently on prescribed medication? Y / N<br>Seen by GP in last month? Y / N<br>GP aware of substance misuse? Y / N
**Postcode:**	
**Home Tel:**	**Pharmacy current/preferred:**
**Mobile Tel:**	
**Email address:**	

Permission to be contacted: Home Visit ☐ Letter ☐ Telephone ☐ Text ☐ Email ☐

**Emergency Contact:**	**Telephone:**
**Medication:** Current ☐ Recent Past ☐ Past ☐ None ☐<br>Type & Dosage – List<br>...<br>...<br>Prescribed by:	**Known Allergies:** Y / N<br>List:<br>...<br>...<br>...
**Barriers to Accessing Treatment? (include any disabilities)**	**Preferred Language:**<br>**Is an interpreter required** Y / N

For peer review only - http://bmjopen.bmj.com/site/about/guidelines.xhtml

Client initials: DoB:

DEMOGRAPHICS

Sexuality: ☐ Heterosexual ☐ Gay/Lesbian ☐ Bi-sexual ☐ Other ☐ Not stated

Marital Status: ☐ Single ☐ Cohabiting ☐ Married ☐ Separated ☐ Divorced ☐ Widowed

Religion: ☐ None ☐ Christian ☐ Buddhist ☐ Hindu ☐ Jewish ☐ Muslim ☐ Sikh ☐ Other ☐ Not stated

Nationality: ☐ UK ☐ Other (please state) ....

Ethnic category

White	Mixed	Asian/Asian British	Black/Black British	Other Ethnic
British ☐	White & Black Caribbean ☐	Indian ☐	Caribbean ☐	Chinese ☐
Irish ☐	White & Black African ☐	Pakistani ☐	African ☐	Any Other ☐
Other White ☐	White & Asian ☐	Bangladeshi ☐	Black British ☐	Not Known ☐
	Other Mixed ☐	Other Asian ☐	Other Black ☐	Not Stated ☐

Accommodation:
☐ Homeless – urgent housing problems
☐ Housing problems –i.e. staying with friends
☐ Owner occupier
☐ Tenant - Landlord (please specify) ....

Employment Status:
☐ Employed (full/part time)
☐ Long term sick or disabled
☐ Retired from paid work
☐ Unemployed and seeking work
☐ Other (please specify)..
☐ Homemaker
☐ Not receiving benefits
☐ Student
☐ Unpaid voluntary work

Time since last employed: ☐ Never employed ☐ Currently employed ☐ Less than 1 year ☐ 1 – 2 years ☐ 2 – 3 years ☐ >3 years (please state) ....

Sex Worker: Yes ☐ No ☐ **Current / Previous**
If **YES** working from premises or street ....

Ex Service Personnel: Yes ☐ No ☐ **Referral to Royal British Legion wanted?** Yes ☐ No ☐

Disability: Yes ☐ No ☐ Type ....

Carer: Yes ☐ No ☐ Support needs..

Debt Issues: Yes ☐ No ☐ Support wanted ....

Support services already engaged with:

Support wanted:

Treatment Goal:

For peer review only - http://bmjopen.bmj.com/site/about/guidelines.xhtml

Client initials: DoB:

PRESENTATION ON ASSESSMENT

(Intoxicated / withdrawing / appearance etc.)

SUBSTANCE MISUSE PAST 7 DAYS / 4 WEEKS

For peer review only

For peer review only - http://bmjopen.bmj.com/site/about/guidelines.xhtml

Client initials: DoB:

SUBSTANCE MISUSE HISTORY

(Why, when, who with, route, frequency, finances, motivations, withdrawal symptoms, harm minimisation)

For peer review only

Client initials: DoB:

TREATMENT HISTORY

Currently in drug or alcohol treatment Yes ☐ No ☐

Name of all agencies/services currently in contact with client and keyworkers' names (where applicable):

Previously sought help with substance use Yes ☐ No ☐

Previously received structured drug or alcohol treatment Yes ☐ No ☐

Date of first treatment episode:

Further details

PROBLEMATIC SUBSTANCE USE

Substance		Frequency (in last 28 days)	Amount/Units /Cost	Route	Age of 1st use
**Primary**					
**2nd**					
**3rd**					

Drug Screening Questionnaire (DAST)

Score ....

INJECTING STATUS

Currently injecting ☐ Previously ☐ Never injected ☐ Declined to answer ☐

ALCOHOL

Drinking Days .... /28 Units/day .... Units/week...

AUDIT .... SADQ...................................... Breath Alcohol: ....Mg/l BrAC

BBV

HIV Status: Negative ☐ Positive ☐ Not Known ☐ **Latest Test Date** ....

Hep C – Intervention Status

Offered and accepted ☐

Offered and refused ☐

Not appropriate to offer ☐

Hep C Tested Yes ☐ No ☐ **Latest Test Date** ....

Hep C Positive Yes ☐ No ☐ Not Known ☐

Hep B - Intervention Status

Offered and accepted ☐

Offered and refused ☐

Immunised already ☐

Not appropriate to offer ☐

Vaccination Count:

1 Vaccination ☐

2 Vaccinations ☐

3 Vaccinations ☐

Course Complete ☐

Referred For Hepatology Yes ☐ No ☐

For peer review only - http://bmjopen.bmj.com/site/about/guidelines.xhtml

PHYSICAL HEALTH

Does the client consider themselves to have a disability? Yes ☐ No ☐
Nature of disability

Does the client have any health problems? Yes ☐ No ☐
(allergies, asthma, epilepsy, diabetes, dental, women specific, sexual health, cardiac, respiratory, DVT, sleep, diet)

Smoker: No ☐ Yes ☐ Qty .... p/day

Does client have smoke alarm: Yes ☐ No ☐ **Referral made to Framework service**: Yes / declined

IMMEDIATE RISK IDENTIFIED: Physical Health & Overdose

Prompts: Regular injector / Injecting in high-risk areas / Poly substance use / Poly substance use – opiates and alcohol / Substance use related seizures or DTs? / Injects alone / Witnessed overdose by others / History of past overdose

Level of risk: No risk ☐ Low ☐ Medium ☐ High ☐

Likelihood of occurrence No risk ☐ Low ☐ Medium ☐ High ☐

ACTION(S):

For peer review only

Client initials: DoB:

PSYCHOLOGICAL HEALTH

Any current/historical contact with mental health services: Yes ☐ No ☐
(mental health diagnosis or symptoms, negative thoughts, self esteem, current mood, history of suicidal thoughts/acts, self-harm)

Mental Health treatment need Yes ☐ No ☐

Current services involved ....

Ever experienced overdose? Yes ☐ No ☐ Accidental ☐ Deliberate ☐

Date & Drugs involved

Treatment received

Has Naloxone been offered (please circle) **Accepted / Refused**

IMMEDIATE RISK IDENTIFIED: Individual

Prompts: Suicidal / Self-harm / Mental health / Domestic violence
Trigger: Where DV is identified complete Multi Agency Domestic Violence Risk Identification Form when appropriate

Level of risk: No risk ☐ Low ☐ Medium ☐ High ☐

Likelihood of occurrence No risk ☐ Low ☐ Medium ☐ High ☐

ACTION(S):

IMMEDIATE RISK IDENTIFIED: Personal Safety / Self Neglect

Prompts: Reliant on others / Difficulty in coping with everyday domestic tasks / Homeless/vulnerably housed / Recent threat (s) from others /Financial Vulnerability

Level of risk: No risk ☐ Low ☐ Medium ☐ High ☐

Likelihood of occurrence No risk ☐ Low ☐ Medium ☐ High ☐

DETAILS:

ACTION(S):

For peer review only - http://bmjopen.bmj.com/site/about/guidelines.xhtml

Client initials: DoB:

PARENTAL STATUS

Do you have any contact with children under 18yrs?: Yes ☐ No ☐

Children/Partner's Children: Yes ☐ No ☐ **Sole Carer:** Yes ☐ No ☐ **Other** ....

Do all/some of the children live with you?: All of the time ☐ Some of the time ☐ No ☐

No. of children: **Ages:**

Are any of the client's children (biological, step, foster, adoptive, guardian) or any of the children receiving early help or are they in contact with Children's Social Care?:

Child in need ☐ Early help ☐ Has a child protection plan ☐ Looked after child ☐ None ☐

Social Care Services Involved: Current ☐ Recent past ☐ Past ☐ None ☐

Further details

Is client or partner pregnant: Yes ☐ No ☐ **Due Date:**

Referred to Specialist Midwife Yes ☐ No ☐ Previous ☐

IMMEDIATE RISK IDENTIFIED: Child Care

Prompts: Currently pregnant / Responsible for any child(ren) / Intoxicated while solely responsible for child(ren)
Trigger: Also complete "Childcare & Family support Form" in line with guidance notes if required

Level of risk: No risk ☐ Low ☐ Medium ☐ High ☐

Likelihood of occurrence No risk ☐ Low ☐ Medium ☐ High ☐

ACTION(S):

Safe storage box issued Yes ☐ No ☐ Refused ☐

Childcare & Family Support Form completed Yes ☐ No ☐

Family/Carer Support Offered: Yes ☐ No ☐ Type...

For peer review only - http://bmjopen.bmj.com/site/about/guidelines.xhtml

FAMILY & RELATIONSHIPS

(family health and significant relationships, child protection, care issues, vulnerable adults, support networks)

DOMESTIC VIOLENCE

Have you ever been a victim or perpetrator of domestic violence?

Survivor ☐
Perpetrator ☐
Declined to answer ☐

Current ☐
Recent past ☐
Past ☐
None ☐

IMMEDIATE RISK IDENTIFIED: Domestic Violence

Where DV is identified complete Multi Agency Domestic Violence Risk Identification Form when appropriate

Level of risk:	No risk ☐	Low ☐	Medium ☐	High ☐
Likelihood of occurrence	No risk ☐	Low ☐	Medium ☐	High ☐

ACTION(S):

Black DV card issued ☐ **DASH form completed** ☐ **MARAC referral made** ☐

For peer review only - http://bmjopen.bmj.com/site/about/guidelines.xhtml

CRIMINAL JUSTICE/OFFENDING HISTORY

Current Criminal Justice Status (tick all that apply)

Community Order	☐	DRR	☐	RAR Days	☐
Licence	☐	ATR	☐	Suspended Sentence	☐
Sex Offender Registration	☐	Prolific Offender	☐	ROB	☐
MAPPA	☐	Paying Fines	☐	Schedule 1 Offender	☐

Further details of current criminal justice status:
(length of orders, name of workers involved)

Details of past criminal justice / offending history
(offences committed, length of sentences, targets of violence)

Any record of violent offences?
Further details:

For peer review only

IMMEDIATE RISK IDENTIFIED: Harm to Others

Prompts: Violence to others / Aggression to others / ever used a weapon in assault? / Ever made any preparations to commit harm to others? / Criminal record / Violence/ Domestic Violence/ Aggression towards professionals

Trigger: Where DV is identified complete Multi Agency Domestic Violence Risk Identification Form when appropriate

Level of risk:	No risk ☐	Low ☐	Medium ☐	High ☐
Likelihood of occurrence	No risk ☐	Low ☐	Medium ☐	High ☐

ACTION(S):

For peer review only - http://bmjopen.bmj.com/site/about/guidelines.xhtml

EXISTING RECOVERY CAPITAL

(Including information around housing, financial, education, employment, cultural issues, offending as well as drug & alcohol use)

For peer review only

DVLA / Occupational Concerns / Other

For peer review only - http://bmjopen.bmj.com/site/about/guidelines.xhtml

PERCEPTION OF ONGOING NEEDS & ACTIONS

Signposted / referred

☐ Debt advice ☐ CGL Jigsaw (ExFam) ☐ GP

☐ Health Shop ☐ Housing Aid ☐ Housing Crisis

☐ Smoking cessation ☐ Street Outreach ☐ Wellness in Mind

☐ Other (please state) ....

Next appointment date and time: Location: Worker:

Keyworker signature: Date:

Print Name:

DVLA Guidelines for people receiving treatment for Alcohol and/or Drug related difficulties.

You have stated that you hold a current driving licence and continue to drive. As such you have been issued with guidance relating to Alcohol &/or Drug and driving as identified above. Please sign to acknowledge your responsibility to act according to this guidance.

Signed: Date:

Print Name:

For peer review only - http://bmjopen.bmj.com/site/about/guidelines.xhtml

For peer review only

Reporting checklist for protocol of a clinical trial.

Based on the SPIRIT guidelines.

Instructions to authors

Complete this checklist by entering the page numbers from your manuscript where readers will find each of the items listed below.

Your article may not currently address all the items on the checklist. Please modify your text to include the missing information. If you are certain that an item does not apply, please write "n/a" and provide a short explanation.

Upload your completed checklist as an extra file when you submit to a journal.

In your methods section, say that you used the SPIRITreporting guidelines, and cite them as:

Chan A-W, Tetzlaff JM, Gøtzsche PC, Altman DG, Mann H, Berlin J, Dickersin K, Hróbjartsson A, Schulz KF, Parulekar WR, Krleža-Jerić K, Laupacis A, Moher D. SPIRIT 2013 Explanation and Elaboration: Guidance for protocols of clinical trials. BMJ. 2013;346:e7586

For peer review only

		Reporting Item	Page Number
**Administrative information**			
Title	#1	Descriptive title identifying the study design, population, interventions, and, if applicable, trial acronym	1
Trial registration	#2a	Trial identifier and registry name. If not yet registered, name of intended registry	1
Trial registration: data set	#2b	All items from the World Health Organization Trial Registration Data Set	1
Protocol version	#3	Date and version identifier	1
Funding	#4	Sources and types of financial, material, and other support	1
Roles and responsibilities: contributorship	#5a	Names, affiliations, and roles of protocol contributors	1, 16

Roles and responsibilities: sponsor contact information	#5b	Name and contact information for the trial sponsor	1
Roles and responsibilities: sponsor and funder	#5c	Role of study sponsor and funders, if any, in study design; collection, management, analysis, and interpretation of data; writing of the report; and the decision to submit the report for publication, including whether they will have ultimate authority over any of these activities	11.12
Roles and responsibilities: committees	#5d	Composition, roles, and responsibilities of the coordinating centre, steering committee, endpoint adjudication committee, data management team, and other individuals or groups overseeing the trial, if applicable (see Item 21a for data monitoring committee)	11,12
**Introduction**			
Background and rationale	#6a	Description of research question and justification for undertaking the trial, including summary of relevant studies (published and unpublished) examining benefits and harms for each intervention	5
Background and rationale: choice of comparators	#6b	Explanation for choice of comparators	5,6
Objectives	#7	Specific objectives or hypotheses	6
Trial design	#8	Description of trial design including type of trial (eg, parallel group, crossover, factorial, single group), allocation ratio, and framework (eg, superiority, equivalence, non-inferiority, exploratory)	6
**Methods: Participants, interventions, and outcomes**			
Study setting	#9	Description of study settings (eg, community clinic, academic hospital) and list of countries where data will be collected. Reference to where list of study sites can be obtained	6
Eligibility criteria	#10	Inclusion and exclusion criteria for participants. If applicable, eligibility criteria for study centres and individuals who will	Table 1

For peer review only

		perform the interventions (eg, surgeons, psychotherapists)	
Interventions: description	#11a	Interventions for each group with sufficient detail to allow replication, including how and when they will be administered	6,7,8
Interventions: modifications	#11b	Criteria for discontinuing or modifying allocated interventions for a given trial participant (eg, drug dose change in response to harms, participant request, or improving / worsening disease)	N/A
Interventions: adherance	#11c	Strategies to improve adherence to intervention protocols, and any procedures for monitoring adherence (eg, drug tablet return; laboratory tests)	11,12
Interventions: concomitant care	#11d	Relevant concomitant care and interventions that are permitted or prohibited during the trial	N/A
Outcomes	#12	Primary, secondary, and other outcomes, including the specific measurement variable (eg, systolic blood pressure), analysis metric (eg, change from baseline, final value, time to event), method of aggregation (eg, median, proportion), and time point for each outcome. Explanation of the clinical relevance of chosen efficacy and harm outcomes is strongly recommended	10, 11
Participant timeline	#13	Time schedule of enrolment, interventions (including any run-ins and washouts), assessments, and visits for participants. A schematic diagram is highly recommended (see Figure)	9
Sample size	#14	Estimated number of participants needed to achieve study objectives and how it was determined, including clinical and statistical assumptions supporting any sample size calculations	9
Recruitment	#15	Strategies for achieving adequate participant enrolment to reach target sample size	6,7,8,9
**Methods: Assignment of interventions (for controlled trials)**			
Allocation: sequence generation	#16a	Method of generating the allocation sequence (eg, computer-generated random numbers), and list of any factors for stratification. To reduce predictability of a random sequence, details of any planned restriction (eg, blocking) should be provided in a separate document that is unavailable to those who enrol participants or assign interventions	9

For peer review only

Allocation concealment mechanism	#16b	Mechanism of implementing the allocation sequence (eg, central telephone; sequentially numbered, opaque, sealed envelopes), describing any steps to conceal the sequence until interventions are assigned	9
Allocation: implementation	#16c	Who will generate the allocation sequence, who will enrol participants, and who will assign participants to interventions	9
Blinding (masking)	#17a	Who will be blinded after assignment to interventions (eg, trial participants, care providers, outcome assessors, data analysts), and how	N/A
Blinding (masking): emergency unblinding	#17b	If blinded, circumstances under which unblinding is permissible, and procedure for revealing a participant's allocated intervention during the trial	N/A
**Methods: Data collection, management, and analysis**			
Data collection plan	#18a	Plans for assessment and collection of outcome, baseline, and other trial data, including any related processes to promote data quality (eg, duplicate measurements, training of assessors) and a description of study instruments (eg, questionnaires, laboratory tests) along with their reliability and validity, if known. Reference to where data collection forms can be found, if not in the protocol	9
Data collection plan: retention	#18b	Plans to promote participant retention and complete follow-up, including list of any outcome data to be collected for participants who discontinue or deviate from intervention protocols	9,10,11
Data management	#19	Plans for data entry, coding, security, and storage, including any related processes to promote data quality (eg, double data entry; range checks for data values). Reference to where details of data management procedures can be found, if not in the protocol	11,12,13
Statistics: outcomes	#20a	Statistical methods for analysing primary and secondary outcomes. Reference to where other details of the statistical analysis plan can be found, if not in the protocol	11
Statistics: additional analyses	#20b	Methods for any additional analyses (eg, subgroup and adjusted analyses)	11

For peer review only

Statistics: analysis population and missing data	#20c	Definition of analysis population relating to protocol non-adherence (eg, as randomised analysis), and any statistical methods to handle missing data (eg, multiple imputation)	11
**Methods: Monitoring**			
Data monitoring: formal committee	#21a	Composition of data monitoring committee (DMC); summary of its role and reporting structure; statement of whether it is independent from the sponsor and competing interests; and reference to where further details about its charter can be found, if not in the protocol. Alternatively, an explanation of why a DMC is not needed	12,13
Data monitoring: interim analysis	#21b	Description of any interim analyses and stopping guidelines, including who will have access to these interim results and make the final decision to terminate the trial	12,13
Harms	#22	Plans for collecting, assessing, reporting, and managing solicited and spontaneously reported adverse events and other unintended effects of trial interventions or trial conduct	12
Auditing	#23	Frequency and procedures for auditing trial conduct, if any, and whether the process will be independent from investigators and the sponsor	11,12,13
**Ethics and dissemination**			
Research ethics approval	#24	Plans for seeking research ethics committee / institutional review board (REC / IRB) approval	1
Protocol amendments	#25	Plans for communicating important protocol modifications (eg, changes to eligibility criteria, outcomes, analyses) to relevant parties (eg, investigators, REC / IRBs, trial participants, trial registries, journals, regulators)	11,12,13
Consent or assent	#26a	Who will obtain informed consent or assent from potential trial participants or authorised surrogates, and how (see Item 32)	11,12,13
Consent or assent: ancillary studies	#26b	Additional consent provisions for collection and use of participant data and biological specimens in ancillary studies, if applicable	N/A
Confidentiality	#27	How personal information about potential and enrolled participants will be collected, shared, and maintained in order to protect	11,12,13

For peer review only

		confidentiality before, during, and after the trial	
Declaration of interests	#28	Financial and other competing interests for principal investigators for the overall trial and each study site	16
Data access	#29	Statement of who will have access to the final trial dataset, and disclosure of contractual agreements that limit such access for investigators	16
Ancillary and post trial care	#30	Provisions, if any, for ancillary and post-trial care, and for compensation to those who suffer harm from trial participation	13
Dissemination policy: trial results	#31a	Plans for investigators and sponsor to communicate trial results to participants, healthcare professionals, the public, and other relevant groups (eg, via publication, reporting in results databases, or other data sharing arrangements), including any publication restrictions	13
Dissemination policy: authorship	#31b	Authorship eligibility guidelines and any intended use of professional writers	13
Dissemination policy: reproducible research	#31c	Plans, if any, for granting public access to the full protocol, participant-level dataset, and statistical code	13
**Appendices**			
Informed consent materials	#32	Model consent form and other related documentation given to participants and authorised surrogates	11-20
Biological specimens	#33	Plans for collection, laboratory evaluation, and storage of biological specimens for genetic or molecular analysis in the current trial and for future use in ancillary studies, if applicable	N/A

The SPIRIT Explanation and Elaboration paper is distributed under the terms of the Creative Commons Attribution License CC-BY-NC. This checklist was completed on 28. June 2021 using https://www.goodreports.org/, a tool made by the EQUATOR Network in collaboration with Penelope.ai

For peer review only

BMJ Open

Does knowledge of liver fibrosis affect high-risk drinking behaviour (KLIFAD)?: Protocol for a feasibility randomised controlled trial

Journal:	*BMJ Open*
Manuscript ID	bmjopen-2021-054954.R1
Article Type:	Protocol
Date Submitted by the Author:	23-Sep-2021
Complete List of Authors:	Subhani, Mohsan; University of Nottingham, Nottingham Digestive Diseases Biomedical Research Centre (NDDC); Nottingham University Hospitals NHS Trust, NIHR Nottingham Biomedical Research Centre<br>Jones, Katy; University of Nottingham School of Medicine, Division of Psychiatry & Applied Psychology<br>Sprange, Kirsty; University of Nottingham, Nottingham Clinical Trials Research Unit<br>Rennick-Egglestone, Stefan; University of Nottingham School of Health Sciences,<br>Knight, Holly; University of Nottingham,<br>Morling, Joanne; University of Nottingham<br>Enki, Doyo; University of Nottingham, Medical Statistics<br>Ryder, Stephen; Nottingham University Hospitals NHS Trust, Nottingham Digestive Diseases Biomedical Research Centre (NDDC), School of Medicine
**Primary Subject Heading**:	Gastroenterology and hepatology
Secondary Subject Heading:	Addiction, Evidence based practice, Health economics, Public health
Keywords:	Hepatology < INTERNAL MEDICINE, Gastroenterology < INTERNAL MEDICINE, PUBLIC HEALTH

SCHOLARONE™
Manuscripts

I, the Submitting Author has the right to grant and does grant on behalf of all authors of the Work (as defined in the below author licence), an exclusive licence and/or a non-exclusive licence for contributions from authors who are: i) UK Crown employees; ii) where BMJ has agreed a CC-BY licence shall apply, and/or iii) in accordance with the terms applicable for US Federal Government officers or employees acting as part of their official duties; on a worldwide, perpetual, irrevocable, royalty-free basis to BMJ Publishing Group Ltd ("BMJ") its licensees and where the relevant Journal is co-owned by BMJ to the co-owners of the Journal, to publish the Work in this journal and any other BMJ products and to exploit all rights, as set out in our licence.

The Submitting Author accepts and understands that any supply made under these terms is made by BMJ to the Submitting Author unless you are acting as an employee on behalf of your employer or a postgraduate student of an affiliated institution which is paying any applicable article publishing charge ("APC") for Open Access articles. Where the Submitting Author wishes to make the Work available on an Open Access basis (and intends to pay the relevant APC), the terms of reuse of such Open Access shall be governed by a Creative Commons licence – details of these licences and which Creative Commons licence will apply to this Work are set out in our licence referred to above.

Other than as permitted in any relevant BMJ Author's Self Archiving Policies, I confirm this Work has not been accepted for publication elsewhere, is not being considered for publication elsewhere and does not duplicate material already published. I confirm all authors consent to publication of this Work and authorise the granting of this licence.

For peer review only

Does knowledge of liver fibrosis affect high-risk drinking behaviour (KLIFAD)?: Protocol for a feasibility randomised controlled trial

Mohsan Subhani[1,2], Katy A Jones[4], Kirsty Sprange[5], Stefan Rennick-Egglestone[6], Holly Knight[3], Joanne R Morling[1,2,3], Doyo G Enki[7], Andrew Wragg[2], Stephen D Ryder[1,2]

Affiliations

[1]Nottingham Digestive Diseases Biomedical Research Centre (NDDC), School of Medicine, University of Nottingham
[2]NIHR Nottingham Biomedical Research Centre, Nottingham University Hospitals NHS Trust and the University of Nottingham, Nottingham, UK
[3]Division of Epidemiology and Public Health, University of Nottingham, Nottingham, UK
[4]Academic Unit of Mental Health and Clinical Neuroscience, School of Medicine, C24 Institute of Mental Health, University of Nottingham
[5]Nottingham Clinical Trials Unit, University of Nottingham
[6]School of Health Sciences, Institute of Mental Health, University of Nottingham
[7]School of Medicine, University of Nottingham

Acronym

KLIFAD

Corresponding Author

Mohsan Subhani
Email: mohsan.subhani@nottingham.ac.uk Telephone: 0044 7572612429

Research reference numbers

IRAS Number:	273765
SPONSOR Number:	124481/2020
FUNDER Number:	RfPB NIHR201146
ISRCTN	16922410
Protocol version	V1.4

Sponsor

Nottinghamshire Healthcare NHS Foundation Trust
research@nottshc.nhs.uk

Funder

National Institute for Health Research
Scheme: Research for Patient Benefit (RfPB)

For peer review only

Abbreviations

ARLD	Alcohol-related liver disease
ARVS	Alcohol recovery video stories
AUD	Alcohol use disorders
AUDIT	Alcohol Use Disorder Identification Test
BRC	Biomedical Research Centre
CONSORT	Consolidated Standards of Reporting Trials
COREQ	Consolidated Criteria for Reporting Qualitative Studies
GCP	Good Clinical Practice
Kpa	Kilopascal
NDTMS	National Drug Treatment Monitoring System
NDDC	Nottingham Digestive Diseases Centre
NHS	National Health Service
NICE	National Institute of Clinical Excellence
NRN	Nottingham Recovery Network
NUH	Nottingham University Hospital
PIS	Patient information sheet
PPI	Patient and public involvement
RCT	Randomised Controlled Trial
REC	Research Ethics Committee
SADQ	Severity of Alcohol Dependence Questionnaire
UK	United Kingdom
WoSRES	West of Scotland Research Ethics Service
WP	Work package

For peer review only

For peer review only - http://bmjopen.bmj.com/site/about/guidelines.xhtml

Abstract

Introduction

Heavy drinkers in contact with alcohol services do not routinely have access to testing to establish the severity of potential liver disease. Transient elastography by FibroScan can provide this information. A recent systematic review suggested providing feedback to patients based on markers of liver injury can be an effective way to reduce harmful alcohol intake. This randomised control trial aims to establish the feasibility of conducting a larger national trial to test the effectiveness of FibroScan advice and alcohol recovery video stories in changing high-risk drinking behaviour in community alcohol services common to United Kingdom practice.

Methods and analysis

Feasibility trial consists of three work packages (WP). **WP1:** To draft a standardised script for FibroScan operators to deliver liver disease-specific advice to eligible participants having FibroScan. **WP2:** To create a video library of alcohol recovery video stories for use in the feasibility RCT (WP3). **WP3:** To test the feasibility of the trial design, including the FibroScan script and video stories developed in WP1 and WP2 in a one-to-one individual randomised trial in community alcohol services. Semi-structured interviews will be conducted at six months follow up for qualitative evaluation. Outcomes will be measures of the feasibility of conducting a later larger RCT related to participant recruitment and follow-up, intervention delivery, including the use of the KLIFAD FibroScan scripts and videos, clinical outcomes and the acceptability and experience of the intervention and trial-related procedures. Data analysis will primarily be descriptive to address the feasibility aims of the trial. All proposed analyses will be documented in a Statistical Analysis Plan.

Ethics and dissemination

The trial received favourable ethical approval from the West of Scotland Research Ethics Service (WoSRES) on 20th January 202, REC reference: 20/WS/0179. Results will be submitted for publication to a peer-reviewed journal.

Trial registration number

ISRCTN16922410, Pre-results

Keywords: Alcohol. FibroScan. Alcohol related liver disease. Alcohol recovery stories

For peer review only

Strengths and limitations of the trial

- The KLIFAD trial is the first randomised control trial to evaluate the feasibility of using non-invasive liver stiffness measurement as a behavioural intervention.
- The KLIFAD trial is the first randomised control trial to incorporate alcohol recovery video stories.
- The mixed methods design of the KLIFAD trial will enable us to test the acceptability of trial specific procedures to participants and key alcohol workers.
- The trial will enable a definitive KLIFAD trial to establish the effectiveness of non-invasive screening for liver fibrosis in community alcohol services.
- The primary limitation of the KLIFAD trial is that it is a single centre trial which could limit generalisation of findings.

For peer review only

Introduction

Alcohol-related liver disease (ARLD) is the most common cause of cirrhosis in United Kingdom (UK), and mortality from ARLD has risen significantly in the last three decades. It is now the second most common cause of working life years lost in men and fifth in women [1,2]. Europe has one of the highest prevalence of Alcohol Use Disorders (AUD) involving 15% of men and 3.5% of women[2]. Around 25% of the UK population drink above the UK specific recommended level of 14 units per week, and 10% are harmful drinkers[3]. The total per capita pure alcohol intake in UK for people age ≥15year is 11.4 litres/annum per person, which is twice higher than globally reported 6.4 litres/annum per person[2,3]. Approximately 20-30% of lifelong drinkers develop liver cirrhosis, and the risk is even higher (35%) among harmful drinkers [4,5].

ARLD causes no symptoms in its earlier stages; indeed, patients are often unaware they have serious physical health problems until they present with the complications of cirrhosis for example; ascites, jaundice, encephalopathy, variceal bleed, and liver failure, when the opportunity for treatment and recovery of liver health are significantly reduced [1,5,6]. It is estimated that the cost to the UK of alcohol on health is £3.5 billion per year [3,7], consuming 3.6% of the National Health Service (NHS) annual budget [8]. In England, there were 5,698 alcohol-specific deaths in 2018, the alcohol-specific age-standardised death rate was 11.9/100,000 (male=16.4 female=7.6), Nottingham (UK) has one of the highest (total=18.6, male=26.8, female 10.2) alcohol-specific age-standardised death rate/100,000 in the country [9]. A recent trial from the United States (US) predicted a 75% increase in age-standardised annual mortality and a 65% increase in decompensated cirrhosis due to ARLD over the next two decades[10].

Systematic reviews of Randomised Controlled Trials (RCTs) have established that delivering brief advice about alcohol to harmful drinkers helps them reduce their alcohol consumption[11,12]. Most studies were conducted in primary care settings where the prevalence of liver disease is likely to be markedly lower than in specialist alcohol treatment services. In alcohol services, where high levels of physical and psychological dependence on alcohol are frequent, National Institute of Clinical Excellence (NICE) guidelines state adults with high levels of alcohol dependency should be assessed and offered intensive structured community-based interventions (with or without medical therapy) as these provide the best chance of achieving and maintaining abstinence from alcohol[13]. Most clinical services in the UK are based on these principles. Individual programmes vary by locality with many of these services delivered by non-NHS providers. Despite brief advice and other alcohol-related interventions delivered in clinical practice for over two decades, mortality and morbidity due to alcohol misuse continue to rise in the UK[3]. There is a pressing need to optimise existing interventions to reduce harmful alcohol intake and examine effective alternative options.

Early diagnosis of liver fibrosis provides an opportunity to intervene and reduce or stop alcohol intake. This is known to be the most effective way of preventing liver disease progression [14]. Transient elastography by FibroScan (Echosens, France) has been used in primary care (General Practice) settings to detect liver disease in populations identified as having liver disease risk (heavy drinkers and those with type 2 diabetes). These studies showed that screening asymptomatic individuals based on risk for liver disease doubled the rates of liver cirrhosis diagnosis in the primary care populations studied[15,16]. Moreover, a recent systematic review suggested providing feedback to patients based on markers of liver injury can be an effective way to reduce harmful alcohol intake[17]. The addition of recovery stories helps one's mental health illness and addiction recovery [18,19]. The peer support from people who have

For peer review only - http://bmjopen.bmj.com/site/about/guidelines.xhtml

recovered from alcohol misuse had been proven beneficial in modifying high risk drinking behaviour[20].

This trial aims to investigate the feasibility and acceptability of conducting an RCT in community specialist alcohol services settings run by Nottingham Recovery Network (NRN) and to test the acceptability of trial interventions (FibroScan and Alcohol Recovery Video Stories, ARVS).

Selection of term alcohol misuse

We acknowledged the heterogeneity in language used to describe alcohol use and stigma associated with some of these terms, which itself can act as barrier to change[21]. Some of terms like alcohol use disorder (AUD) are not well understood among general population. The original research idea for the current research project was put forward in collaboration with patient and population representative group (PPI). After thoughtful discussion between study and PPI groups, we opted term **'alcohol misuse'** to describe excess alcohol intake, harmful alcohol intake, drinking problems, alcohol dependence, and AUD.

Alcohol misuse was defined as, "weekly alcohol intake ≥14 units, or an AUDIT score of ≥ 8, or key alcohol worker and/or physician diagnosis, or referral from any other services with problem drinking".

Methods and analysis:

KLIFAD is a parallel design feasibility RCT. The trial will be conducted in a single centre in the UK, carried out at three community alcohol services in Nottingham (the Wellbeing Hub, Edwin House and the Primary Care Alcohol Clinic run by the Nottingham Recovery Network) hosted by Framework and Nottingham Recovery Network (NRN) and working in partnership with Nottinghamshire NHS Foundation Trust.

The KLIFAD trial consists of three work packages (WP) (Figure 1).

Work Package one (WP1)

WP1 aims to design a standardised script framework for FibroScan operators to deliver liver disease-specific advice to participants having FibroScan as part of the feasibility RCT (WP3).

FibroScan, is an ultrasound technology developed by Echosence, France, which non-invasively asseses liver stiffness. A prototype script for FibroScan has been created in consultation with the existing KLIFAD Patient Public Involvement (PPI) group covering three ranges of FibroScan scores, normal ≤7 Kilopascal (kPa), intermediate fibrosis 8-15 kPa and advance fibrosis ≥15 kPa. The sample of these scripts are provided in supplementary material (SP) and the trial flow chart in Figure 2.

We will organise separate participant and FibroScan operator focus groups to collect feedback on the prototype scripts. The participant focus group will allow us to investigate the key messages to be included in the script and feedback, as well as considering how best to present the FibroScan results (e.g., graphically, in the text). The FibroScan operator focus group to investigate implementations in clinical practice. In addition, to evaluate the stages of change, a validated readiness to change model will be piloted[22].

Following Krueger's (1988) focus group guide, each focus group will include five-eight participants and will last for a maximum of two hours[23]. Depending upon the latest Covid-19 guidelines the focus group will be either virtual or face-to-face. A topic guide will be used (SP-Focus Group Guide WP1 V2.0). We aim to arrange two participant focus groups and one

For peer review only - http://bmjopen.bmj.com/site/about/guidelines.xhtml

FibroScan operator focus group. The focus groups will be facilitated by two members of the research team. Examples of questions include:

a) If you were a participant in the trial, would the script make sense to you?
b) Are there any parts of the script that you do not understand, and if so, why?
c) What is the best way to present the results of the FibroScan (e.g., graphically, in the text)?

Eligible participants (Table 1) will be identified and recruited through multiple channels. For example, via existing patient forums at all three recruitment settings, the KLIFAD PPI group, by offering information to patients self-presenting to any of the trial treatment settings, snowball methods, and via Black, Asian and minority ethnicity/Framework PPI groups. The focus group meeting will be recorded and transcribed verbatim either by automated software or an independent sponsor approved transcriber. After the first participant focus group the FibroScan script will be edited considering feedback and a second focus group will then be held to review iterated scripts. The final scripts will be sent via email to participants of focus groups for any final thoughts. We will then organise a FibroScan operator focus group of key alcohol workers working at any of the recruitment settings who are willing to give informed consent, to discuss any specific implementation issues.

After the focus groups, we will collect participant feedback on the change model (SP-Change model questionnaire (CMQ) V1.0) to get an initial sense of the applicability of readiness to change following discussion about the scripts.

**Work package one**	
**Inclusion criteria**	**Exclusion criteria**
A person age ≥18 years	Other primary substance misuse even where alcohol is a factor
Primary problem of alcohol misuse[a]	Lacks capacity to confirm consent
Had FibroScan in past	
**Work package two**	
**Inclusion criteria**	**Exclusion criteria**
A person age ≥18 years	Lacks capacity to confirm consent
Primary problem of alcohol misuse	
Had FibroScan in past	
A with lived experience of alcohol problems	
A person Willing to consent to the recording and public use of video recording	
**Work package three the randomisation phase**	

For peer review only - http://bmjopen.bmj.com/site/about/guidelines.xhtml

Inclusion criteria	Exclusion criteria
A person age ≥18 years	Other primary substance misuse even where alcohol is a factor
Primary problem of alcohol misuse	Lacks capacity to confirm consent
	Referrals from driving offences and student referrals[b]
	Out of area clients at Edwin House[c]
	Participants unable to comply with trial procedures

Table 1: KLIFAD trial eligibility criteria

[a]Alcohol misuse was defined as, weekly alcohol intake ≥14 units, or an AUDIT score of ≥ 8, or key alcohol worker and/or physician diagnosis, or referral from any other services with problem drinking.

[b]As these individuals are essentially not self-presenting, may have different motivation and have lower overall levels of alcohol use and so are substantially lower risk of having liver disease.

[c]In whom we cannot obtain follow up data due to lack of follow up availability.

Work Package Two (WP2)

WP2 aims to create a video library of ARVS from people with a history of alcohol misuse. These ARVS will be used in the feasibility RCT (WP3).

Receiving mental health recovery stories can provide benefits to some people experiencing mental health distress [18,24,25], and the effectiveness of mental health recovery stories as an intervention to increase quality of life has been examined in a clinical trial[26]. However, equivalent evidence is not available for the impact of ARVS. So that we can explore the impact of stories of recovery from alcohol misuse, in WP2 we will develop a set of recovery stories from participants who have successfully overcome their alcohol misuse. These videos will be peer-reviewed by the KLIFAD PPI group which will include input from Nottingham University Hospitals NHS Trust (NUH) Black, Asian and minority Ethnic patient and public involvement Group. Based on feedback the videos will then be edited ready for use in the feasibility RCT (WP3). All edits will be agreed upon with the story narrators.

For each narrator, we will follow their preference to create either:

- A recovery story that starts with an open-ended question where narrators have the liberty to tell their story without interruption *or*
- A recovery story in which the participant is asked a set of standard questions.

Drinking history and last FibroScan reading will be recorded at the start. Eligible participants (Table 1) will be recruited through the channels used in WP1. Those who took part in WP1 will also be invited to take part in WP2. A purposive sample based on demographic and liver disease severity of 6-9 individuals will be selected[27]. We will arrange a meeting with the KLIFAD PPI group to discuss what makes a video impactful. The outline of WP2 is given in Figure 2.

For peer review only - http://bmjopen.bmj.com/site/about/guidelines.xhtml

The ARVS will be recorded either at NDDC Biomedical Research Centre Nottingham University Hospital, the University of Nottingham, or the participant's usual place of residence. Each video will be of two-to-five-minute duration. Videos will be titled based on FibroScan score (low-risk, medium and high-risk score). Videos will be subtitled and depending on the final video format after the feedback we envisage adding a photograph of the storyteller and a short-associated text on the title page with informed consent from the participant. The video stories will be brought together in a single tablet computer-based package from which the participant will be able to choose their most preferred video after receiving a FibroScan score. Collaborative work between a clinician and patient can make a significant impact on the recovery process [28] and hence in some videos, and with consent by narrators, we will include sections of a video narrated by a clinician the narrator has worked with.

All video stories recorded as part of the KLIFAD trial will have peer review by the trial team and KLIFAD/Black, Asian and ethnic minority PPI groups. The videos will be shown in the same format that they would be used in WP3.

Work Package 3 (WP3)- Feasibility RCT

A feasibility RCT of parallel groups (one-to-one) will compare usual care (assessment and entry into an alcohol reduction programme which does not include information on liver disease severity) to usual care plus feedback from the FibroScan and ARVS. The eligibility for WP3 is provided in Table 1 and the attached flow chart (Figure 3).

Objectives

Bowen et al (2009)'s guide for feasibility studies was used to decide objectives [29].

1. **Test:** the intervention (FibroScan plus feedback and ARVS) in a feasibility randomised control trial.
2. **Acceptability**: of feasibility randomised control trial related procedures and interventions among patients and healthcare workers.
3. **Feasibility outcomes**: to establish recruitment rate, consent rate, dropout rate, and completion rate for accurate sample size calculation for future large-scale RCT.
4. **Refine**: the eligibility and randomisation criteria for a future large-scale RCT.
5. **Implementation and practicality:** to assess the ability of community alcohol services to deliver the intervention, and training and support needs for community alcohol services keyworkers for delivering the intervention.
6. **Adaptation:** of KLIFAD Trial interventions, FibroScan feedback, and ARVS format and access as per suggestions from participants and key alcohol workers
7. **Limited efficacy**: to test limited efficacy of KLIFAD interventions

Intervention Group

Participants randomised to the intervention arm will receive a FibroScan, feedback on FibroScan results and watch ARVS immediately after. The ARVS will be made available should a participant wish to watch them later.

Control group

Participants randomised to the control arm will continue with standard treatment (usual care) provided at the three treatment settings. The participants in this arm will be offered FibroScan at 6 months.

For peer review only - http://bmjopen.bmj.com/site/about/guidelines.xhtml

As part of standard treatment, the recruitment settings provide different types of interventions to participants in line with the National Drug Treatment Monitoring System Dataset (NDTMS) and Public health England (PHE) guidelines [30]. Existing treatment programmes can run for up to 12-weeks.

For adult drug and alcohol services there are three main categories of standard intervention (usual care) delivered by the NRN:

a) Psychological: which includes motivational interventions, family and social network interventions, and cognitive and behavioural based relapse prevention interventions (substance misuse specific).
b) Recovery Support: which includes 12 step work and counselling.
c) Pharmacological: which involves prescribing medication for drug and/or alcohol relapse prevention support. For example, naltrexone, acamprosate, disulfiram as part of alcohol or opioid relapse prevention therapy and Chlordiazepoxide for acute alcohol withdrawal.

Specific treatment programmes are started after an initial assessment and based on the participant's needs. The duration of contact with services varies, most participants stay with services for 12 weeks, some get discharged early, and a few stays longer than six months.

Methods

Sample size

As this is a feasibility trial, a formal sample size calculation for between-group comparisons of a primary outcome is not appropriate. . Researchers have previously recommended sample sizes between 24-50 to satisfactorily achieve feasibility outcomes [31-33].

After discussion with community alcohol services data manger and considering variation in number of patients presenting per week, we aim to approach 40 eligible participants per month. Assuming a 50% consent rate we anticipate randomising 20 participants per month (10 per month per arm) for a recruitment period of six months. With an estimated sample size of 120, we will be able to calculate a dropout rate of 80% within in a 95% confidence interval of +/-7.1%. Assuming a non-differential follow-rate of 80%, this target sample size should provide follow-up outcome data on a minimum of 48 participants in each of the two arms.

Randomisation

The participants will be individually allocated on a one-to-one ratio using minimisation with a probabilistic element. The minimisation variables will be age, gender, ethnicity, and severity of alcohol misuse based on the Severity of Alcohol Dependence Questionnaire (SADQ) score. To minimise the selection bias the randomisation is externally performed by data manager from Nottingham Recovery Network.

Schedule of visits

Baseline

The baseline visits will be on the day when the participant starts standard treatment at any recruitment setting. At this visit written informed consent will be given by participants and participants will be randomised to the intervention or control group. Participants in both arms will have an initial detailed assessment (SP-NRN assessment form Supplementary Material) as part of their standard care. This includes the collection of baseline demographic and clinical data (e.g., age, gender, ethnicity). Participants randomised to the control arm will continue

For peer review only - http://bmjopen.bmj.com/site/about/guidelines.xhtml

with usual care while participants randomised to the intervention arm will have the usual care and FibroScan followed by standardised script feedback with ARVS watched immediately after the FibroScan result.

Three months

This visit will be part of usual care no research specific activity will be carried out. The research data will be extracted from routinely collected data from three treatment settings.

Six months

This will be a telephone consultation or in-person appointment by the research team. Participants in the control arm will be offered a FibroScan after completion of outcomes. The six-month follow up is specifically to cover those who were lost to follow up at NRN from the treatment programme.

A detailed schedule of the visits is given in Table 2.

For peer review only

For peer review only - http://bmjopen.bmj.com/site/about/guidelines.xhtml

Trial Activity	Baseline visit	3[a] Months	6[b] months
**Control group**			
Date & Time	Yes	Yes	Yes
Baseline consent	Yes	-	-
FibroScan + Feedback	-	-	Yes
Watching video stories	-	-	Yes
Qualitative interview	-	-	Yes
Demographics	Yes	-	-
AUDIT score	Yes	Yes	Yes
SADQ score	Yes	Yes	Yes
Self-reported alcohol intake[c]	Yes	Yes	Yes
Breath alcohol test	Yes	Yes	Yes
Substance misuse other than alcohol	Yes	Yes	Yes
Data on feasibility outcomes	Yes	Yes	Yes
**Intervention group**			
Date & Time	Yes	Yes	Yes
Baseline consent	Yes	-	-
FibroScan + Feedback	Yes	-	-
Watching video stories	Yes	-	-
Qualitative interview	-	-	Yes
Demographics	Yes	-	-
AUDIT score	Yes	Yes	Yes
SADQ score	Yes	Yes	Yes
Self-reported alcohol intake	Yes	Yes	Yes
Breath alcohol test	Yes	Yes	Yes
Substance misuse other than alcohol	Yes	Yes	Yes
Data on feasibility outcomes	Yes	Yes	Yes

Table 2: Work-package-three (feasibility RCT) schedule of visits and variables for data
(Alcohol Use Disorder Identification Test- AUDIT, Severity of alcohol dependence questionnaire- SADQ)

[a]3-months visit: this will be routine visit no trial-specific procedure will be carried out

[b]6 -months visit: will be a telephone consultation and/or if possible/required in person

The participant in the control group will be offered a FibroScan at 6 months if they attend it will be in-person appointment

[c]Self-reported alcohol intake in gram and unites per week

For peer review only - http://bmjopen.bmj.com/site/about/guidelines.xhtml

Data collection

At Baseline, three and six months, the following data will be collected (Table 2)

- Demographics (including address, email address and contact number) This will be archived and kept separate from the main database.
- Alcohol Use Disorder Identification Test (AUDIT) scores.
- Severity of Alcohol Dependence Questionnaire (SADQ) scores.
- Self-reported alcohol intake (gram and unit per week).
- Substance misuse other than alcohol.
- Breath alcohol testing where participants are still attending.
  Breath alcohol testing is a strength of this trial; most studies have relied on self-reporting of alcohol intake. This means we can correlate breath alcohol readings with self-reporting, providing substantial additional information.
- Data on feasibility outcomes (e.g., screening rate, recruitment rate, retention rate).

All the above measurements are part of routine outcomes data collected by all three recruitment settings, apart from the six-month data collected for those who are no longer in a treatment programme at six months. All three services included in this trial record all the above outcomes as part of the 12-week programme standard data set and report these to commissioners. Follow-up data is obtained at every attendance and includes the above dataset and breath alcohol testing.

Qualitative data

We will conduct one-to-one semi-structured interviews to evaluate participant's experiences of being part of the trial (e.g., "Overall, how do you feel about taking part in the KLIFAD trial?") and any changes they may have made to their lives (e.g., "Do you think the KLIFAD trial changed your use of alcohol in any way?"). The preliminary qualitative interview schedule topic guide is provided in supplementary material (SP- semi-structure interview). It will be piloted before use by the PPI group to check structure and wording of questions. A readiness to change model used in WP1 will also be piloted. Focus groups and interviews will be audio-recorded and transcribed by an independent transcriber approved by the sponsor for thematic analysis.

Health economics

Routine NHS data which is collected for the standard care 12-week treatment programmes will be used together with resources utilisation derived from the NHS digital linked data to derive healthcare costs and the potential benefits of the intervention.

Outcomes

The outcomes are designed to assess the feasibility and acceptability of the KLIFAD intervention and research processes to help inform a future large-scale RCT. The following outcomes will be reported:

- Recruitment rate.
- Retention rate.
- Consent rate.
- Acceptability of the intervention (FibroScan and ARVS).
- The willingness of participants to be randomised to trial arms.
- Acceptability of the intervention to patients.

For peer review only - http://bmjopen.bmj.com/site/about/guidelines.xhtml

For peer review only

- Participant adherence.
- Feasibility of outcome measures.

These feasibility outcomes will enable the trial team to:

- Determine the best primary endpoint for the future definitive trial.
- Provide sample size estimates for the future definitive trial.
- Record ARVS which will contribute to the video library used in later large-scale RCT.

Statistical and data analysis plan

The analyses of the quantitative data will be in line with Consolidated Standards of Reporting Trials (CONSORT) guidelines for pilot and feasibility trials [34]. Sekhon et al's (2017) framework for acceptability testing will be used[35]. The primary descriptive analyses will be on an intention-to-treat basis (that is, participants are analysed in the group to which they were originally allocated). Data will be summarized using frequency (%), mean (SD) or median (IQR) depending on the distribution of the data. Summary measures will be presented along with their 95% confidence intervals whenever appropriate. Results of the data analysis will be presented using appropriate tables and graphs.

The trial is not powered to investigate statistical significance between the two arms. As this is a feasibility trial, no subgroup analysis is planned. However, the results of the feasibility variables will be presented by categories of different variables (age, gender, ethnicity, severity of alcohol misuse).

Different techniques will be followed to maximize the completeness of data collection (for example via staff training). The level of missing data will be assessed. This is especially useful for the proposed primary outcome variables. An interim analysis is not planned for this trial, but the progress of the trial will be reported to the oversight committee who can assess any concerns.

Thematic analysis of qualitative data will be conducted following Braun and Clarke's standard methods[36]. Care will be taken to integrate updated guidelines about thematic analysis including a transparent appreciation of researcher reflexivity[36]. If the trial management group feel the analysis requires external validity, a sample of transcripts identified by a random number generator with the codebook will be given to a researcher independent of the trial. This will allow us to calculate the % agreement and Cohen's Kappa value (using criteria by Cohen, 1960)[37]. The Consolidated Criteria for Reporting Qualitative Studies (COREQ) will be used to ensure thorough and explicit reporting of qualitative data in reports and manuscripts for publication [38].

Ethics and dissemination

Ethical approval

The trial received favourable ethical approval from the West of Scotland Research Ethics Service (WoSRES) on 20th January 2021, REC reference: 20/WS/0179.

Informed consent

All participants will provide a written or online (e-consent) informed consent before any research activities are initiated. A PIS written in plain language will be provided and it will be

For peer review only - http://bmjopen.bmj.com/site/about/guidelines.xhtml

ensured the participant has understood the trial information and had enough time to make an informed decision. The Site Investigator will be available to answer any questions about trial participation.

Data handling and record-keeping

In compliance with the ICH/Good Clinical Practice guidelines, regulations and following the Nottinghamshire Healthcare NHS Foundation Trust SOPS, the Chief or local Principal Investigator will maintain all records and documents regarding the conduct of the trial. These will be retained for at least 24 months or for longer if required. If the responsible investigator is no longer able to maintain the trial records, a second person will be nominated to take over this responsibility. The routinely collected clinical data will be treated in the same way as other clinical case records are treated in the NHS following Nottinghamshire Healthcare NHS Foundation Trust's, the Government's, and funders' policies.

The Trial Master File and trial documents held by the Chief Investigator on behalf of the Sponsor shall be finally archived at secure archive facilities at the Nottingham Digestive Diseases Biomedical Research Centre (NDDC) at Nottingham University Hospital NHS Trust (NUHT). This archive shall include all trial databases and associated meta-data encryption codes.

An index will be created for the CRF and paper trial data before it gets stored. All online and IT-based data will be password protected and access will only be granted to people directly involved in trial and data analysis. All patient identifiable data will be anonymised with trial-specific participant number.

The information will be copied to the research database (REDCAP cloud) run by the NUHT. We will delete any information that identifies participant by the end of the KLIFAD trial (currently expected October 2022). Moreover, we will ensure data security by following the UK data protection laws.

Participant safety

There is a risk that being given a normal FibroScan result may provide false reassurance and encourage the participant to maintain their current level of harmful drinking or encourage them to drink more. It is also possible that a high reading will generate anxiety. The trial is designed to minimise these risks by providing scripted feedback (WP1) and watching ARVS (WP2).

Cirrhosis diagnosis and FibroScan: It is anticipated that a small number of people will be identified who have previously unknown cirrhosis and so would be at risk of complications of liver disease. This will be mitigated by offering onward referral to out-patient Hepatology for all participants with a FibroScan reading >15 Kilopascal(kPa). This will be via contact with the participant's GP and would follow the current NUHT Nottinghamshire adult liver disease stratification pathway for referral[39]. Some mitigation of this risk will be done via the feedback included in this trial which covers cirrhosis.

We cannot foresee any potential risks except possible emotional distress during participation in a focus group or semi-structured interview. Participants can choose to skip any question

For peer review only - http://bmjopen.bmj.com/site/about/guidelines.xhtml

that they prefer not to answer. If distress occurs during the trial visit, we will ask the participant to take a break to recover or they can terminate the process. We do not expect that the trial will cause any discomfort or pose any disadvantages, however, contact details for the trial team are provided should the participant have any questions before, during, or after taking part. We have also provided a list of locally relevant support services at the end of each patient information sheet, which participant can consult.

Patient and public involvement (PPI)

The trail had dedicated PPI group and had considerable regular input from PPI group at every stage.

Dissemination

The results of the feasibility trial will be submitted for publication to a peer-reviewed journal and presented at relevant conferences. A separate manuscript on the qualitative aspect of the trial will be written as well. This work is part of a PhD for the lead author (MS) who will present and submit data as a PhD Thesis to the University of Nottingham. The work will also be made available to trial participants via the NDDC Biomedical Research Unit website.

For peer review only

References

1. Williams R, Aithal G, Alexander GJ, et al. Unacceptable failures: the final report of the Lancet Commission into liver disease in the UK. *Lancet (London, England).* 2020;395(10219):226-239.
2. WHO. <2018 WHO global stats report on alcohol and health.pdf>. 2018.
3. Williams R, Aspinall R, Bellis M, et al. Addressing liver disease in the UK: a blueprint for attaining excellence in health care and reducing premature mortality from lifestyle issues of excess consumption of alcohol, obesity, and viral hepatitis. *Lancet.* 2014;384(9958):1953-1997.
4. Osna NA, Donohue TM, Jr., Kharbanda KK. Alcoholic Liver Disease: Pathogenesis and Current Management. *Alcohol research : current reviews.* 2017;38(2):147-161.
5. BritishLiverTrust. <2019 British liver trust The-alarming-impact-of-liver-disease-FINAL-June-.pdf>. 2019
6. Parker R. The natural history of alcohol-related liver disease. *Current Opinion in Gastroenterology.* 2020;36(3).
7. England PH. <2019 Statistics on Alcohol, England 2019 [PAS] - NHS Digital.pdf>. 2019.
8. <House of Commons Health Committee. Government's Alcohol Strategy. Third Report of Session.pdf>. 2012.
9. England PH. Statistics on Alcohol, England 2020. 2020.
10. Julien J, Ayer T, Bethea ED, Tapper EB, Chhatwal J. Projected prevalence and mortality associated with alcohol-related liver disease in the USA, 2019–40: a modelling study. *The Lancet Public Health.* 2020;5(6):e316-e323.
11. O'Donnell A, Anderson P, Newbury-Birch D, et al. The impact of brief alcohol interventions in primary healthcare: a systematic review of reviews. *Alcohol and alcoholism (Oxford, Oxfordshire).* 2014;49(1):66-78.
12. Kaner EF, Beyer F, Dickinson HO, et al. Effectiveness of brief alcohol interventions in primary care populations. *The Cochrane database of systematic reviews.* 2007(2):Cd004148.
13. NICE. Alcohol-use disorders: prevention. 2010.
14. Roberts SE, John A, Brown J, Napier DJ, Lyons RA, Williams JG. Early and late mortality following unscheduled admissions for severe liver disease across England and Wales. *Aliment Pharmacol Ther.* 2019;49(10):1334-1345.
15. Harman DJ, Ryder SD, James MW, et al. Direct targeting of risk factors significantly increases the detection of liver cirrhosis in primary care: a cross-sectional diagnostic study utilising transient elastography. *BMJ Open.* 2015;5(4):e007516.
16. Harman DJ, Ryder SD, James MW, et al. Obesity and type 2 diabetes are important risk factors underlying previously undiagnosed cirrhosis in general practice: a cross-sectional study using transient elastography. *Aliment Pharmacol Ther.* 2018;47(4):504-515.
17. Subhani M, Knight H, Ryder S, Morling JR. Does Advice Based on Biomarkers of Liver Injury or Non-Invasive Tests of Liver Fibrosis Impact High-Risk Drinking Behaviour: A Systematic Review With Meta-analysis. *Alcohol and Alcoholism.* 2021.
18. Rennick-Egglestone S, Ramsay A, McGranahan R, et al. The impact of mental health recovery narratives on recipients experiencing mental health problems: Qualitative analysis and change model. *PloS one.* 2019;14(12):e0226201-e0226201.
19. White WL. Addiction recovery: its definition and conceptual boundaries. *Journal of substance abuse treatment.* 2007;33(3):229-241.
20. Tracy K, Wallace SP. Benefits of peer support groups in the treatment of addiction. *Subst Abuse Rehabil.* 2016;7:143-154.
21. Ashford RD, Brown AM, Curtis B. Substance use, recovery, and linguistics: The impact of word choice on explicit and implicit bias. *Drug Alcohol Depend.* 2018;189:131-138.
22. Zimmerman GL, Olsen CG, Bosworth MF. A 'stages of change' approach to helping patients change behavior. *Am Fam Physician.* 2000;61(5):1409-1416.

For peer review only - http://bmjopen.bmj.com/site/about/guidelines.xhtml

23. Krueger RA. *Focus groups: A practical guide for applied research.* Thousand Oaks, CA, US: Sage Publications, Inc; 1988.
24. Rennick-Egglestone S, Morgan K, Llewellyn-Beardsley J, et al. Mental Health Recovery Narratives and Their Impact on Recipients: Systematic Review and Narrative Synthesis. *Canadian journal of psychiatry Revue canadienne de psychiatrie.* 2019;64(10):669-679.
25. Ng F, Charles A, Pollock K, et al. The mechanisms and processes of connection: developing a causal chain model capturing impacts of receiving recorded mental health recovery narratives. *BMC psychiatry.* 2019;19(1):413-413.
26. Rennick-Egglestone S, Elliott R, Smuk M, et al. Impact of receiving recorded mental health recovery narratives on quality of life in people experiencing psychosis, people experiencing other mental health problems and for informal carers: Narrative Experiences Online (NEON) study protocol for three randomised controlled trials. *Trials.* 2020;21(1):661.
27. Charles Teddlie FY. Mixed Methods Sampling A Typology With Examples. *Journal of Mixed Methods Research.* 2007;1(1).
28. STOTLAND NL, MATTSON MG, BERGESON S. The Recovery Concept: Clinician and Consumer Perspectives. *Journal of Psychiatric Practice®.* 2008;14:45-54.
29. Bowen DJ, Kreuter M, Spring B, et al. How we design feasibility studies. *Am J Prev Med.* 2009;36(5):452-457.
30. England PH. The international evidence on the prevention of drug and alcohol use Summary and examples of implementation in England. https://assets.publishing.service.gov.uk/government/uploads/system/uploads/attachment_data/file/774743/Preventing_drug_and_alcohol_misuse__international_evidence_and_implementation_examples.pdf. Published 2015. Accessed.
31. Sim J, Lewis M. The size of a pilot study for a clinical trial should be calculated in relation to considerations of precision and efficiency. *Journal of clinical epidemiology.* 2012;65(3):301-308.
32. Lancaster GA, Dodd S, Williamson PR. Design and analysis of pilot studies: recommendations for good practice. *Journal of evaluation in clinical practice.* 2004;10(2):307-312.
33. Browne RH. On the use of a pilot sample for sample size determination. *Statistics in medicine.* 1995;14(17):1933-1940.
34. CONSORT. Consort Statement
35. Sekhon M, Cartwright M, Francis JJ. Acceptability of healthcare interventions: an overview of reviews and development of a theoretical framework. *BMC Health Services Research.* 2017;17(1):88.
36. Braun V, Clarke V. Reflecting on reflexive thematic analysis. *Qualitative Research in Sport, Exercise and Health.* 2019;11(4):589-597.
37. Cohen J. A Coefficient of Agreement for Nominal Scales. *Educational and Psychological Measurement.* 1960;20(1):37-46.
38. Tong A, Sainsbury P, Craig J. Consolidated criteria for reporting qualitative research (COREQ): a 32-item checklist for interviews and focus groups. *International Journal for Quality in Health Care.* 2007;19(6):349-357.
39. Chalmers J, Wilkes E, Harris R, et al. The Development and Implementation of a Commissioned Pathway for the Identification and Stratification of Liver Disease in the Community. *Frontline Gastroenterol.* 2020;11(2):86-92.

For peer review only

Authors' contributions

Mohsan Subhani:

KLIFAD trial: The project is part of PhD thesis. Trial coordinator and member of trial management group. He has and will contribute to following; research idea, funding application, PPI meetings, trial protocol, IRAS application and ethical approval, FibroScan training, site initiation, work package 1 focus group, work package 2 alcohol recovery story recording, monthly trial management group meeting, monitoring ongoing progress of work package 3, qualitative interview, data synthesis and analysis, report writing, dissemination.

Manuscript: Written initial draft of the protocol, implemented changes, and drafted final version of protocol and manuscript.

Katy Jones:

KLIFAD trial: Member of trial management group. She is supervising the qualitative component of the trial including conducting and analysing semi structured interviews.

Manuscript: Reviewed protocol and manuscript, provided specialist input for qualitative aspects of the protocol and contributed to the final manuscript.

Kirsty Sprange:

KLIFAD trial: Member of trial management group. She contributed to following; research idea, funding application, trial protocol, IRAS application, work package 3 initiation, trial management and progress.

Manuscript: Reviewed protocol and manuscript and contributed to the final manuscript.

Stefan Rennick-Egglestone:

KLIFAD trial: Member of trial management group. He is supervising work package 2 including proposal for alcohol recovery stories recording, editing, and finalising.

Manuscript: Reviewed protocol and manuscript, provided specialist input for work-package-2 of protocol and contributed to final manuscript

Holly Knight:

KLIFAD trial: Member of trial management group. She is contributing to work package 1 including developing FibroScan results feedback scripts and organising focus groups.

Manuscript: Reviewed protocol and manuscript, provided specialist input for work-package-1 of protocol and contributed to final manuscript

Joanne R Morling:

KLIFAD trial: Member of trial management group. She PhD supervisor for Dr Subhani, supervising trial overall and specifically helping with health economics part of trial.

Manuscript: Reviewed protocol and manuscript, provided specialist input for health economics section of protocol and contributed to final manuscript

Doyo G Enki:

KLIFAD trial: Member of trial management group. He is Statistical support for the trial.

Manuscript: reviewed final manuscript

Andrew Wragg:

KLIFAD trial: Patient and public involvement coordinator.

Manuscript: reviewed final manuscript

Stephen D Ryder:

KLIFAD trial: Chief investigator, PhD supervisor for Dr Subhani and member of trial management group. He has contributed to following; research idea, funding application, PPI meetings, trial protocol, IRAS application and ethical approval, overall supervision of all three work packages, data synthesis and analysis, report writing, dissemination.

Manuscript: reviewed final manuscript

For peer review only

For peer review only - http://bmjopen.bmj.com/site/about/guidelines.xhtml

Funding statement

This work was supported by “National Institute for Health Research (NIHR)” grant number [RfPB NIHR201146]. JRM receives salary support from a Medical Research Council Clinician Scientist award (grant number MR/P008348/1).

Data sharing statement

The anonymised data that will support the findings of this trial will be available from the corresponding author, [MS], upon reasonable request.

Competing interests’ statement

No competing interests from any author

For peer review only

Figure Legends

Figure 1: The KLIFAD Trial timeline and flow chart (RCT-randomised control trial, GCP-good clinical practice, AUDIT-alcohol use disorder identification test, SADQ-severity of alcohol dependence questionnaire). The trial has three work packages (WP), WP3 is in randomised feasibility trial.

Figure 2: Flow diagram for; Work package one to create FibroScan scripted feedback and Work package two to create alcohol recovery videos stories

Figure 3: Flow diagram for work-package-three the randomised control trial flow chart (RCT-randomised control trial, GCP-good clinical practice, AUDIT-alcohol use disorder identification test, SADQ-severity of alcohol dependence questionnaire). Work package three is feasibility randomised control trial.

For peer review only

For peer review only - http://bmjopen.bmj.com/site/about/guidelines.xhtml

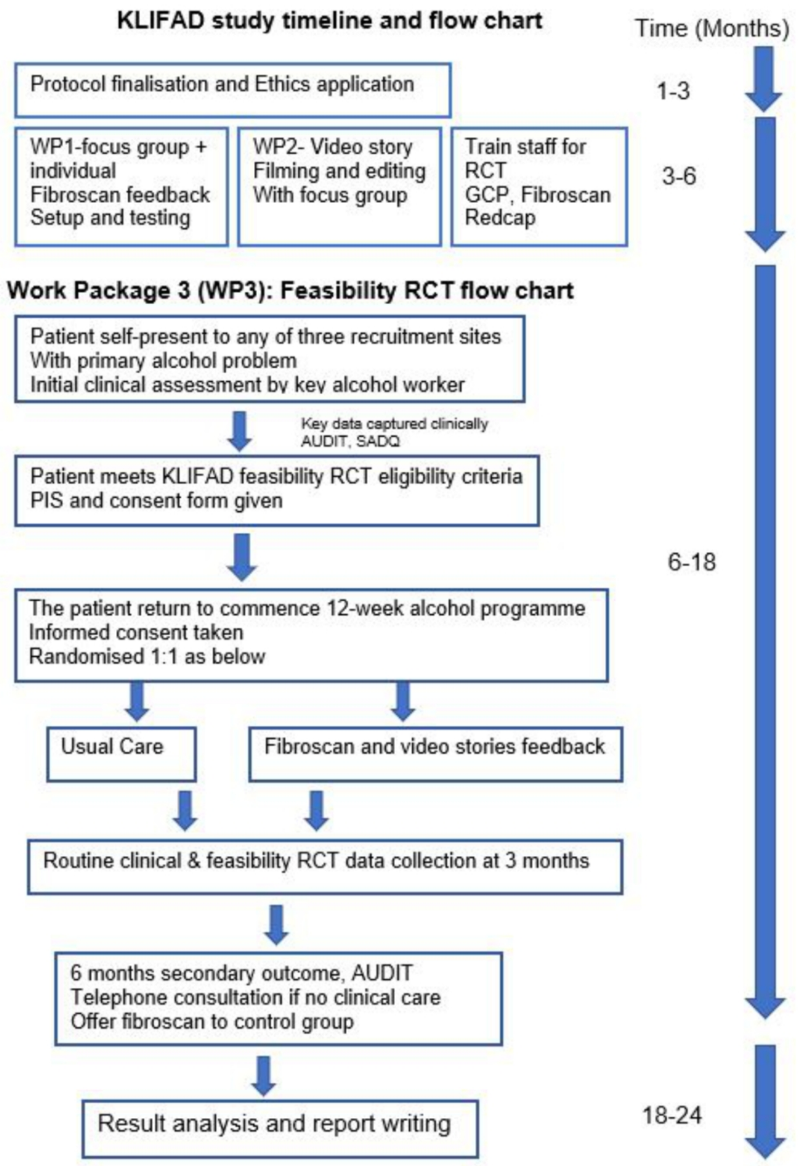

Figure 1: The KLIFAD trial timeline and flow chart (RCT-randomised control trial, GCP-good clinical practice, AUDIT-alcohol use disorder identification test, SADQ-severity of alcohol dependence questionnaire). The trial has three work packages (WP), WP3 is in randomised feasibility trial.

149x197mm (300 x 300 DPI)

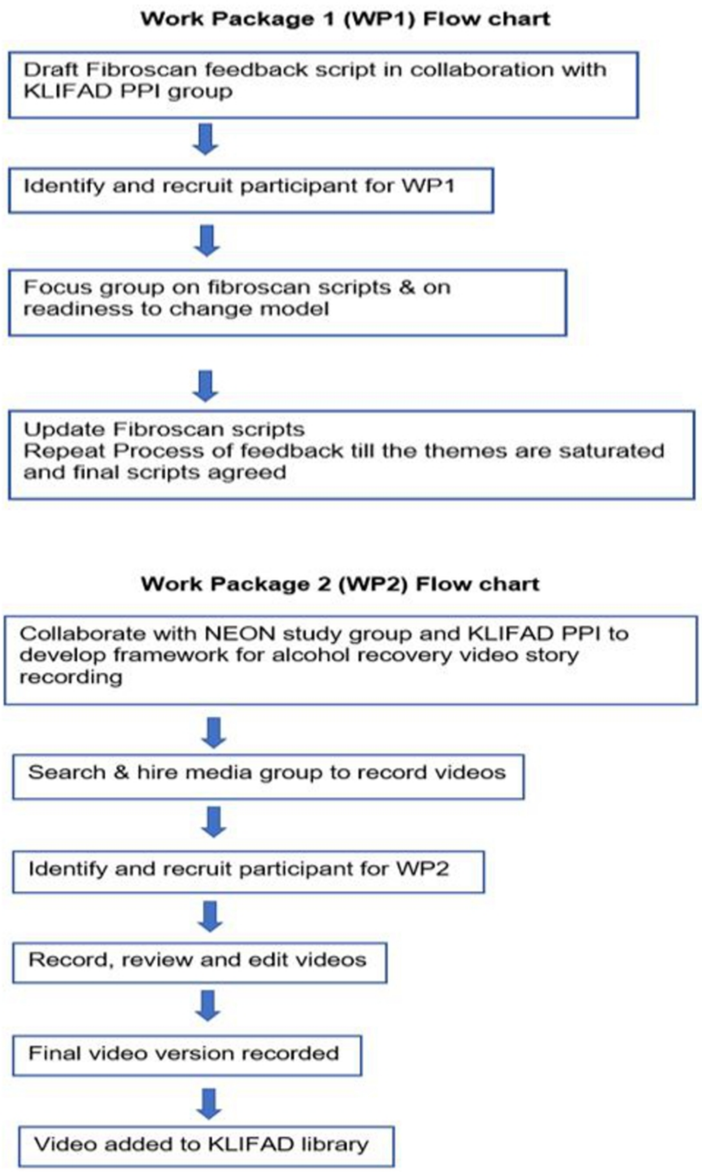

Figure 2: Flow diagram for; Work-package 1 to create fibroscan scripted feedback and Work-package 2 to create alcohol recovery videos stories

147x216mm (300 x 300 DPI)

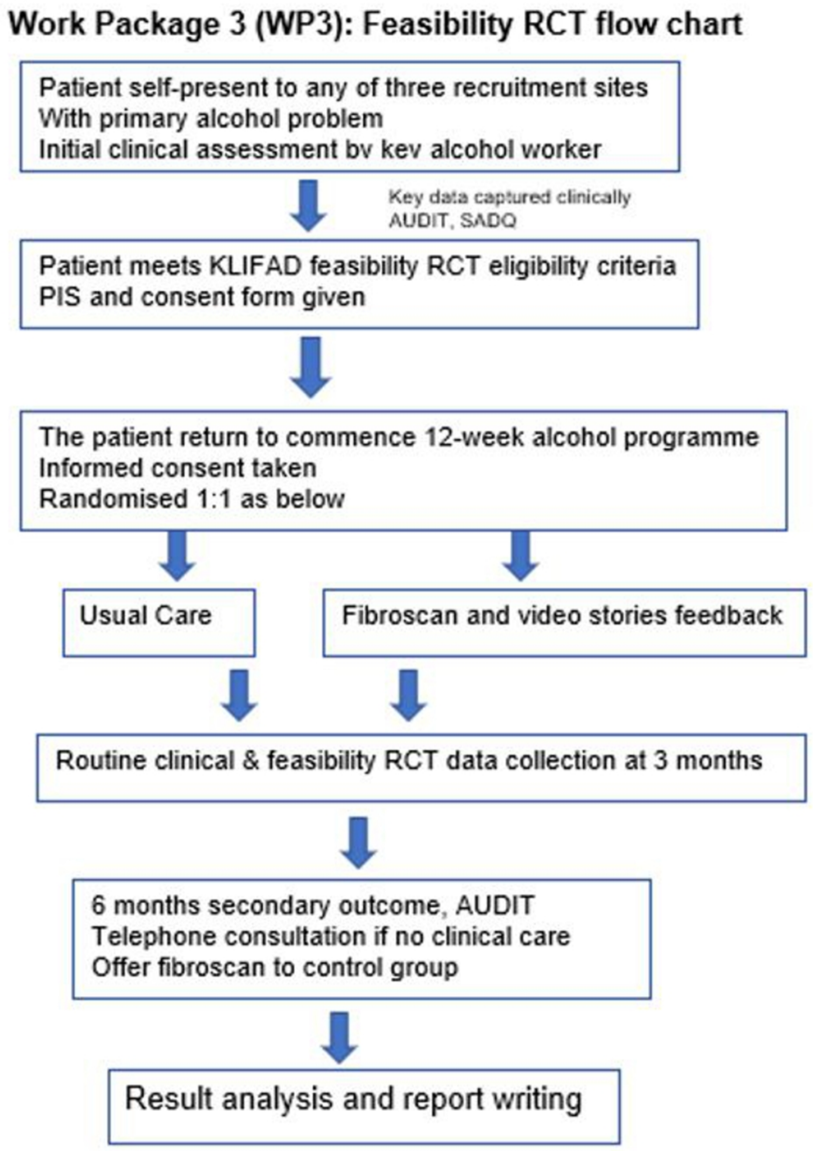

Figure 3: Flow diagram for work-package-3 the randomised control trial flow chart (RCT-randomised control trial, GCP-good clinical practice, AUDIT-alcohol use disorder identification test, SADQ-severity of alcohol dependence questionnaire). Work-package 3 is feasibility randomised control trial.

149x202mm (300 x 300 DPI)

Nottingham Recovery Network

Assessment Form

Clean Slate
Criminal Justice Substance Misuse Service

OFFICE USE ONLY

Date received:<br>Referred by:<br>If SELF, how did they hear about the service:		Client Id:<br>Assessed by:<br>Assessment location:<br>Date:
Specific risk / need identified:	Probation<br>Y / N	

CLIENT DETAILS

GDPR: There is a privacy notice in each room - please read it

DRUG ☐
DRUG & ALCOHOL ☐
ALCOHOL ☐

If drug(s) please state type:

Title: Mr / Mrs / Ms / Miss / other (please state)

First name: **Surname:**

Prefers to be known as:

Gender: Male ☐ Female ☐ Other ☐ Not specified ☐

Date of Birth: **Age:**

Address:

Postcode:

Home Tel:

Mobile Tel:

Email address:

G.P Name:

Address:

Tel:

Currently receiving treatment? Y / N
Currently on prescribed medication? Y / N
Seen by GP in last month? Y / N
GP aware of substance misuse? Y / N

Pharmacy current/preferred:

Permission to be contacted: Home Visit ☐ Letter ☐ Telephone ☐ Text ☐ Email ☐

Emergency Contact: **Telephone:**

Medication: Current ☐ Recent Past ☐ Past ☐ None ☐

Type & Dosage – List

....

....

Prescribed by:

Known Allergies: Y / N

List:

....

....

....

Barriers to Accessing Treatment? (include any disabilities)

Preferred Language:

Is an interpreter required Y / N

For peer review only - http://bmjopen.bmj.com/site/about/guidelines.xhtml

Client initials: DoB:

DEMOGRAPHICS

Sexuality: ☐ Heterosexual ☐ Gay/Lesbian ☐ Bi-sexual ☐ Other ☐ Not stated

Marital Status: ☐ Single ☐ Cohabiting ☐ Married ☐ Separated ☐ Divorced ☐ Widowed

Religion: ☐ None ☐ Christian ☐ Buddhist ☐ Hindu ☐ Jewish ☐ Muslim ☐ Sikh ☐ Other ☐ Not stated

Nationality: ☐ UK ☐ Other (please state) ....

Ethnic category

White	Mixed	Asian/Asian British	Black/Black British	Other Ethnic
British ☐	White & Black Caribbean ☐	Indian ☐	Caribbean ☐	Chinese ☐
Irish ☐	White & Black African ☐	Pakistani ☐	African ☐	Any Other ☐
Other White ☐	White & Asian ☐	Bangladeshi ☐	Black British ☐	Not Known ☐
	Other Mixed ☐	Other Asian ☐	Other Black ☐	Not Stated ☐

Accommodation:
☐ Homeless – urgent housing problems
☐ Housing problems –i.e. staying with friends
☐ Owner occupier
☐ Tenant - Landlord (please specify) ....

Employment Status:
☐ Employed (full/part time)
☐ Long term sick or disabled
☐ Retired from paid work
☐ Unemployed and seeking work
☐ Other (please specify)....................
☐ Homemaker
☐ Not receiving benefits
☐ Student
☐ Unpaid voluntary work

Time since last employed: ☐ Never employed ☐ Currently employed ☐ Less than 1 year ☐ 1 – 2 years ☐ 2 – 3 years ☐ >3 years (please state) ....

Sex Worker: Yes ☐ No ☐ **Current / Previous**
If **YES** working from premises or street ....

Ex Service Personnel: Yes ☐ No ☐ **Referral to Royal British Legion wanted?** Yes ☐ No ☐

Disability: Yes ☐ No ☐ Type ....

Carer: Yes ☐ No ☐ Support needs....................

Debt Issues: Yes ☐ No ☐ Support wanted ....

Support services already engaged with:

Support wanted:

Treatment Goal:

For peer review only - http://bmjopen.bmj.com/site/about/guidelines.xhtml

Client initials: DoB:

PRESENTATION ON ASSESSMENT

(Intoxicated / withdrawing / appearance etc.)

SUBSTANCE MISUSE PAST 7 DAYS / 4 WEEKS

For peer review only
For peer review only - http://bmjopen.bmj.com/site/about/guidelines.xhtml

Client initials: DoB:

SUBSTANCE MISUSE HISTORY

(Why, when, who with, route, frequency, finances, motivations, withdrawal symptoms, harm minimisation)

For peer review only

For peer review only - http://bmjopen.bmj.com/site/about/guidelines.xhtml

Client initials: DoB:

TREATMENT HISTORY

Currently in drug or alcohol treatment Yes ☐ No ☐

Name of all agencies/services currently in contact with client and keyworkers' names (where applicable):

Previously sought help with substance use Yes ☐ No ☐

Previously received structured drug or alcohol treatment Yes ☐ No ☐

Date of first treatment episode:

Further details

PROBLEMATIC SUBSTANCE USE

	Substance	Frequency (in last 28 days)	Amount/Units /Cost	Route	Age of 1st use
**Primary**					
**2nd**					
**3rd**					

Drug Screening Questionnaire (DAST)

Score ....

INJECTING STATUS

Currently injecting ☐ Previously ☐ Never injected ☐ Declined to answer ☐

ALCOHOL

Drinking Days .... /28 Units/day .... Units/week..

AUDIT .... SADQ....................................... Breath Alcohol: ....Mg/l BrAC

BBV

HIV Status: Negative ☐ Positive ☐ Not Known ☐ **Latest Test Date** ....

Hep C – Intervention Status

Offered and accepted ☐ **Hep C Tested** Yes ☐ No ☐ **Latest Test Date** ....

Offered and refused ☐

Not appropriate to offer ☐ **Hep C Positive** Yes ☐ No ☐ Not Known ☐

Hep B - Intervention Status

Offered and accepted ☐ **Vaccination Count:** 1 Vaccination ☐

Offered and refused ☐ 2 Vaccinations ☐

Immunised already ☐ 3 Vaccinations ☐

Not appropriate to offer ☐ Course Complete ☐

Referred For Hepatology Yes ☐ No ☐

For peer review only

Client initials: DoB:

PHYSICAL HEALTH

Does the client consider themselves to have a disability? Yes ☐ No ☐
Nature of disability

Does the client have any health problems? Yes ☐ No ☐
(allergies, asthma, epilepsy, diabetes, dental, women specific, sexual health, cardiac, respiratory, DVT, sleep, diet)

Smoker: No ☐ Yes ☐ Qty .... p/day

Does client have smoke alarm: Yes ☐ No ☐ **Referral made to Framework service**: Yes / declined

IMMEDIATE RISK IDENTIFIED: Physical Health & Overdose

Prompts: Regular injector / Injecting in high-risk areas / Poly substance use / Poly substance use – opiates and alcohol / Substance use related seizures or DTs? / Injects alone / Witnessed overdose by others / History of past overdose

Level of risk:	No risk ☐	Low ☐	Medium ☐	High ☐
Likelihood of occurrence	No risk ☐	Low ☐	Medium ☐	High ☐

ACTION(S):

For peer review only

For peer review only - http://bmjopen.bmj.com/site/about/guidelines.xhtml

Client initials: DoB:

PSYCHOLOGICAL HEALTH

Any current/historical contact with mental health services: Yes ☐ No ☐
(mental health diagnosis or symptoms, negative thoughts, self esteem, current mood, history of suicidal thoughts/acts, self-harm)

Mental Health treatment need Yes ☐ No ☐

Current services involved ....

Ever experienced overdose? Yes ☐ No ☐ Accidental ☐ Deliberate ☐

Date & Drugs involved

Treatment received

Has Naloxone been offered (please circle) **Accepted / Refused**

IMMEDIATE RISK IDENTIFIED: Individual

Prompts: Suicidal / Self-harm / Mental health / Domestic violence
Trigger: Where DV is identified complete Multi Agency Domestic Violence Risk Identification Form when appropriate

Level of risk: No risk ☐ Low ☐ Medium ☐ High ☐

Likelihood of occurrence No risk ☐ Low ☐ Medium ☐ High ☐

ACTION(S):

IMMEDIATE RISK IDENTIFIED: Personal Safety / Self Neglect

Prompts: Reliant on others / Difficulty in coping with everyday domestic tasks / Homeless/vulnerably housed / Recent threat (s) from others /Financial Vulnerability

Level of risk: No risk ☐ Low ☐ Medium ☐ High ☐

Likelihood of occurrence No risk ☐ Low ☐ Medium ☐ High ☐

DETAILS:

ACTION(S):

For peer review only - http://bmjopen.bmj.com/site/about/guidelines.xhtml

Client initials: DoB:

PARENTAL STATUS

Do you have any contact with children under 18yrs?: Yes ☐ No ☐

Children/Partner's Children: Yes ☐ No ☐ **Sole Carer:** Yes ☐ No ☐ **Other** ....

Do all/some of the children live with you?: All of the time ☐ Some of the time ☐ No ☐

No. of children: **Ages:**

Are any of the client's children (biological, step, foster, adoptive, guardian) or any of the children receiving early help or are they in contact with Children's Social Care?:

Child in need ☐ Early help ☐ Has a child protection plan ☐ Looked after child ☐ None ☐

Social Care Services Involved: Current ☐ Recent past ☐ Past ☐ None ☐

Further details

Is client or partner pregnant: Yes ☐ No ☐ **Due Date:**

Referred to Specialist Midwife Yes ☐ No ☐ Previous ☐

IMMEDIATE RISK IDENTIFIED: Child Care

Prompts: Currently pregnant / Responsible for any child(ren) / Intoxicated while solely responsible for child(ren)
Trigger: Also complete "Childcare & Family support Form" in line with guidance notes if required

Level of risk: No risk ☐ Low ☐ Medium ☐ High ☐

Likelihood of occurrence No risk ☐ Low ☐ Medium ☐ High ☐

ACTION(S):

Safe storage box issued Yes ☐ No ☐ Refused ☐

Childcare & Family Support Form completed Yes ☐ No ☐

Family/Carer Support Offered: Yes ☐ No ☐ Type..

For peer review only - http://bmjopen.bmj.com/site/about/guidelines.xhtml

FAMILY & RELATIONSHIPS

(family health and significant relationships, child protection, care issues, vulnerable adults, support networks)

DOMESTIC VIOLENCE

Have you ever been a victim or perpetrator of domestic violence?

Survivor ☐
Perpetrator ☐
Declined to answer ☐

Current ☐
Recent past ☐
Past ☐
None ☐

For peer review only

IMMEDIATE RISK IDENTIFIED: Domestic Violence

Where DV is identified complete Multi Agency Domestic Violence Risk Identification Form when appropriate

Level of risk:	No risk ☐	Low ☐	Medium ☐	High ☐
Likelihood of occurrence	No risk ☐	Low ☐	Medium ☐	High ☐

ACTION(S):

Black DV card issued ☐ **DASH form completed** ☐ **MARAC referral made** ☐

For peer review only - http://bmjopen.bmj.com/site/about/guidelines.xhtml

CRIMINAL JUSTICE/OFFENDING HISTORY

Current Criminal Justice Status (tick all that apply)

Community Order	☐	DRR	☐	RAR Days	☐
Licence	☐	ATR	☐	Suspended Sentence	☐
Sex Offender Registration	☐	Prolific Offender	☐	ROB	☐
MAPPA	☐	Paying Fines	☐	Schedule 1 Offender	☐

Further details of current criminal justice status:
(length of orders, name of workers involved)

For peer review only

Details of past criminal justice / offending history
(offences committed, length of sentences, targets of violence)

Any record of violent offences?
Further details:

IMMEDIATE RISK IDENTIFIED: Harm to Others

Prompts: Violence to others / Aggression to others / ever used a weapon in assault? / Ever made any preparations to commit harm to others? / Criminal record / Violence/ Domestic Violence/ Aggression towards professionals

Trigger: Where DV is identified complete Multi Agency Domestic Violence Risk Identification Form when appropriate

Level of risk:	No risk ☐	Low ☐	Medium ☐	High ☐
Likelihood of occurrence	No risk ☐	Low ☐	Medium ☐	High ☐

ACTION(S):

For peer review only - http://bmjopen.bmj.com/site/about/guidelines.xhtml

EXISTING RECOVERY CAPITAL

(Including information around housing, financial, education, employment, cultural issues, offending as well as drug & alcohol use)

For peer review only

DVLA / Occupational Concerns / Other

For peer review only - http://bmjopen.bmj.com/site/about/guidelines.xhtml

PERCEPTION OF ONGOING NEEDS & ACTIONS

Signposted / referred	☐ Debt advice	☐ CGL Jigsaw (ExFam)	☐ GP
	☐ Health Shop	☐ Housing Aid	☐ Housing Crisis
	☐ Smoking cessation	☐ Street Outreach	☐ Wellness in Mind
	☐ Other (please state)		

Next appointment date and time: Location: Worker:

Keyworker signature: Date:

Print Name:

DVLA Guidelines for people receiving treatment for Alcohol and/or Drug related difficulties.

You have stated that you hold a current driving licence and continue to drive. As such you have been issued with guidance relating to Alcohol &/or Drug and driving as identified above. Please sign to acknowledge your responsibility to act according to this guidance.

Signed: Date:

Print Name:

For peer review only - http://bmjopen.bmj.com/site/about/guidelines.xhtml

Definitions

The following definitions are relevant to the KLIFAD trial:

Recovery Definition
For the KLIFAD trial we adopted the following definition of "Recovery"

"A period of sustained abstinence from alcohol creating a deeply personal, unique process of change, a way of living a satisfying, hopeful and contributing life even with limitations caused by illness. A process involving the development of new meaning or purpose in one's life which maximises health and wellbeing and participation in the rights, roles and responsibilities of society"[1-4].

Recovery story
A story told by a person about their journey of recovery.

In KLIFAD we are using recovery stories which are primarily first-person lived experience accounts, which include elements of both adversity/struggle and of strength/success/survival related to AUD, and which refer to events or actions over a period. Some stories will include brief fragments presenting clinical perspectives on a case, provided by a clinician who worked with the narrator[5].

Story narrator
The person telling their own recovery story.

Story recipient
The person viewing, reading or listening to someone else's recovery story.

KLIFAD Library
A collection of recovery stories intended for use in the KLIFAD feasibility trial.

Alcohol misuse
The National Institute on Alcohol Abuse and Alcoholism (NIAAA) define alcohol misuse as "alcohol consumption that puts individuals at increased risk for adverse health and social consequences"[6]

Alcohol use disorders
The NIAAA define AUD as "a chronic relapsing brain disorder characterized by an impaired ability to stop or control alcohol use despite adverse social, occupational, or health consequences" [6].

For peer review only - http://bmjopen.bmj.com/site/about/guidelines.xhtml

SP-Focus Group Guide WP1 V2.0

Focus group Guide participants
Work Package 1 (WP1)
Version 2.0 Date:14/10/2020

Study title: **Does knowledge of liver fibrosis affect high-risk drinking behaviour (KLIFAD)? A feasibility randomised controlled trial**

To begin

Welcome to the focus group session. Thanks for taking the time to join us to talk about liver disease screening.

You were invited here today because you attended a liver scan appointment and were given your level of risk for liver disease using a Fibroscan machine. We would like to understand how to provide the best experience for patients undergoing the scan. This includes how the person operating the Fibroscan machine discusses the scan itself and then delivers the results of the scan to patients. We will ask you to read through a script we have prepared to help operators talk through the scan and also a document that provides patients with their results.

Everyone's risk of liver disease may be different. Because everyone has very different life experiences, there are no wrong answers to these questions, but rather different points of view. Please feel free to share your point of view even if it differs from what others have said. Keep in mind that we're just as interested in negative comments as positive comments, and at times the negative comments are the most helpful.

Logistics

- Focus group will last about 2 hours
- Feel free to move around
- Where is the bathroom? Exit?
- Help yourself to refreshments

Ground Rules

- Hope that everyone feels comfortable enough to participate.
- Information provided in the focus group must be kept confidential
- Stay with the group and please don't have side conversations
- Turn off mobile phones if possible
- This is an opportunity to help contribute to the treatment of liver disease!

You've probably noticed the microphone. I'm tape recording the session because I don't want to miss any of your comments. People often say very helpful things in these discussions and I can't write fast enough to get them all down.

For peer review only

If you talk about anyone else during the focus group by name (such as a friend or member of staff) – then we will keep their name anonymous when we write up the results by providing them with a false name. Likewise (the participant) we will also keep your identity anonymous during the write-up by giving you a false name in any reports resulting from this study

Are you okay with this? Do you have any questions?

- Answer any questions they have
- If they do not want to participate, thank them for their time and escort them out of the venue. If they have participated via telephone or over video conferencing – finish the call.

Beginning the focus group

Start recording the interview on the Dictaphone.

Firstly, I want you to think back to your liver scan appointment.

1. Did you understand why you were undergoing a fibroscan and what the scan involved?
2. What was your experience of the scan? Was there anything about the way the operator conducted the scan or talked to you about the scan that you liked/disliked/found helpful?
3. After the scan, what information were you provided with? Including your results, any feedback from the scan operator, and any other information about liver disease?
    a. Was any of this difficult to understand? What information did you find most helpful?
4. Did the scan and/or scan results prompt you to make some changes to improve your liver health?
    a. If you received normal scan results, did you still want to make lifestyle changes?

Now I'd like us to spend the rest of the session today reviewing the documents in front of you. Please take some time to read through these documents and write any thoughts you have about the wording or how the information is presented on the document.

Provide participants with pens
Give participants approximately 10-15 minutes to read through script and fibroscan results

Let's review the operator script. Imagine you were receiving this information from a fibroscan operator.

1. Do you understand the information presented in the script?
    a. What did you like/dislike about the script? What information was helpful/unhelpful? Was anything unclear?
2. Was there any information you felt was missing or that you think would make a useful addition to the script?

For peer review only - http://bmjopen.bmj.com/site/about/guidelines.xhtml

a. Do you have any suggested changes or improvements to the script?

Now let's review the fibroscan result documents. There are three different results a patient can receive, depending on their liver stiffness. Imagine you were receiving this information from a fibroscan operator.

1. Do you think the results made sense for each level of liver disease stiffness?
    a. Did you understand the information? What information was helpful/unhelpful? Was anything unclear?
2. How did the documents make you feel?
    a. Did anyone have a negative reaction/positive reaction?
3. Did you like the way the results were presented (e.g. graphically, visually)?
    a. What would you change? Would you prefer the results to be presented as a value, on a scale, on a graph etc.?
4. Would you feel confident knowing what your result was and how to go about making lifestyle changes from this information?
    a. If not, why and what could we include that would help improve your confidence? Do you think the results documents would need explaining further by the operator?
5. Does anyone have additional thoughts about a specific result document (normal, likely fibrosis, likely cirrhosis)?
    a. Do you think the information reflects the level of risk and need for behaviour change?
6. Is there any other information we should include in the results document?
    a. Do you have any suggested changes or improvements to the results?

Close

Okay, that reaches the end of the questions I wanted to ask today. Is there anything else you wanted to add or talk about that we didn't talk about today?

If you're okay to end the focus group there, I'll switch the Dictaphone off, thank you!

Debriefing

- Thank you for speaking to us.
- Provide participants with a sheet which outlines the range of services etc, go through it with them. If there is any particular service/resource that they have expressed an interest in – then signpost them to it.
    - If they have participated via telephone– a state that they can be sent this via email if this wish or it can be read out to them.
- Thank them again, and ask if they are feeling okay to leave the building/ or hang up/exit the call.

For peer review only

For peer review only - http://bmjopen.bmj.com/site/about/guidelines.xhtml

SP-Change model questionnaire (CMQ) V1.0

Change model questionnaire

Work package 1 (WP1) V1.0 26/10/2020

Study title: Does knowledge of liver fibrosis affect high-risk drinking behaviour (KLIFAD)? A feasibility randomised controlled trial

Your doctor may have asked you to cut down how much alcohol you are drinking. Please find the statement that best describes the way you feel right now about cutting down your alcohol use to the amount the research team recommends

- ☐ I am continuing to drink at the same level and right now I am not considering reducing how much I drink
- ☐ I am continuing to drink at the same level but and right now I am considering reducing how much I drink
- ☐ I am continuing to drink at the same level but I am planning to reduce how much I drink
- ☐ Right now I have reduced how much alcohol I drink, and have maintained this for less than six months
- ☐ Right now I have reduced how much alcohol I drink, and have maintained this for more than six months

For peer review only

Qualitative interview Guide

Work package 3 (WP 3) Feasibility RCT

Study title: **Does knowledge of liver fibrosis affect high-risk drinking behaviour (KLIFAD)? A feasibility randomised controlled trial**

To begin

- Go over the study information again with the participant:
    - Thank you for coming to/agreeing to take part in the interview today…
    - Explain what will happen:
    - 'You'll be asked brief questions about your experience of taking part in the KLIFAD study and some questions about how you felt about taking part in this study and how it might have had an impact on you".
    - There are no 'right' or 'wrong' answers – I am not here to judge you, but to listen to your experiences as everyone's experience is valuable.
    - You can tell us as little or as much information as you want to during this interview, it is kept confidential in the research team. We may use a transcription service, but they are required to sign a confidentiality agreement and identifiers are removed from the typed-up transcript.
    - You can pause or stop the interview at any time if you want a break, you feel uncomfortable or don't want to continue with the interview.
    - After the interview, I will provide you with information about services and resources – that you may find useful if you have any concerns about what you have told us.
- Are you okay with all this? Do you have any questions?
    - Answer any questions they have
    - If they do not want to participate, thank them for their time and escort them out of the venue. If they have participated via telephone or over video conferencing – finish the call.

- Note: We will ask our PPI group about whether to include clarification of specific terms at this point. For example, relapse or lapse or teetotal/sober etc to ensure we ask questions in the participant's preferred way of talking about their alcohol use.
- If you talk about anyone else during the interview by name (such as a friend or member of staff) – then we will keep their name anonymous when we write up the results by providing them with a false name. Likewise (the participant) we will also keep your identity anonymous during the write-up by giving you a false name in any reports resulting from this study
- If you are satisfied with this, please confirm that you still consent to take part.
    - They will have already consented to take part when they signed up. Check you have received this consent (if was by e-mail or post).
    - If unsatisfied and does not want to take part – thank them for their time and guide them out of the venue/end the call.

For peer review only - http://bmjopen.bmj.com/site/about/guidelines.xhtml

Beginning the interview

Start recording the interview on the Dictaphone.

Here we can ask an introductory question to establish some rapport.

Your experience of the KLIFAD study

Q. Have you ever taken part in a research study before?

Q. Can you take me through what you remember about the KLIFAD study? (If they get into specifics of the results…. We'll touch on that later, for now, I'd like you to think about your experience of the scan process as a whole, for example how you felt about the scan or the staff who scanned you.)

Q. Overall, how do you feel about taking part in the KLIFAD study?

Follow up questions: If positive feedback: What did you particularly like?

If negative feedback: What did you not like/thought could be different?

Q. In regard to the fibroscan, did you understand why you were invited to have this scan? Did the staff give you enough information about the scan? Was there anything about the whole process you liked/didn't like?

Q. Where did you watch the stories? Did you watch it with anyone else? What was your response to them?

Your feelings about getting the KLIFAD study

Q. Can you tell me what you remember about your fibroscan scan result?

Follow-up questions: Can you remember the specific value, scale, what the value meant (potential liver disease etc)? Was the result explained clearly, did you understand it? Can you think of ways to improve how we give people their scan results? Is there anything else you think would be helpful to know when you receive your scan result?

For peer review only - http://bmjopen.bmj.com/site/about/guidelines.xhtml

Q. Do you remember how you felt when you first got your fibroscan result? Explore their thoughts and feelings here by using reflection ‘So, I’m hearing that you felt confused and a bit frightened’. Also can use follow-up questions if appropriate e.g., Can you talk a bit more about why you felt scared? Can you describe your feeling of relief? Etc.

Q. What did it feel like watch stories describing other people’s experiences of receiving a fibroscan? Follow up questions: Which stories can you remember accessing? Can you describe any ways in which these made an immediate impact on you? Can you describe any ways in which these have made a longer-tem impact on you? Did you learn anything from the stories?

Q. Did you discuss the KLIFAD study with anyone?

Follow up questions: What part did you talk about? (Scan/story/both?). Who did you talk to about it? How did they feel about it? If they didn’t talk to anyone about it, ask why they didn’t

Q. Now that a bit of time has passed, how do you feel about taking part in the KLIFAD study?

Your use of alcohol since you took part in the KLIFAD study

Q. Can you talk about your use of alcohol at a few different time points? It may be hard to remember this far back so sometimes it’s helpful to look at a calendar and plot out some key dates (e.g. birthdays, trips away) that can help you remember.

1. Your use of alcohol (if any) just before you had your fibroscan result
2. Your use of alcohol (if any) on the day or days after you had your fibroscan result
3. Your use of alcohol (if any) two weeks after you had your result
4. Your use of alcohol (if any) over the last month

Q. Do you think the KLIFAD study changed your use of alcohol in any way?

If yes: explore, how, why do they think it affected it. If no: invite them to talk about that.

Explore if they sought out additional supports e.g. AA

Follow-up: Had you thought about changing before taking part in this study?

Q. If yes to changes, what were your main reasons for making these changes?

Q. If no, tell me more about why you didn’t want to or didn’t feel able to make changes at that time.

For peer review only - http://bmjopen.bmj.com/site/about/guidelines.xhtml

Follow-up questions: Was there anything that helped you make the changes? Was there anything that was a barrier to making changes?

Close

Okay that reaches the end of the questions I wanted to ask you. Is there anything else you wanted to add or talk about that we didn't talk about today?

If you're okay to end the interview there, I'll switch the Dictaphone off, thank you!

Debriefing

- Thank you for speaking to us.
- How are you feeling – is there anything in the interview has troubled you or upset you?
- Provide participant with sheet which outlines range of services etc, go through it with them. If there is any particular service/resource that they have expressed an interest in – then signpost them to it.
    - If they have participated via telephone– state that they can be sent this via email if this wish or it can be read out to them.
- Thank them again, and ask if they are feeling okay to leave the building/ or hang up/exit the call.

For peer review only - http://bmjopen.bmj.com/site/about/guidelines.xhtml

1. Anthony WA. Recovery from mental illness: The guiding vision of the mental health service system in the 1990s. *Psychosocial Rehabilitation Journal.* 1993;16(4):11-23.
2. Rennick-Egglestone S, Morgan K, Llewellyn-Beardsley J, et al. Mental Health Recovery Narratives and Their Impact on Recipients: Systematic Review and Narrative Synthesis. *Canadian journal of psychiatry Revue canadienne de psychiatrie.* 2019;64(10):669-679.
3. Witkiewitz K, Wilson AD, Pearson MR, et al. Profiles of recovery from alcohol use disorder at three years following treatment: can the definition of recovery be extended to include high functioning heavy drinkers? *Addiction (Abingdon, England).* 2019;114(1):69-80.
4. <Policy report - A vision of recovery_ UKDPC recovery consensus group.pdf>.
5. Llewellyn-Beardsley J, Rennick-Egglestone S, Callard F, et al. Characteristics of mental health recovery narratives: Systematic review and narrative synthesis. *PloS one.* 2019;14(3):e0214678.
6. Alcoholism NIoAAa. Helping patients who drink too much: a clinician's guide, updated 2005 edition. Rockville: National Institutes of Health. 2005.

For peer review only

Participant Consent Form
Work Package 3 (WP 3) Feasibility RCT

Version 2.2 Date:24/12/2020

Does knowledge of liver fibrosis affect high-risk drinking behaviour (KLIFAD)? A feasibility randomized controlled trial

Chief Investigator: Professor Stephen Ryder

Please initial each box

1. I confirm that I have read and understood the participant information sheet dated ____________ (version _______) for the above study and have had the opportunity to ask questions.
2. I understand that my participation is voluntary and that I am free to withdraw at any time without my medical care or legal rights being affected.
3. I understand that my medical records may be looked at by authorised individuals from the Sponsor for the study and the UK Regulatory Authority in order to check that the study is being carried out correctly.
4. I understand that should I withdraw from the above study, the data collected from me up to that point will be used in analysing the results of the trial.
5. I consent to the storage, including electronic, of my personal information for this study. I understand that any information that could identify me will be kept strictly confidential and that no personal information will be included in the study report or other publication.
6. I agree that my GP, or if required any other doctor treating me, will be notified of my participation in this study and of my fibroscan results if they shows advance fibrosis
7. I understand I will be offered a voluntary video story recording at end of the study.
8. I consent to access my medical and mental health record via NHS digital services as part of the study
9. I agree to the storage of my anonymized data and that this may be kept for future research papers related to this study and for the completion of the study
10. I agree to take part in the study

For peer review only - http://bmjopen.bmj.com/site/about/guidelines.xhtml

Name of the participant *(Print)* date (dd/mm/yyyy) Participant
s signature

Name of person taking consent *(Print)* date (dd/mm/yyyy) Signature

For peer review only

Participant Consent Form
Qualitative Interview WP3

Version 2.2 Date:24/12/2020

Does knowledge of liver fibrosis affect high-risk drinking behaviour (KLIFAD) A feasibility randomized controlled trial

Chief Investigator: Professor Stephen Ryder

For peer review only

Please <u>initial</u> each box

10. I confirm that I have read and understood the PIS Qualitative interview WP 3dated ____________ (version _______) for the above study and have had the opportunity to ask questions. ☐

11. I understand that my participation is voluntary and that I am free to withdraw at any time without my medical care or legal rights being affected. In addition, should I not wish to answer any question, I am free to decline. ☐

12. I understand that my study and medical records may be looked at by authorised individuals from the Sponsor for the study and the UK Regulatory Authority in order to check that the study is being carried out correctly. ☐

13. I understand that should I withdraw from the above study, the data collected from me up to that point will be used in analysing the results of the trial. ☐

14. consent to the storage, including electronic, of personal information for this study. I understand that any information that could identify me will be kept strictly confidential and that no personal information will be included in the study report or other publication. ☐

15. understand that audio recordings will be used only for analysis and that extracts from the interview, from which I would not be personally identified, may be used in any conference presentation, report or journal article developed as a result of the research. I understand that no other use will be made of the recording without my written permission and that no one outside the research team will be allowed access to the original recording. ☐ ☐

16. understand a NHS approved professional transcription service can be used to transcribe the interveiew audio recoding. ☐

17. understand that if I tell the researcher anything that could cause me or someone else harm, the researcher may have to share this with the relevant healthcare professional. ☐

13

18.
agree to take part in the interview that will be audio recorded (typed up recordings will be anonymised).

___________________________________ ________________ ______________________________

Name of the participant *(Print)* date (dd/mm/yyyy) Participant signature

___________________________________ ______________ ______________________________

Name of person taking consent *(Print)* date (dd/mm/yyyy) Signature

For peer review only

For peer review only - http://bmjopen.bmj.com/site/about/guidelines.xhtml

Participant Consent Form

Work package 1(WP 1) Focus group

Version 2.2 Date:22/12/2020

Does knowledge of liver fibrosis affect high-risk drinking behaviour? (KLIFAD): A feasibility randomized controlled trial

Chief Investigator: Professor Stephen Ryder

For peer review only

Please initial each box

1. I confirm that I have read and understood the participant information sheet Work package 1(WP 1) Focus group dated ____________ (version _______) for the above study and have had the opportunity to ask questions.

2. I understand that my participation is voluntary and that I am free to withdraw at any time and without my medical care or legal rights being affected. In addition, should I not wish to answer any question or questions, I am free to decline.

3. I understand that my study and medical records may be looked at by authorised individuals from the Sponsor for the study and the UK Regulatory Authority in order to check that the study is being carried out correctly.

4. I understand that should I withdraw from the above study, the data collected from me up to that point will be used in analysing the results of the trial.

5. I agree to participate in a Focus group, and I understand that the focus group will be audio recorded. I agree with the audio recording and understand that my responses will be kept strictly confidential.

6. I understand that audio recordings will be used only for analysis and that extracts from the interview, from which I would not be personally identified, may be used in any conference presentation, report or journal article developed as a result of the research. I understand that no other use will be made of the recording without my written permission and that no one outside the research team will be allowed access to the original recording.

7. I understand a NHS approved professional transcription service can be used to transcribe the the focus group audio recoding.

8. I agree to the storage of my anonymized data and that this may be kept for future research papers related to this study and for the completion of the study.

For peer review only - http://bmjopen.bmj.com/site/about/guidelines.xhtml

9. I understand that I will be offered the opportunity of making a voluntary video story recording

10. I agree to take part in the study

Name of the participant *(Print)* date (dd/mm/yyyy) Participant signature

Name of person taking consent *(Print)* date (dd/mm/yyyy) Signature

For peer review only

Participant Consent Form

Work package 1(WP 1) Key Alcohol Worker Focus group

Version 1.0 Date:22/12/2020

Does knowledge of liver fibrosis affect high-risk drinking behaviour? (KLIFAD): A feasibility randomized controlled trial

Chief Investigator: Professor Stephen Ryder

Please initial each box

11. I confirm that I have read and understood the participant information sheet Work package 1(WP 1) Key Alcohol Worker Focus group dated ____________ (version _______) for the above study and have had the opportunity to ask questions.

12. I understand that my participation is voluntary and that I am free to withdraw at any time and without my medical care, legal or employment rights being affected.

13. I understand that should I not wish to answer a question, and I am free to decline.

14. I understand that should I decide to withdraw from the above study, the data collected from me up to that point will be used in analyzing the results of the study

15. I agree to participate in a Focus group, and I understand that the focus group will be audio recorded. I agree with the audio recording and understand that my responses will be kept strictly confidential.

16. I understand that my name will not be linked with the research materials and will not be identified or identifiable in the report or reports that result from the research.

17. I understand that audio recordings will be used only for analysis and that extracts from the interview, from which I would not be personally identified, may be used in any conference presentation, report or journal article developed as a result of the research. I understand that no other use will be made of the recording without my written permission and that no one outside the research team will be allowed access to the original recording.

18. I agree to the storage of my anonymized data and that this may be kept for future research papers related to this study and for the completion of the study.

19. I agree to take part in the study

For peer review only - http://bmjopen.bmj.com/site/about/guidelines.xhtml

Name of the participant *(Print)* date (dd/mm/yyyy) Participant signature

Name of person taking consent *(Print)* date (dd/mm/yyyy) Signature

For peer review only

For peer review only - http://bmjopen.bmj.com/site/about/guidelines.xhtml

Participant Consent Form
Work package 2 (WP 2)Video recording

Version 2.2 Date:22/12/2020

Does knowledge of liver fibrosis affect high-risk drinking behaviour (KLIFAD)? A feasibility randomized controlled trial

Chief Investigator: Professor Stephen Ryder

Please initial each box

1. I confirm that I have read and understood the participant information sheet Work package 2 (WP 2) Video recording dated ____________ (version _______) for the above study and have had the opportunity to ask questions. ☐

2. I understand that my participation is voluntary and that I am free to withdraw at any time without my medical care or legal rights being affected. In addition, should I not wish to answer any particular question or questions, I am free to decline. ☐

3. I understand that my study and medical records may be looked at by authorised individuals from the Sponsor for the study and the UK Regulatory Authority in order to check that the study is being carried out correctly. ☐

4. I understand that should I withdraw from the above study, the data collected from me up to that point will be used in analysing the results of the trial . ☐

5. I understand that any information that could identify me will be kept strictly confidential Only anonymised information will be used for analysis for this study and may be used in any conference presentation, report or journal article developed as a result of the research from which I would not be personally identified, I understand that no other use will be made of the recording without my written permission. ☐

6. I understand that my identity cannot be hidden in the video recording and that there is a risk of my story becoming openly accessible to other people. ☐

7. I agree to the storage of my anonymized data and that this may be kept for future research papers related to this study and for the completion of the study ☐

8. I consent to participate in recovery video recording. ☐

9. I agree to take part in the study ☐

For peer review only - http://bmjopen.bmj.com/site/about/guidelines.xhtml

Name of participant *(Print)* date (dd/mm/yyyy) Participant signature

Name of person taking consent *(Print)* date (dd/mm/yyyy) Signature

For peer review only - http://bmjopen.bmj.com/site/about/guidelines.xhtml

Reporting checklist for protocol of a clinical trial.

Based on the SPIRIT guidelines.

Instructions to authors

Complete this checklist by entering the page numbers from your manuscript where readers will find each of the items listed below.

Your article may not currently address all the items on the checklist. Please modify your text to include the missing information. If you are certain that an item does not apply, please write "n/a" and provide a short explanation.

Upload your completed checklist as an extra file when you submit to a journal.

In your methods section, say that you used the SPIRITreporting guidelines, and cite them as:

Chan A-W, Tetzlaff JM, Gøtzsche PC, Altman DG, Mann H, Berlin J, Dickersin K, Hróbjartsson A, Schulz KF, Parulekar WR, Krleža-Jerić K, Laupacis A, Moher D. SPIRIT 2013 Explanation and Elaboration: Guidance for protocols of clinical trials. BMJ. 2013;346:e7586

		Reporting Item	Page Number
**Administrative information**			
Title	#1	Descriptive title identifying the study design, population, interventions, and, if applicable, trial acronym	1
Trial registration	#2a	Trial identifier and registry name. If not yet registered, name of intended registry	1
Trial registration: data set	#2b	All items from the World Health Organization Trial Registration Data Set	1
Protocol version	#3	Date and version identifier	1
Funding	#4	Sources and types of financial, material, and other support	1
Roles and responsibilities: contributorship	#5a	Names, affiliations, and roles of protocol contributors	1, 16

For peer review only

Roles and responsibilities: sponsor contact information	#5b	Name and contact information for the trial sponsor	1
Roles and responsibilities: sponsor and funder	#5c	Role of study sponsor and funders, if any, in study design; collection, management, analysis, and interpretation of data; writing of the report; and the decision to submit the report for publication, including whether they will have ultimate authority over any of these activities	11.12
Roles and responsibilities: committees	#5d	Composition, roles, and responsibilities of the coordinating centre, steering committee, endpoint adjudication committee, data management team, and other individuals or groups overseeing the trial, if applicable (see Item 21a for data monitoring committee)	11,12
**Introduction**			
Background and rationale	#6a	Description of research question and justification for undertaking the trial, including summary of relevant studies (published and unpublished) examining benefits and harms for each intervention	5
Background and rationale: choice of comparators	#6b	Explanation for choice of comparators	5,6
Objectives	#7	Specific objectives or hypotheses	6
Trial design	#8	Description of trial design including type of trial (eg, parallel group, crossover, factorial, single group), allocation ratio, and framework (eg, superiority, equivalence, non-inferiority, exploratory)	6
**Methods: Participants, interventions, and outcomes**			
Study setting	#9	Description of study settings (eg, community clinic, academic hospital) and list of countries where data will be collected. Reference to where list of study sites can be obtained	6
Eligibility criteria	#10	Inclusion and exclusion criteria for participants. If applicable, eligibility criteria for study centres and individuals who will	Table 1

For peer review only

		perform the interventions (eg, surgeons, psychotherapists)	
Interventions: description	#11a	Interventions for each group with sufficient detail to allow replication, including how and when they will be administered	6,7,8
Interventions: modifications	#11b	Criteria for discontinuing or modifying allocated interventions for a given trial participant (eg, drug dose change in response to harms, participant request, or improving / worsening disease)	N/A
Interventions: adherance	#11c	Strategies to improve adherence to intervention protocols, and any procedures for monitoring adherence (eg, drug tablet return; laboratory tests)	11,12
Interventions: concomitant care	#11d	Relevant concomitant care and interventions that are permitted or prohibited during the trial	N/A
Outcomes	#12	Primary, secondary, and other outcomes, including the specific measurement variable (eg, systolic blood pressure), analysis metric (eg, change from baseline, final value, time to event), method of aggregation (eg, median, proportion), and time point for each outcome. Explanation of the clinical relevance of chosen efficacy and harm outcomes is strongly recommended	10, 11
Participant timeline	#13	Time schedule of enrolment, interventions (including any run-ins and washouts), assessments, and visits for participants. A schematic diagram is highly recommended (see Figure)	9
Sample size	#14	Estimated number of participants needed to achieve study objectives and how it was determined, including clinical and statistical assumptions supporting any sample size calculations	9
Recruitment	#15	Strategies for achieving adequate participant enrolment to reach target sample size	6,7,8,9
**Methods: Assignment of interventions (for controlled trials)**			
Allocation: sequence generation	#16a	Method of generating the allocation sequence (eg, computer-generated random numbers), and list of any factors for stratification. To reduce predictability of a random sequence, details of any planned restriction (eg, blocking) should be provided in a separate document that is unavailable to those who enrol participants or assign interventions	9

For peer review only

Allocation concealment mechanism	#16b	Mechanism of implementing the allocation sequence (eg, central telephone; sequentially numbered, opaque, sealed envelopes), describing any steps to conceal the sequence until interventions are assigned	9
Allocation: implementation	#16c	Who will generate the allocation sequence, who will enrol participants, and who will assign participants to interventions	9
Blinding (masking)	#17a	Who will be blinded after assignment to interventions (eg, trial participants, care providers, outcome assessors, data analysts), and how	N/A
Blinding (masking): emergency unblinding	#17b	If blinded, circumstances under which unblinding is permissible, and procedure for revealing a participant's allocated intervention during the trial	N/A
**Methods: Data collection, management, and analysis**			
Data collection plan	#18a	Plans for assessment and collection of outcome, baseline, and other trial data, including any related processes to promote data quality (eg, duplicate measurements, training of assessors) and a description of study instruments (eg, questionnaires, laboratory tests) along with their reliability and validity, if known. Reference to where data collection forms can be found, if not in the protocol	9
Data collection plan: retention	#18b	Plans to promote participant retention and complete follow-up, including list of any outcome data to be collected for participants who discontinue or deviate from intervention protocols	9,10,11
Data management	#19	Plans for data entry, coding, security, and storage, including any related processes to promote data quality (eg, double data entry; range checks for data values). Reference to where details of data management procedures can be found, if not in the protocol	11,12,13
Statistics: outcomes	#20a	Statistical methods for analysing primary and secondary outcomes. Reference to where other details of the statistical analysis plan can be found, if not in the protocol	11
Statistics: additional analyses	#20b	Methods for any additional analyses (eg, subgroup and adjusted analyses)	11

For peer review only

Statistics: analysis population and missing data	#20c	Definition of analysis population relating to protocol non-adherence (eg, as randomised analysis), and any statistical methods to handle missing data (eg, multiple imputation)	11
**Methods: Monitoring**			
Data monitoring: formal committee	#21a	Composition of data monitoring committee (DMC); summary of its role and reporting structure; statement of whether it is independent from the sponsor and competing interests; and reference to where further details about its charter can be found, if not in the protocol. Alternatively, an explanation of why a DMC is not needed	12,13
Data monitoring: interim analysis	#21b	Description of any interim analyses and stopping guidelines, including who will have access to these interim results and make the final decision to terminate the trial	12,13
Harms	#22	Plans for collecting, assessing, reporting, and managing solicited and spontaneously reported adverse events and other unintended effects of trial interventions or trial conduct	12
Auditing	#23	Frequency and procedures for auditing trial conduct, if any, and whether the process will be independent from investigators and the sponsor	11,12,13
**Ethics and dissemination**			
Research ethics approval	#24	Plans for seeking research ethics committee / institutional review board (REC / IRB) approval	1
Protocol amendments	#25	Plans for communicating important protocol modifications (eg, changes to eligibility criteria, outcomes, analyses) to relevant parties (eg, investigators, REC / IRBs, trial participants, trial registries, journals, regulators)	11,12,13
Consent or assent	#26a	Who will obtain informed consent or assent from potential trial participants or authorised surrogates, and how (see Item 32)	11,12,13
Consent or assent: ancillary studies	#26b	Additional consent provisions for collection and use of participant data and biological specimens in ancillary studies, if applicable	N/A
Confidentiality	#27	How personal information about potential and enrolled participants will be collected, shared, and maintained in order to protect	11,12,13

For peer review only

		confidentiality before, during, and after the trial	
Declaration of interests	#28	Financial and other competing interests for principal investigators for the overall trial and each study site	16
Data access	#29	Statement of who will have access to the final trial dataset, and disclosure of contractual agreements that limit such access for investigators	16
Ancillary and post trial care	#30	Provisions, if any, for ancillary and post-trial care, and for compensation to those who suffer harm from trial participation	13
Dissemination policy: trial results	#31a	Plans for investigators and sponsor to communicate trial results to participants, healthcare professionals, the public, and other relevant groups (eg, via publication, reporting in results databases, or other data sharing arrangements), including any publication restrictions	13
Dissemination policy: authorship	#31b	Authorship eligibility guidelines and any intended use of professional writers	13
Dissemination policy: reproducible research	#31c	Plans, if any, for granting public access to the full protocol, participant-level dataset, and statistical code	13
**Appendices**			
Informed consent materials	#32	Model consent form and other related documentation given to participants and authorised surrogates	11-20
Biological specimens	#33	Plans for collection, laboratory evaluation, and storage of biological specimens for genetic or molecular analysis in the current trial and for future use in ancillary studies, if applicable	N/A

The SPIRIT Explanation and Elaboration paper is distributed under the terms of the Creative Commons Attribution License CC-BY-NC. This checklist was completed on 28. June 2021 using https://www.goodreports.org/, a tool made by the EQUATOR Network in collaboration with Penelope.ai

For peer review only

BMJ Open

Does knowledge of liver fibrosis affect high-risk drinking behaviour (KLIFAD)?: Protocol for a feasibility randomised controlled trial

Journal:	*BMJ Open*
Manuscript ID	bmjopen-2021-054954.R2
Article Type:	Protocol
Date Submitted by the Author:	18-Oct-2021
Complete List of Authors:	Subhani, Mohsan; University of Nottingham, Nottingham Digestive Diseases Biomedical Research Centre (NDDC); Nottingham University Hospitals NHS Trust, NIHR Nottingham Biomedical Research Centre<br>Jones, Katy; University of Nottingham School of Medicine, Division of Psychiatry & Applied Psychology<br>Sprange, Kirsty; University of Nottingham, Nottingham Clinical Trials Research Unit<br>Rennick-Egglestone, Stefan; University of Nottingham School of Health Sciences,<br>Knight, Holly; University of Nottingham,<br>Morling, Joanne; University of Nottingham<br>Enki, Doyo; University of Nottingham, Medical Statistics<br>Wragg, Andrew; Queen's Medical Centre Nottingham University Hospital NHS Trust<br>Ryder, Stephen; Nottingham University Hospitals NHS Trust, Nottingham Digestive Diseases Biomedical Research Centre (NDDC), School of Medicine
**Primary Subject Heading**:	Gastroenterology and hepatology
Secondary Subject Heading:	Addiction, Evidence based practice, Health economics, Public health
Keywords:	Hepatology < INTERNAL MEDICINE, Gastroenterology < INTERNAL MEDICINE, PUBLIC HEALTH

SCHOLARONE™
Manuscripts

I, the Submitting Author has the right to grant and does grant on behalf of all authors of the Work (as defined in the below author licence), an exclusive licence and/or a non-exclusive licence for contributions from authors who are: i) UK Crown employees; ii) where BMJ has agreed a CC-BY licence shall apply, and/or iii) in accordance with the terms applicable for US Federal Government officers or employees acting as part of their official duties; on a worldwide, perpetual, irrevocable, royalty-free basis to BMJ Publishing Group Ltd ("BMJ") its licensees and where the relevant Journal is co-owned by BMJ to the co-owners of the Journal, to publish the Work in this journal and any other BMJ products and to exploit all rights, as set out in our licence.

The Submitting Author accepts and understands that any supply made under these terms is made by BMJ to the Submitting Author unless you are acting as an employee on behalf of your employer or a postgraduate student of an affiliated institution which is paying any applicable article publishing charge ("APC") for Open Access articles. Where the Submitting Author wishes to make the Work available on an Open Access basis (and intends to pay the relevant APC), the terms of reuse of such Open Access shall be governed by a Creative Commons licence – details of these licences and which Creative Commons licence will apply to this Work are set out in our licence referred to above.

Other than as permitted in any relevant BMJ Author's Self Archiving Policies, I confirm this Work has not been accepted for publication elsewhere, is not being considered for publication elsewhere and does not duplicate material already published. I confirm all authors consent to publication of this Work and authorise the granting of this licence.

For peer review only

Does knowledge of liver fibrosis affect high-risk drinking behaviour (KLIFAD)?: Protocol for a feasibility randomised controlled trial

Mohsan Subhani[1,2], Katy A Jones[3], Kirsty Sprange[4], Stefan Rennick-Egglestone[5], Holly Knight[6], Joanne R Morling[1,2,6], Doyo G Enki[7], Andrew Wragg[2], Stephen D Ryder[1,2]

Affiliations

[1]Nottingham Digestive Diseases Biomedical Research Centre (NDDC), School of Medicine, University of Nottingham

[2]NIHR Nottingham Biomedical Research Centre, Nottingham University Hospitals NHS Trust and the University of Nottingham, Nottingham, UK

[3]Academic Unit of Mental Health and Clinical Neuroscience, School of Medicine, C24 Institute of Mental Health, University of Nottingham

[4]Nottingham Clinical Trials Unit, University of Nottingham

[5]School of Health Sciences, Institute of Mental Health, University of Nottingham

[6]Division of Epidemiology and Public Health, University of Nottingham, Nottingham, UK

[7]School of Medicine, University of Nottingham

Acronym

KLIFAD (**K**nowledge of **LI**ver **F**ibrosis **A**ffects **D**rinking)

Corresponding Author

Mohsan Subhani

Email: mohsan.subhani@nottingham.ac.uk Telephone: 0044 7572612429

Research reference numbers

IRAS Number:	273765
SPONSOR Number:	124481/2020
FUNDER Number:	RfPB NIHR201146
ISRCTN	16922410
Protocol version	V1.4

Sponsor

Nottinghamshire Healthcare NHS Foundation Trust

research@nottshc.nhs.uk

Funder

National Institute for Health Research

Scheme: Research for Patient Benefit (RfPB)

For peer review only

Abbreviations

ARLD	Alcohol-Related Liver Disease
ARVS	Alcohol Recovery Video Stories
AUD	Alcohol Use Disorders
AUDIT	Alcohol Use Disorder Identification Test
BRC	Biomedical Research Centre
CONSORT	Consolidated Standards of Reporting Trials
COREQ	Consolidated Criteria for Reporting Qualitative Studies
GCP	Good Clinical Practice
kPa	Kilopascal
NDTMS	National Drug Treatment Monitoring System
NDDC	Nottingham Digestive Diseases Centre
NHS	National Health Service
NICE	National Institute of Clinical Excellence
NRN	Nottingham Recovery Network
NUH	Nottingham University Hospital
PIS	Patient Information Sheet
PPI	Patient and Public Involvement
RCT	Randomised Controlled Trial
REC	Research Ethics Committee
SADQ	Severity of Alcohol Dependence Questionnaire
UK	United Kingdom
WoSRES	West of Scotland Research Ethics Service
WP	Work Package

For peer review only

Abstract

Introduction

Heavy drinkers in contact with alcohol services do not routinely have access to testing to establish the severity of potential liver disease. Transient elastography by FibroScan can provide this information. A recent systematic review suggested providing feedback to patients based on markers of liver injury can be an effective way to reduce harmful alcohol intake. This randomised control trial aims to establish the feasibility of conducting a larger national trial to test the effectiveness of FibroScan advice and alcohol recovery video stories in changing high-risk drinking behaviour in community alcohol services common to United Kingdom practice.

Methods and analysis

This feasibility trial consists of three work packages (WP). **WP1:** To draft a standardised script for FibroScan operators to deliver liver disease-specific advice to eligible participants having FibroScan. **WP2:** To create a video library of alcohol recovery video stories for use in the feasibility RCT (WP3). **WP3:** To test the feasibility of the trial design, including the FibroScan script and video stories developed in WP1 and WP2 in a one-to-one individual randomised trial in community alcohol services. Semi-structured interviews will be conducted at six months follow up for qualitative evaluation. Outcomes will be measures of the feasibility of conducting a larger RCT related to participant recruitment and follow-up, intervention delivery, including the use of the KLIFAD FibroScan scripts and videos, clinical outcomes and the acceptability and experience of the intervention and trial-related procedures. Data analysis will primarily be descriptive to address the feasibility aims of the trial. All proposed analyses will be documented in a Statistical Analysis Plan.

Ethics and dissemination

This trial received favourable ethical approval from the West of Scotland Research Ethics Service (WoSRES) on 20th January 2021, REC reference: 20/WS/0179. Results will be submitted for publication to a peer-reviewed journal.

Trial registration number

ISRCTN16922410, Pre-results

Keywords: Alcohol. FibroScan. Alcohol-related liver disease. Alcohol recovery stories.

For peer review only

Strengths and limitations of the trial

- The KLIFAD trial is the first randomised control trial to evaluate the feasibility of using non-invasive liver stiffness measurement and alcohol recovery video stories as a behavioural intervention.
- The mixed-methods design of the KLIFAD trial will enable us to test the acceptability of trial-specific procedures to participants and key alcohol workers.
- The KLIFAD trial will enable a definitive trial to establish the effectiveness of non-invasive screening for liver fibrosis in community alcohol services.
- The primary limitation of the KLIFAD trial is that it is a single centre trial which could limit the generalisation of findings.
- Due to the nature of the KLIFAD intervention, blinding is not possible.

For peer review only

Introduction

Alcohol-related liver disease (ARLD) is the most common cause of cirrhosis in the United Kingdom (UK), and mortality from ARLD has risen significantly over the past three decades. It is now the second most common cause of working life years lost in men and fifth in women[1,2]. Europe has one of the highest prevalence of Alcohol Use Disorders (AUD), involving 15% of men and 3.5% of women[2]. Around 25% of the UK population drink above the UK recommended level of 14 units per week, and 10% are harmful drinkers[3]. The UK's total per capita pure alcohol intake for people age ≥15 years is 11.4 litres/annum per person, which is twice the global average of 6.4 litres/annum per person [2,3]. Approximately 20-30% of lifelong drinkers develop liver cirrhosis, and the risk is even higher (35%) among harmful drinkers [4,5].

ARLD causes no symptoms in its earlier stages; indeed, patients are often unaware they have serious physical health problems until they present with the complications of cirrhosis, for example; ascites, jaundice, encephalopathy, variceal bleed, and liver failure, when the opportunity for treatment and recovery of liver health are significantly reduced [1,5,6]. It is estimated that the cost to the UK of alcohol on health is £3.5 billion per year [3,7], consuming 3.6% of the National Health Service (NHS) annual budget [8]. In England, there were 5,698 alcohol-specific deaths in 2018, the alcohol-specific age-standardised death rate was 11.9/100,000 (male=16.4 female=7.6). Nottingham (UK) has one of the highest (total=18.6, male=26.8, female 10.2) alcohol-specific age-standardised death rate/100,000 in the country [9]. A recent trial from the United States (US) predicted a 75% increase in age-standardised annual mortality and a 65% increase in decompensated cirrhosis due to ARLD over the next two decades[10].

Systematic reviews of Randomised Controlled Trials (RCTs) have established that delivering brief advice about alcohol to harmful drinkers helps them reduce their alcohol consumption[11,12]. Most studies were conducted in primary care settings where the prevalence of liver disease is likely to be markedly lower than in specialist alcohol treatment services. In alcohol services, where high levels of physical and psychological dependence on alcohol are frequent, National Institute of Clinical Excellence (NICE) guidelines state adults with high levels of alcohol dependency should be assessed and offered intensive structured community-based interventions (with or without medical therapy) as these provide the best chance of achieving and maintaining abstinence from alcohol[13]. Most clinical services in the UK are based on these principles. Individual programmes vary by locality with many of these services delivered by non-NHS providers. Despite the delivery of brief advice and other alcohol-related interventions in clinical practice for over two decades, mortality and morbidity due to alcohol misuse continues to rise in the UK[3]. There is a pressing need to optimise existing interventions to reduce harmful alcohol intake and examine effective alternative options.

Early diagnosis of liver fibrosis provides an opportunity to intervene and reduce or stop alcohol intake. This is known to be the most effective way of preventing liver disease progression [14]. Transient elastography by FibroScan (Echosens, France) has been used in primary care (General Practice) settings to detect liver disease in populations identified as having liver disease risk (heavy drinkers and those with type 2 diabetes). These studies showed that screening asymptomatic individuals based on risk for liver disease doubles the rates of liver cirrhosis diagnosis in the primary care populations studied[15,16]. Moreover, a recent systematic review suggested providing feedback to patients based on markers of liver injury can be an effective way to reduce harmful alcohol intake[17]. Access to recovery stories can help address mental health problems and support recovery from addiction[18,19]. Peer support from people

For peer review only - http://bmjopen.bmj.com/site/about/guidelines.xhtml

who have recovered from alcohol misuse is beneficial in modifying high risk drinking behaviour[20].

This trial aims to investigate the feasibility and acceptability of conducting an RCT in community specialist alcohol services settings run by Nottingham Recovery Network (NRN) and to test the acceptability of trial interventions (FibroScan and Alcohol Recovery Video Stories, ARVS).

Selection of the term "alcohol misuse"

We acknowledge the heterogeneity in language used to describe alcohol use, and also the stigma associated with some commonly used terms, which itself can act as barrier to change[21]. Some terms, such as alcohol use disorder (AUD), are not well understood in the general population. The concept for this feasibility trial was developed in collaboration with Patient and Public Involvement (PPI) groups. After extensive discussion between the study team and PPI groups, we have opted for the term **'alcohol misuse'** as a general term to cover excess alcohol intake, harmful alcohol intake, drinking problems, alcohol dependence, and AUD.

We define alcohol misuse as "weekly alcohol intake ≥14 units, or an AUDIT score of ≥ 8, or key alcohol worker and/or physician diagnosis, or referral from any other services with problem drinking".

The other definitions relevant to KLIFAD trial are provided in supplementary material (SP-Definitions)

Methods and analysis:

KLIFAD (**K**nowledge of **LI**ver **F**ibrosis **A**ffects **D**rinking) is a parallel design feasibility RCT. The trial will be conducted in a single centre in the UK, carried out at three community alcohol services in Nottingham (the Wellbeing Hub, Edwin House and the Primary Care Alcohol Clinic run by the Nottingham Recovery Network) hosted by Framework and Nottingham Recovery Network (NRN) and working in partnership with Nottinghamshire NHS Foundation Trust.

The KLIFAD trial consists of three work packages (WP) (Figure 1).

Work Package one (WP1)

WP1 aims to design a standardised script framework for FibroScan operators to deliver liver disease-specific advice to participants having FibroScan as part of the feasibility RCT (WP3).

FibroScan, is an ultrasound technology developed by Echosence, France, which non-invasively asseses liver stiffness. A prototype script for FibroScan has been created in consultation with the existing KLIFAD Patient Public Involvement (PPI) group covering three ranges of FibroScan scores, normal ≤7 Kilopascal (kPa), intermediate fibrosis 8-15 kPa and advance fibrosis ≥15 kPa. A sample script is provided in supplementary material (SP) and the trial flow chart in Figure 2.

We will organise separate participant and FibroScan operator focus groups to collect feedback on the prototype scripts. The participant focus group will allow us to investigate the key messages to be included in the script and feedback, as well as considering how best to present the FibroScan results (e.g., graphically, in the text). The FibroScan operator focus group will specifically discuss implementation in clinical practice. In addition, to evaluate the stage of change that each participant has reached, a validated readiness to change model will be piloted[22].

Following Krueger's (1988) focus group guide, each focus group will include five to eight participants and will last for a maximum of two hours[23]. Depending upon the latest Covid-19

For peer review only - http://bmjopen.bmj.com/site/about/guidelines.xhtml

guidelines the focus group will be either virtual or face-to-face. A topic guide will be used (SP-Focus Group Guide WP1 V2.0). We aim to arrange two participant focus groups and one FibroScan operator focus group. The focus groups will be facilitated by two members of the research team. Examples of questions include:

a) If you were a participant in the trial, would the script make sense to you?
b) Are there any parts of the script that you do not understand, and if so, why?
c) What is the best way to present the results of the FibroScan (e.g., graphically, in the text)?

Eligible participants (Table 1) will be identified and recruited through multiple channels. For example, via existing patient forums at all three recruitment settings, the KLIFAD PPI group, by offering information to patients self-presenting to any of the trial treatment settings, snowball methods, and via Black, Asian and minority ethnicity/Framework PPI groups. The focus group meeting will be recorded and transcribed verbatim either by automated software or an independent sponsor-approved transcriber. After the first participant focus group the FibroScan script will be edited considering feedback and a second focus group will then be held to review iterated scripts. The final scripts will be sent via email to participants of focus groups for any final thoughts. We will then organise a FibroScan operator focus group of key alcohol workers working at any of the recruitment settings who are willing to give informed consent, to discuss any specific implementation issues.

After the focus groups, we will collect participant feedback on the change model (SP-Change model questionnaire (CMQ) V1.0) to get an initial sense of the applicability of readiness to change following discussion about the scripts.

**Work package one**	
**Inclusion criteria**	**Exclusion criteria**
A person age ≥18 years	Other primary substance misuse even where alcohol is a factor
Primary problem of alcohol misuse[a]	Lacks capacity to confirm consent
Had FibroScan in past	
**Work package two**	
**Inclusion criteria**	**Exclusion criteria**
A person age ≥18 years	Lacks capacity to confirm consent
Primary problem of alcohol misuse	
Had FibroScan in past	
A person with lived experience of alcohol problems	
A person willing to consent to the recording and public use of their video recording	

For peer review only - http://bmjopen.bmj.com/site/about/guidelines.xhtml

Work package three the randomisation phase	
**Inclusion criteria**	**Exclusion criteria**
A person age ≥18 years	Other primary substance misuse even where alcohol is a factor
Primary problem of alcohol misuse	Lacks capacity to confirm consent
	Referrals from driving offences and student referrals[b]
	Out of area clients at Edwin House[c]
	Participants unable to comply with trial procedures

Table 1: KLIFAD trial eligibility criteria

[a]Alcohol misuse was defined as, weekly alcohol intake ≥14 units, or an AUDIT score of ≥ 8, or key alcohol worker and/or physician diagnosis, or referral from any other services with problem drinking.

[b]As these individuals are essentially not self-presenting, may have different motivation and have lower overall levels of alcohol use and so are substantially lower risk of having liver disease.

[c]In whom we cannot obtain follow up data due to lack of follow up availability.

For peer review only

Work Package Two (WP2)

WP2 aims to create a video library of ARVS from people with a history of alcohol misuse. These ARVS will be used in the feasibility RCT (WP3).

Receiving mental health recovery stories can provide benefits to some people experiencing mental health distress [18,24,25], and the effectiveness of mental health recovery stories as an intervention to increase quality of life has been examined in a clinical trial[26]. However, equivalent evidence is not available for the impact of ARVS. So that we can explore the impact of stories of recovery from alcohol misuse, in WP2 we will develop a set of recovery stories from participants who have successfully overcome their alcohol misuse. These videos will be peer-reviewed by the KLIFAD PPI group which will include input from Nottingham University Hospitals NHS Trust (NUH) Black, Asian and minority Ethnic patient and public involvement Group. Based on feedback the videos will then be edited ready for use in the feasibility RCT (WP3). All edits will be agreed upon with the story narrators.

For each narrator, we will follow their preference to create either:

- A recovery story that starts with an open-ended question where narrators have the liberty to tell their story without interruption *or*
- A recovery story in which the participant is asked a set of standard questions.

Drinking history and last FibroScan reading will be included at the start of each video. Eligible participants (Table 1) will be recruited through the channels used in WP1. Those who took part in WP1 will also be invited to take part in WP2. A purposive sample based on demographic and liver disease severity of six to nine individuals will be selected[27]. We will arrange a meeting with the KLIFAD PPI group to discuss what makes a video impactful. The outline of WP2 is given in Figure 2.

For peer review only - http://bmjopen.bmj.com/site/about/guidelines.xhtml

The ARVS will be recorded either at NDDC Biomedical Research Centre Nottingham University Hospital, the University of Nottingham, or the participant's usual place of residence. Each video will be of two-to-five-minute duration. Videos will be titled based on FibroScan score (low-risk, medium and high-risk score). Videos will be subtitled and depending on the final video format, after the feedback, we envisage adding a photograph of the storyteller and a short-associated text on the title page with informed consent from the participant. The video stories will be brought together in a single tablet computer-based package from which the participant will be able to choose their most preferred video after receiving a FibroScan score. Collaborative work between a clinician and patient can make a significant impact on the recovery process [28] and hence in some videos, and with consent by narrators, we will include sections of a video narrated by a clinician the narrator has worked with.

All video stories recorded as part of the KLIFAD trial will have peer review by the trial team and KLIFAD/Black, Asian and ethnic minority PPI groups. The videos will be shown in the same format that they would be used in WP3.

Work Package 3 (WP3)- Feasibility RCT

A feasibility RCT of parallel groups (one-to-one) will compare usual care (assessment and entry into an alcohol reduction programme which does not include information on liver disease severity) to usual care plus feedback from the FibroScan and ARVS. The eligibility for WP3 is provided in Table 1 and the attached flow chart (Figure 3).

Objectives

Bowen et al (2009)'s guide for feasibility studies was used to decide objectives [29].

1. **Test:** the intervention (FibroScan plus feedback and ARVS) in a feasibility randomised control trial.
2. **Acceptability**: of feasibility randomised control trial related procedures and interventions among patients and healthcare workers.
3. **Feasibility outcomes**: to establish recruitment rate, consent rate, dropout rate, and completion rate for accurate sample size calculation for future large-scale RCT.
4. **Refine**: the eligibility and randomisation criteria for a future large-scale RCT.
5. **Implementation and practicality:** to assess the ability of community alcohol services to deliver the intervention, and training and support needs for community alcohol services keyworkers for delivering the intervention.
6. **Adaptation:** of KLIFAD trial interventions, FibroScan feedback, and ARVS format and access as per suggestions from participants and key alcohol workers
7. **Limited efficacy**: to test limited efficacy of KLIFAD interventions

Intervention Group

Participants randomised to the intervention arm will receive a FibroScan, feedback on FibroScan results and watch ARVS immediately after. The ARVS will be made available should a participant wish to watch them later.

Control group

Participants randomised to the control arm will continue with standard treatment (usual care) provided at the three treatment settings. The participants in this arm will be offered FibroScan at 6 months.

For peer review only - http://bmjopen.bmj.com/site/about/guidelines.xhtml

As part of standard treatment, the recruitment settings provide different types of interventions to participants in line with the National Drug Treatment Monitoring System Dataset (NDTMS) and Public health England (PHE) guidelines [30]. Existing treatment programmes can run for up to 12-weeks.

For adult drug and alcohol services there are three main categories of standard intervention (usual care) delivered by the NRN:

a) Psychological: which includes motivational interventions, family and social network interventions, and cognitive and behavioural based relapse prevention interventions (substance misuse specific).
b) Recovery Support: which includes 12 step work and counselling.
c) Pharmacological: which involves prescribing medication for drug and/or alcohol relapse prevention support. For example, naltrexone, acamprosate, disulfiram as part of alcohol or opioid relapse prevention therapy and Chlordiazepoxide for acute alcohol withdrawal.

Specific treatment programmes are started after an initial assessment and based on the participant's needs. The duration of contact with services varies, most participants stay with services for 12 weeks, some get discharged early, and a few stay longer than six months.

Methods

Sample size

As this is a feasibility trial, a formal sample size calculation for between-group comparisons of a primary outcome is not appropriate. Researchers have previously recommended sample sizes between 24-50 to satisfactorily achieve feasibility outcomes [31-33].

After discussion with community alcohol services data manager and considering variation in number of patients presenting per week, we aim to approach 40 eligible participants per month. Assuming a 50% consent rate we anticipate randomising 20 participants per month (10 per month per arm) for a recruitment period of six months. With an estimated sample size of 120, we will be able to calculate a dropout rate of 80% within a 95% confidence interval of +/-7.1%. Assuming a non-differential follow-rate of 80%, this target sample size should provide follow-up outcome data on a minimum of 48 participants in each of the two arms.

Randomisation

The participants will be individually allocated on a one-to-one ratio using minimisation with a probabilistic element. The minimisation variables will be age, gender, ethnicity, and severity of alcohol misuse based on the Severity of Alcohol Dependence Questionnaire (SADQ) score. To minimise selection bias the randomisation will be externally performed by a data manager from Nottingham Recovery Network.

Schedule of visits

Baseline

The baseline visit will be on the day when the participant starts standard treatment at any recruitment setting. At this visit written informed consent will be given by participants and participants will be randomised to the intervention or control group. Participants in both arms will have an initial detailed assessment (SP-NRN assessment form Supplementary Material) as part of their standard care. This includes the collection of baseline demographic and clinical data (e.g., age, gender, ethnicity). Participants randomised to the control arm will continue

For peer review only - http://bmjopen.bmj.com/site/about/guidelines.xhtml

with usual care while participants randomised to the intervention arm will have the usual care and FibroScan followed by standardised script feedback with ARVS watched immediately after the FibroScan result.

Three months

This visit will be part of usual care, no research specific activity will be carried out. The research data will be extracted from routinely collected data from three treatment settings.

Six months

This will be a telephone consultation or in-person appointment by the research team. Participants in the control arm will be offered a FibroScan after completion of outcomes. The six-month follow up is specifically to cover those who were lost to follow up at NRN from the treatment programme.

A detailed schedule of the visits is given in Table 2.

For peer review only

For peer review only - http://bmjopen.bmj.com/site/about/guidelines.xhtml

Trial Activity	Baseline visit	3[a] Months	6[b] months
**Control group**			
Date & Time	Yes	Yes	Yes
Baseline consent	Yes	-	-
FibroScan + Feedback	-	-	Yes
Watching video stories	-	-	Yes
Qualitative interview	-	-	Yes
Demographics	Yes	-	-
AUDIT score	Yes	Yes	Yes
SADQ score	Yes	Yes	Yes
Self-reported alcohol intake[c]	Yes	Yes	Yes
Breath alcohol test	Yes	Yes	Yes
Substance misuse other than alcohol	Yes	Yes	Yes
Data on feasibility outcomes	Yes	Yes	Yes
**Intervention group**			
Date & Time	Yes	Yes	Yes
Baseline consent	Yes	-	-
FibroScan + Feedback	Yes	-	-
Watching video stories	Yes	-	-
Qualitative interview	-	-	Yes
Demographics	Yes	-	-
AUDIT score	Yes	Yes	Yes
SADQ score	Yes	Yes	Yes
Self-reported alcohol intake	Yes	Yes	Yes
Breath alcohol test	Yes	Yes	Yes
Substance misuse other than alcohol	Yes	Yes	Yes
Data on feasibility outcomes	Yes	Yes	Yes

Table 2: Work-package-three (feasibility RCT) schedule of visits and variables for data
(Alcohol Use Disorder Identification Test- AUDIT, Severity of alcohol dependence questionnaire-SADQ)

[a]3-months visit: this will be routine visit no trial-specific procedure will be carried out

[b]6 -months visit: will be a telephone consultation and/or if possible/required in person

The participant in the control group will be offered a FibroScan at 6 months if they attend it will be in-person appointment

[c]Self-reported alcohol intake in gram and unites per week

For peer review only - http://bmjopen.bmj.com/site/about/guidelines.xhtml

Data collection

At Baseline, three and six months, the following data will be collected (Table 2)

- Demographics (including address, email address and contact number). This will be archived and kept separate from the main database.
- Alcohol Use Disorder Identification Test (AUDIT) scores.
- Severity of Alcohol Dependence Questionnaire (SADQ) scores.
- Self-reported alcohol intake (gram and unit per week).
- Substance misuse other than alcohol.
- Breath alcohol testing where participants are still attending.
  Breath alcohol testing is a strength of this trial; most studies have relied on self-reporting of alcohol intake. This means we can correlate breath alcohol readings with self-reported alcohol consumption, providing substantial additional information.
- Data on feasibility outcomes (e.g., screening rate, recruitment rate, retention rate).

All the above measurements are part of routine outcome data collected by all three recruitment settings, apart from the six-month data collected for those who are no longer in a treatment programme at six months. All three services included in this trial record all of the above outcomes as part of the 12-week programme standard data set and report these to commissioners. Follow-up data is obtained at every attendance and includes the above dataset and breath alcohol testing.

Qualitative data

We will conduct one-to-one semi-structured interviews to evaluate participant's experiences of being part of the trial (e.g., "Overall, how do you feel about taking part in the KLIFAD trial?") and any changes they may have made to their lives (e.g., "Do you think the KLIFAD trial changed your use of alcohol in any way?"). The preliminary qualitative interview schedule topic guide is provided in supplementary material (SP- Qualitative interview guide). It will be piloted before use by the PPI group to check structure and wording of questions. A readiness to change model used in WP1 will also be piloted. Focus groups and interviews will be audio-recorded and transcribed by an independent transcriber approved by the sponsor, to enable thematic analysis.

Health economics

Routine NHS data collected for the standard care 12-week treatment programmes will be used together with resources utilisation derived from the NHS digital linked data to derive healthcare costs and the potential benefits of the intervention.

Outcomes

The outcomes are designed to assess the feasibility and acceptability of the KLIFAD intervention and research processes to help inform a future large-scale RCT. The following outcomes will be reported:

- Recruitment rate.
- Retention rate.
- Consent rate.
- Acceptability of the intervention (FibroScan and ARVS).
- The willingness of participants to be randomised to trial arms.
- Acceptability of the intervention to patients.

For peer review only - http://bmjopen.bmj.com/site/about/guidelines.xhtml

- Participant adherence.
- Feasibility of outcome measures.

These feasibility outcomes will enable the trial team to:

- Determine the best primary endpoint for the future definitive trial.
- Provide sample size estimates for the future definitive trial.
- Record ARVS which will contribute to the video library used in a later large-scale RCT.

Statistical and data analysis plan

The analyses of the quantitative data will be in line with Consolidated Standards of Reporting Trials (CONSORT) guidelines for pilot and feasibility trials [34]. Sekhon et al's (2017) framework for acceptability testing will be used[35]. The primary descriptive analyses will be on an intention-to-treat basis (that is, participants are analysed in the group to which they were originally allocated). Data will be summarised using frequency (%), mean (SD) or median (IQR) depending on the distribution of the data. Summary measures will be presented along with their 95% confidence intervals whenever appropriate. Results of the data analysis will be presented using appropriate tables and graphs.

The trial is not powered to investigate statistical significance between the two arms. As this is a feasibility trial, no subgroup analysis is planned. However, the results of the feasibility variables will be presented by categories of different variables (age, gender, ethnicity, severity of alcohol misuse).

Different techniques will be followed to maximise the completeness of data collection (for example via staff training). The level of missing data will be assessed. This is especially useful for the proposed primary outcome variables. An interim analysis is not planned for this trial, but the progress of the trial will be reported to the oversight committee who can assess any concerns.

Thematic analysis of qualitative data will be conducted following Braun and Clarke's standard methods[36]. Care will be taken to integrate updated guidelines about thematic analysis including a transparent appreciation of researcher reflexivity[36]. If the trial management group feel the analysis requires external validity, a sample of transcripts identified by a random number generator with the codebook will be given to a researcher independent of the trial. This will allow us to calculate the % agreement and Cohen's Kappa value (using criteria by Cohen, 1960)[37]. The Consolidated Criteria for Reporting Qualitative Studies (COREQ) will be used to ensure thorough and explicit reporting of qualitative data in reports and manuscripts for publication [38].

Ethics and dissemination

Ethical approval

The trial received favourable ethical approval from the West of Scotland Research Ethics Service (WoSRES) on 20th January 2021, REC reference: 20/WS/0179.

Informed consent

All participants will provide a written or online (e-consent) informed consent before any research activities are initiated. A PIS written in plain language will be provided and it will be

For peer review only - http://bmjopen.bmj.com/site/about/guidelines.xhtml

ensured the participant has understood the trial information and had enough time to make an informed decision. The Site Investigator will be available to answer any questions about trial participation.

Data handling and record-keeping

In compliance with the ICH/Good Clinical Practice guidelines, regulations and following the Nottinghamshire Healthcare NHS Foundation Trust SoPS, the Chief or local Principal Investigator will maintain all records and documents regarding the conduct of the trial. These will be retained for at least 24 months or for longer if required. If the responsible investigator is no longer able to maintain the trial records, a second person will be nominated to take over this responsibility. The routinely collected clinical data will be treated in the same way as other clinical case records are treated in the NHS following relevant policies developed by Nottinghamshire Healthcare NHS Foundation Trust, the UK Government, and the National Institute for Health Research (NIHR).

The Trial Master File and trial documents held by the Chief Investigator on behalf of the Sponsor shall be finally archived at secure archive facilities at the Nottingham Digestive Diseases Biomedical Research Centre (NDDC) at Nottingham University Hospital NHS Trust (NUHT). This archive shall include all trial databases and associated meta-data encryption codes.

An index will be created for Case Report Forms (CRFs) and paper trial data before storage. All online and IT-based data will be password protected and access will only be granted to people directly involved in trial and data analysis. All patient identifiable data will be pseudonymised with a trial-specific participant number.

The information will be copied to the research database (REDCAP cloud) run by the NUHT. We will delete any information that identifies participants by the end of the KLIFAD trial (currently expected October 2022). All relevant UK data protection laws will be followed, including the 2018 Data Protection Act.

Participant safety

There is a risk that being given a normal FibroScan result may provide false reassurance and encourage participants to maintain their current level of harmful drinking or encourage them to drink more. It is also possible that a high reading will generate anxiety. The trial is designed to minimise these risks by providing scripted feedback (WP1) and watching ARVS (WP2).

Cirrhosis diagnosis and FibroScan: It is anticipated that a small number of people will be identified who have previously unknown cirrhosis and so would be at risk of complications of liver disease. This will be mitigated by offering onward referral to out-patient Hepatology for all participants with a FibroScan reading >15 Kilopascal(kPa). This will be via contact with the participant's GP and would follow the current NUHT Nottinghamshire adult liver disease stratification pathway for referral[39]. Some risk mitigations will be through the feedback included in this trial which covers cirrhosis.

For WP1 and WP2, we cannot foresee any potential risks except possible emotional distress during participation in a focus group or semi-structured interview. Participants can choose to skip any question that they prefer not to answer. If distress occurs during the WP3 trial visit,

For peer review only - http://bmjopen.bmj.com/site/about/guidelines.xhtml

we will ask the participant to take a break to recover, or they can choose to terminate the process. We do not expect that the trial will cause any discomfort or pose any disadvantages, however, contact details for the trial team are provided should the participant have any questions before, during, or after taking part. We have also provided a list of locally relevant support services at the end of each patient information sheet, which participants can consult.

Patient and Public Involvement (PPI)

The trial has a dedicated PPI group and has considerable regular input from PPI group at every stage of the study.

Dissemination

The results of the feasibility trial will be submitted for publication to a peer-reviewed journal and presented at relevant conferences. A separate manuscript on the qualitative aspect of the trial will be written as well. This work is part of a PhD for the lead author (MS) who will present and submit data as a PhD thesis to the University of Nottingham. The work will also be made available to trial participants via the NDDC Biomedical Research Unit website.

For peer review only

For peer review only - http://bmjopen.bmj.com/site/about/guidelines.xhtml

References

1. Williams R, Aithal G, Alexander GJ, et al. Unacceptable failures: the final report of the Lancet Commission into liver disease in the UK. *Lancet (London, England).* 2020;395(10219):226-239.
2. World Health Organisation (WHO) 2018 global stats report on alcohol and health.pdf. 2018. URL: https://www.who.int/publications/i/item/9789241565639. Accessed 01/07/2021
3. Williams R, Aspinall R, Bellis M, et al. Addressing liver disease in the UK: a blueprint for attaining excellence in health care and reducing premature mortality from lifestyle issues of excess consumption of alcohol, obesity, and viral hepatitis. *Lancet.* 2014;384(9958):1953-1997.
4. Osna NA, Donohue TM, Jr., Kharbanda KK. Alcoholic Liver Disease: Pathogenesis and Current Management. *Alcohol research : current reviews.* 2017;38(2):147-161.
5. BritishLiverTrust. <2019 British liver trust The-alarming-impact-of-liver-disease. URL: https://www.britishlivertrust.org.uk/wp-content/uploads/The-alarming-impact-of-liver-disease-FINAL-June-2019.pdf. Accessed 01/07/2021
6. Parker R. The natural history of alcohol-related liver disease. *Current Opinion in Gastroenterology.* 2020;36(3).
7. Public Health England (PHE), Statistics on Alcohol, England 2019 [PAS] - NHS Digital.pdf>. 2019. URL: https://digital.nhs.uk/data-and information/publications/statistical/statistics-on-alcohol/2019. Accessed 01/07/2021
8. House of Commons Health Committee. Government's Alcohol Strategy. Third Report of Session.pdf>. 2012. URL: https://publications.parliament.uk/pa/cm201213/cmselect/cmhealth/132/132.pdf. Accessed 01/07/2021
9. Public Health England (PHE). Statistics on Alcohol, England 2020. 2020. URL; https://www.gov.uk/government/statistics/statistics-on-alcohol-england-2020. Accessed 01/07/2021
10. Julien J, Ayer T, Bethea ED, Tapper EB, Chhatwal J. Projected prevalence and mortality associated with alcohol-related liver disease in the USA, 2019–40: a modelling study. *The Lancet Public Health.* 2020;5(6):e316-e323.
11. O'Donnell A, Anderson P, Newbury-Birch D, et al. The impact of brief alcohol interventions in primary healthcare: a systematic review of reviews. *Alcohol and alcoholism (Oxford, Oxfordshire).* 2014;49(1):66-78.
12. Kaner EF, Beyer F, Dickinson HO, et al. Effectiveness of brief alcohol interventions in primary care populations. *The Cochrane database of systematic reviews.* 2007(2):Cd004148.
13. The National Institue fro Health and Care Excellence (NICE). Alcohol-use disorders: prevention. 2010. URL: https://www.nice.org.uk/guidance/ph24. Accessed 15/07/2021
14. Roberts SE, John A, Brown J, Napier DJ, Lyons RA, Williams JG. Early and late mortality following unscheduled admissions for severe liver disease across England and Wales. *Aliment Pharmacol Ther.* 2019;49(10):1334-1345.
15. Harman DJ, Ryder SD, James MW, et al. Direct targeting of risk factors significantly increases the detection of liver cirrhosis in primary care: a cross-sectional diagnostic study utilising transient elastography. *BMJ Open.* 2015;5(4):e007516.
16. Harman DJ, Ryder SD, James MW, et al. Obesity and type 2 diabetes are important risk factors underlying previously undiagnosed cirrhosis in general practice: a cross-sectional study using transient elastography. *Aliment Pharmacol Ther.* 2018;47(4):504-515.
17. Subhani M, Knight H, Ryder S, Morling JR. Does Advice Based on Biomarkers of Liver Injury or Non-Invasive Tests of Liver Fibrosis Impact High-Risk Drinking Behaviour: A Systematic Review With Meta-analysis. *Alcohol and Alcoholism.* 2021.
18. Rennick-Egglestone S, Ramsay A, McGranahan R, et al. The impact of mental health recovery narratives on recipients experiencing mental health problems: Qualitative analysis and change model. *PloS one.* 2019;14(12):e0226201-e0226201.

For peer review only - http://bmjopen.bmj.com/site/about/guidelines.xhtml

19. White WL. Addiction recovery: its definition and conceptual boundaries. *Journal of substance abuse treatment.* 2007;33(3):229-241.
20. Tracy K, Wallace SP. Benefits of peer support groups in the treatment of addiction. *Subst Abuse Rehabil.* 2016;7:143-154.
21. Ashford RD, Brown AM, Curtis B. Substance use, recovery, and linguistics: The impact of word choice on explicit and implicit bias. *Drug Alcohol Depend.* 2018;189:131-138.
22. Zimmerman GL, Olsen CG, Bosworth MF. A 'stages of change' approach to helping patients change behavior. *Am Fam Physician.* 2000;61(5):1409-1416.
23. Krueger RA. *Focus groups: A practical guide for applied research.* Thousand Oaks, CA, US: Sage Publications, Inc; 1988.
24. Rennick-Egglestone S, Morgan K, Llewellyn-Beardsley J, et al. Mental Health Recovery Narratives and Their Impact on Recipients: Systematic Review and Narrative Synthesis. *Canadian journal of psychiatry Revue canadienne de psychiatrie.* 2019;64(10):669-679.
25. Ng F, Charles A, Pollock K, et al. The mechanisms and processes of connection: developing a causal chain model capturing impacts of receiving recorded mental health recovery narratives. *BMC psychiatry.* 2019;19(1):413-413.
26. Rennick-Egglestone S, Elliott R, Smuk M, et al. Impact of receiving recorded mental health recovery narratives on quality of life in people experiencing psychosis, people experiencing other mental health problems and for informal carers: Narrative Experiences Online (NEON) study protocol for three randomised controlled trials. *Trials.* 2020;21(1):661.
27. Charles Teddlie FY. Mixed Methods Sampling A Typology With Examples. *Journal of Mixed Methods Research.* 2007;1(1).
28. STOTLAND NL, MATTSON MG, BERGESON S. The Recovery Concept: Clinician and Consumer Perspectives. *Journal of Psychiatric Practice®.* 2008;14:45-54.
29. Bowen DJ, Kreuter M, Spring B, et al. How we design feasibility studies. *Am J Prev Med.* 2009;36(5):452-457.
30. Public helath England (PHE). The international evidence on the prevention of drug and alcohol use Summary and examples of implementation in England. https://assets.publishing.service.gov.uk/government/uploads/system/uploads/attachment_data/file/774743/Preventing_drug_and_alcohol_misuse__international_evidence_and_implementation_examples.pdf. Published 2015. Accessed 15/07/2021
31. Sim J, Lewis M. The size of a pilot study for a clinical trial should be calculated in relation to considerations of precision and efficiency. *Journal of clinical epidemiology.* 2012;65(3):301-308.
32. Lancaster GA, Dodd S, Williamson PR. Design and analysis of pilot studies: recommendations for good practice. *Journal of evaluation in clinical practice.* 2004;10(2):307-312.
33. Browne RH. On the use of a pilot sample for sample size determination. *Statistics in medicine.* 1995;14(17):1933-1940.
34. Consolidated Standards of Reporting Trials (CONSORT). Consort Statement 2010. URL: http://www.consort-statement.org/. Accessed 01/07/2021.
35. Sekhon M, Cartwright M, Francis JJ. Acceptability of healthcare interventions: an overview of reviews and development of a theoretical framework. *BMC Health Services Research.* 2017;17(1):88.
36. Braun V, Clarke V. Reflecting on reflexive thematic analysis. *Qualitative Research in Sport, Exercise and Health.* 2019;11(4):589-597.
37. Cohen J. A Coefficient of Agreement for Nominal Scales. *Educational and Psychological Measurement.* 1960;20(1):37-46.
38. Tong A, Sainsbury P, Craig J. Consolidated criteria for reporting qualitative research (COREQ): a 32-item checklist for interviews and focus groups. *International Journal for Quality in Health Care.* 2007;19(6):349-357.
39. Chalmers J, Wilkes E, Harris R, et al. The Development and Implementation of a Commissioned Pathway for the Identification and Stratification of Liver Disease in the Community. *Frontline Gastroenterol.* 2020;11(2):86-92.

For peer review only - http://bmjopen.bmj.com/site/about/guidelines.xhtml

Authors' contributions

Mohsan Subhani:

KLIFAD trial: The project is part of PhD thesis. Trial coordinator and member of trial management group. He has and will contribute to following; research idea, funding application, PPI meetings, trial protocol, IRAS application and ethical approval, FibroScan training, site initiation, work package 1 focus group, work package 2 alcohol recovery story recording, monthly trial management group meeting, monitoring ongoing progress of work package 3, qualitative interview, data synthesis and analysis, report writing, dissemination.

Manuscript: Written initial draft of the protocol, implemented changes, and drafted final version of protocol and manuscript.

Katy Jones:

KLIFAD trial: Member of trial management group. She is supervising the qualitative component of the trial including conducting and analysing semi structured interviews.

Manuscript: Reviewed protocol and manuscript, provided specialist input for qualitative aspects of the protocol and contributed to the final manuscript.

Kirsty Sprange:

KLIFAD trial: Member of trial management group. She contributed to following; research idea, funding application, trial protocol, IRAS application, work package 3 initiation, trial management and progress.

Manuscript: Reviewed protocol and manuscript and contributed to the final manuscript.

Stefan Rennick-Egglestone:

KLIFAD trial: Member of trial management group. He is supervising work package 2 including proposal for alcohol recovery stories recording, editing, and finalising.

Manuscript: Reviewed protocol and manuscript, provided specialist input for work-package-2 of protocol and contributed to final manuscript

Holly Knight:

KLIFAD trial: Member of trial management group. She is contributing to work package 1 including developing FibroScan results feedback scripts and organising focus groups.

Manuscript: Reviewed protocol and manuscript, provided specialist input for work-package-1 of protocol and contributed to final manuscript

Joanne R Morling:

KLIFAD trial: Member of trial management group. She PhD supervisor for Dr Subhani, supervising trial overall and specifically helping with health economics part of trial.

Manuscript: Reviewed protocol and manuscript, provided specialist input for health economics section of protocol and contributed to final manuscript

Doyo G Enki:

KLIFAD trial: Member of trial management group. He is Statistical support for the trial.

Manuscript: reviewed final manuscript

Andrew Wragg:

KLIFAD trial: Patient and public involvement coordinator.

Manuscript: reviewed final manuscript

Stephen D Ryder:

KLIFAD trial: Chief investigator, PhD supervisor for Dr Subhani and member of trial management group. He has contributed to following; research idea, funding application, PPI meetings, trial protocol, IRAS application and ethical approval, overall supervision of all three work packages, data synthesis and analysis, report writing, dissemination.

For peer review only - http://bmjopen.bmj.com/site/about/guidelines.xhtml

Funding statement

This work was supported by “National Institute for Health Research (NIHR)” grant number [RfPB NIHR201146]. JRM receives salary support from a Medical Research Council Clinician Scientist award (grant number MR/P008348/1).

Data sharing statement

The anonymised data that will support the findings of this trial will be available from the corresponding author, [MS], upon reasonable request.

Competing interests’ statement

No competing interests from any author

Figure Legends

Figure 1: The KLIFAD Trial timeline and flow chart (RCT-randomised control trial, GCP-good clinical practice, AUDIT-alcohol use disorder identification test, SADQ-severity of alcohol dependence questionnaire). The trial has three work packages (WP), WP3 is in randomised feasibility trial.

Figure 2: Flow diagram for; Work package one to create FibroScan scripted feedback and Work package two to create alcohol recovery videos stories

Figure 3: Flow diagram for work-package-three the randomised control trial flow chart (RCT-randomised control trial, GCP-good clinical practice, AUDIT-alcohol use disorder identification test, SADQ-severity of alcohol dependence questionnaire). Work package three is feasibility randomised control trial.

For peer review only

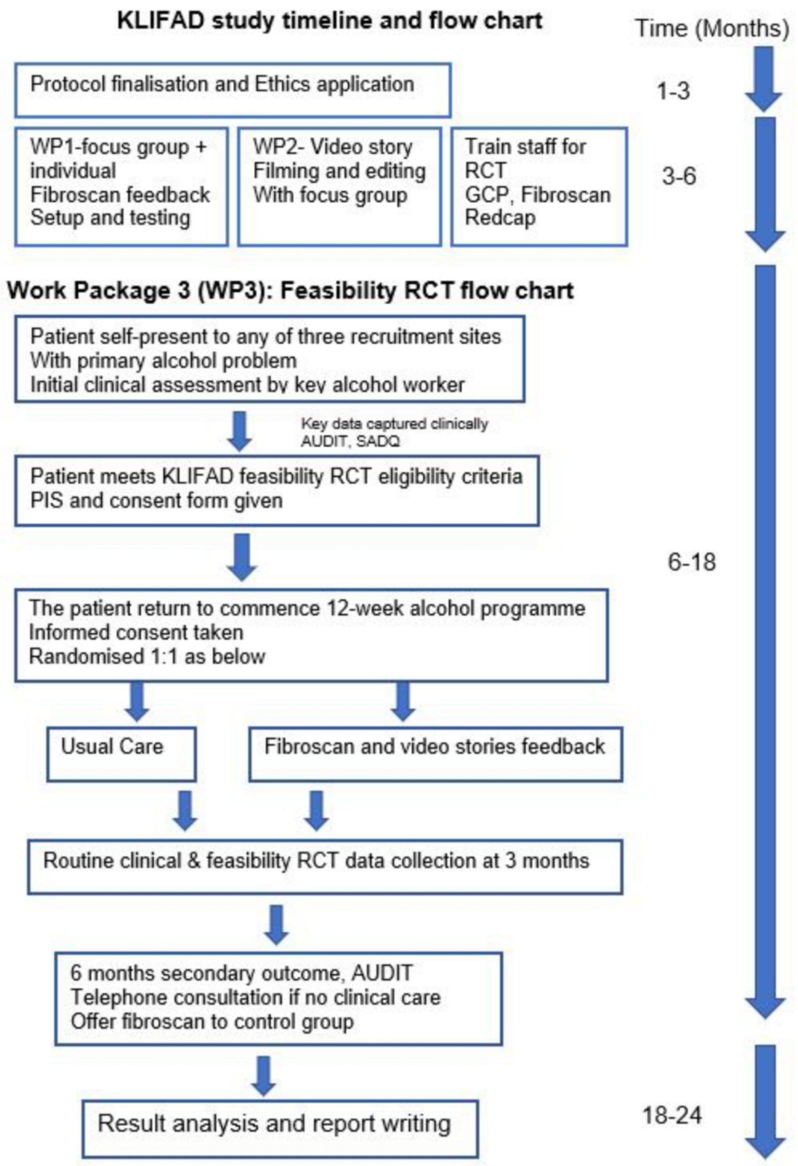

Figure 1: The KLIFAD trial timeline and flow chart (RCT-randomised control trial, GCP-good clinical practice, AUDIT-alcohol use disorder identification test, SADQ-severity of alcohol dependence questionnaire). The trial has three work packages (WP), WP3 is in randomised feasibility trial.

149x197mm (300 x 300 DPI)

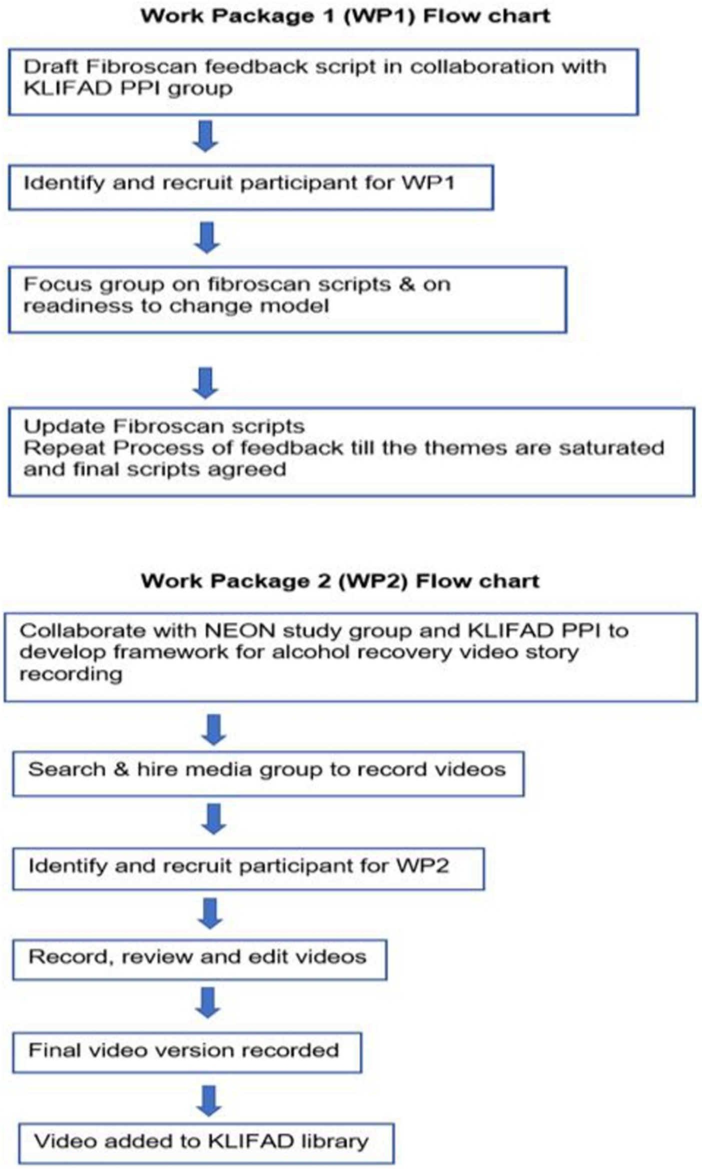

Figure 2: Flow diagram for; Work-package 1 to create fibroscan scripted feedback and Work-package 2 to create alcohol recovery videos stories

147x216mm (300 x 300 DPI)

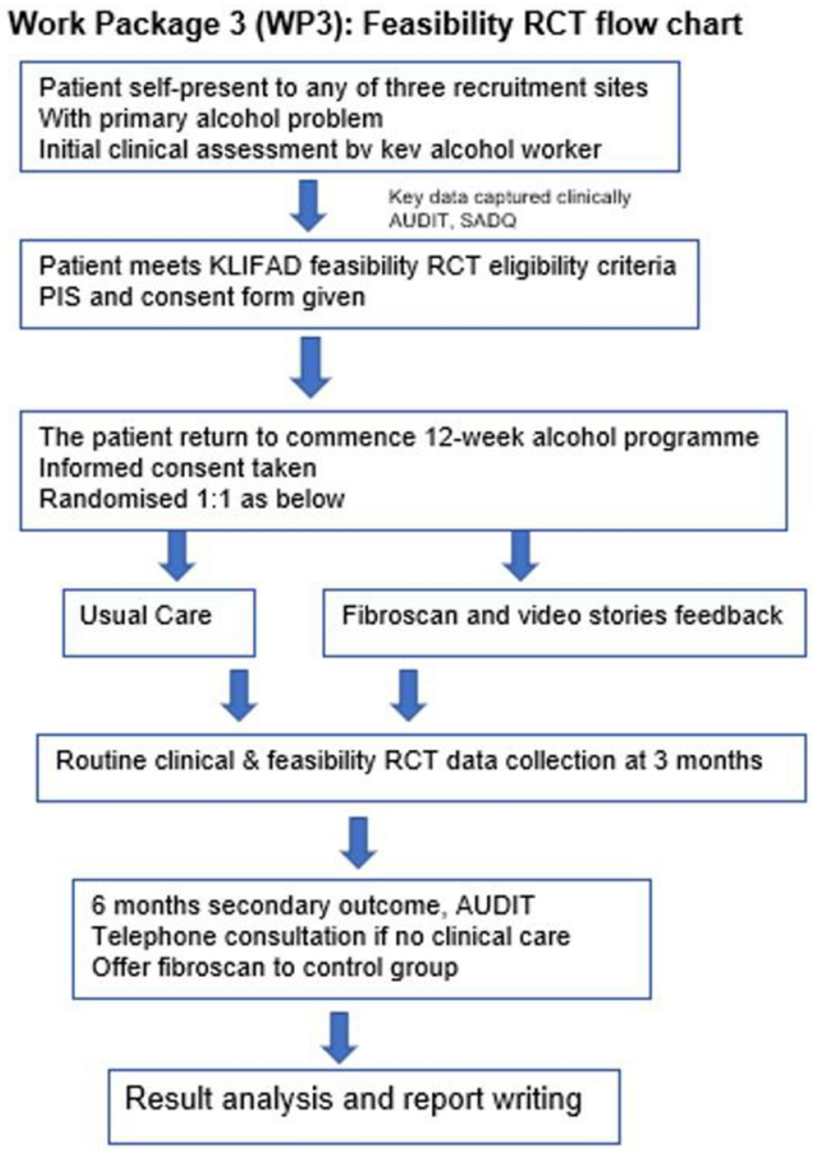

Figure 3: Flow diagram for work-package-3 the randomised control trial flow chart (RCT-randomised control trial, GCP-good clinical practice, AUDIT-alcohol use disorder identification test, SADQ-severity of alcohol dependence questionnaire). Work-package 3 is feasibility randomised control trial.

149x202mm (300 x 300 DPI)

Nottingham Recovery Network

Assessment Form

Clean Slate
Criminal Justice Substance Misuse Service

OFFICE USE ONLY

Date received:<br>Referred by:<br>If SELF, how did they hear about the service:		Client Id:<br>Assessed by:<br>Assessment location:<br>Date:
Specific risk / need identified:	Probation<br>Y / N	

CLIENT DETAILS

GDPR: There is a privacy notice in each room - please read it

DRUG ☐
DRUG & ALCOHOL ☐
ALCOHOL ☐

If drug(s) please state type:

Title: Mr / Mrs / Ms / Miss / other (please state)

First name: **Surname:**

Prefers to be known as:

Gender: Male ☐ Female ☐ Other ☐ Not specified ☐

Date of Birth: **Age:**

Address:

Postcode:

Home Tel:

Mobile Tel:

Email address:

G.P Name:

Address:

Tel:

Currently receiving treatment? Y / N
Currently on prescribed medication? Y / N
Seen by GP in last month? Y / N
GP aware of substance misuse? Y / N

Pharmacy current/preferred:

Permission to be contacted: Home Visit ☐ Letter ☐ Telephone ☐ Text ☐ Email ☐

Emergency Contact: **Telephone:**

Medication: Current ☐ Recent Past ☐ Past ☐ None ☐

Type & Dosage – List

....

....

Prescribed by:

Known Allergies: Y / N

List:

....

....

....

Barriers to Accessing Treatment? (include any disabilities)

Preferred Language:

Is an interpreter required Y / N

For peer review only

For peer review only - http://bmjopen.bmj.com/site/about/guidelines.xhtml

Client initials: DoB:

DEMOGRAPHICS

Sexuality: ☐ Heterosexual ☐ Gay/Lesbian ☐ Bi-sexual ☐ Other ☐ Not stated

Marital Status: ☐ Single ☐ Cohabiting ☐ Married ☐ Separated ☐ Divorced ☐ Widowed

Religion: ☐ None ☐ Christian ☐ Buddhist ☐ Hindu ☐ Jewish ☐ Muslim ☐ Sikh ☐ Other ☐ Not stated

Nationality: ☐ UK ☐ Other (please state) ....

Ethnic category

White	Mixed	Asian/Asian British	Black/Black British	Other Ethnic
British ☐	White & Black Caribbean ☐	Indian ☐	Caribbean ☐	Chinese ☐
Irish ☐	White & Black African ☐	Pakistani ☐	African ☐	Any Other ☐
Other White ☐	White & Asian ☐	Bangladeshi ☐	Black British ☐	Not Known ☐
	Other Mixed ☐	Other Asian ☐	Other Black ☐	Not Stated ☐

Accommodation:
☐ Homeless – urgent housing problems
☐ Housing problems –i.e. staying with friends
☐ Owner occupier
☐ Tenant - Landlord (please specify) ....

Employment Status:
☐ Employed (full/part time)
☐ Long term sick or disabled
☐ Retired from paid work
☐ Unemployed and seeking work
☐ Other (please specify)..
☐ Homemaker
☐ Not receiving benefits
☐ Student
☐ Unpaid voluntary work

Time since last employed: ☐ Never employed ☐ Currently employed ☐ Less than 1 year ☐ 1 – 2 years ☐ 2 – 3 years ☐ >3 years (please state) ....

Sex Worker: Yes ☐ No ☐ **Current / Previous**
If **YES** working from premises or street ....

Ex Service Personnel: Yes ☐ No ☐ **Referral to Royal British Legion wanted?** Yes ☐ No ☐

Disability: Yes ☐ No ☐ Type ....

Carer: Yes ☐ No ☐ Support needs..

Debt Issues: Yes ☐ No ☐ Support wanted ....

Support services already engaged with:

Support wanted:

Treatment Goal:

For peer review only - http://bmjopen.bmj.com/site/about/guidelines.xhtml

Client initials: DoB:

PRESENTATION ON ASSESSMENT

(Intoxicated / withdrawing / appearance etc.)

SUBSTANCE MISUSE PAST 7 DAYS / 4 WEEKS

For peer review only

For peer review only - http://bmjopen.bmj.com/site/about/guidelines.xhtml

Client initials: DoB:

SUBSTANCE MISUSE HISTORY

(Why, when, who with, route, frequency, finances, motivations, withdrawal symptoms, harm minimisation)

For peer review only

Client initials: DoB:

TREATMENT HISTORY

Currently in drug or alcohol treatment Yes ☐ No ☐

Name of all agencies/services currently in contact with client and keyworkers' names (where applicable):

Previously sought help with substance use Yes ☐ No ☐

Previously received structured drug or alcohol treatment Yes ☐ No ☐

Date of first treatment episode:

Further details

PROBLEMATIC SUBSTANCE USE

Substance		Frequency (in last 28 days)	Amount/Units /Cost	Route	Age of 1st use
**Primary**					
**2nd**					
**3rd**					

Drug Screening Questionnaire (DAST)

Score ....

INJECTING STATUS

Currently injecting ☐ Previously ☐ Never injected ☐ Declined to answer ☐

ALCOHOL

Drinking Days .... /28 Units/day .... Units/week..

AUDIT .... SADQ....................................... Breath Alcohol: ....Mg/l BrAC

BBV

HIV Status: Negative ☐ Positive ☐ Not Known ☐ **Latest Test Date** ....

Hep C – Intervention Status

Offered and accepted ☐ **Hep C Tested** Yes ☐ No ☐ **Latest Test Date** ....

Offered and refused ☐

Not appropriate to offer ☐ **Hep C Positive** Yes ☐ No ☐ Not Known ☐

Hep B - Intervention Status

Offered and accepted ☐ **Vaccination Count:** 1 Vaccination ☐

Offered and refused ☐ 2 Vaccinations ☐

Immunised already ☐ 3 Vaccinations ☐

Not appropriate to offer ☐ Course Complete ☐

Referred For Hepatology Yes ☐ No ☐

For peer review only - http://bmjopen.bmj.com/site/about/guidelines.xhtml

For peer review only

Client initials: DoB:

PHYSICAL HEALTH

Does the client consider themselves to have a disability? Yes ☐ No ☐

Nature of disability

Does the client have any health problems? Yes ☐ No ☐

(allergies, asthma, epilepsy, diabetes, dental, women specific, sexual health, cardiac, respiratory, DVT, sleep, diet)

Smoker: No ☐ Yes ☐ Qty .... p/day

Does client have smoke alarm: Yes ☐ No ☐ **Referral made to Framework service**: Yes / declined

IMMEDIATE RISK IDENTIFIED: Physical Health & Overdose

Prompts: Regular injector / Injecting in high-risk areas / Poly substance use / Poly substance use – opiates and alcohol / Substance use related seizures or DTs? / Injects alone / Witnessed overdose by others / History of past overdose

Level of risk: No risk ☐ Low ☐ Medium ☐ High ☐

Likelihood of occurrence No risk ☐ Low ☐ Medium ☐ High ☐

ACTION(S):

For peer review only

Client initials: DoB:

PSYCHOLOGICAL HEALTH

Any current/historical contact with mental health services: Yes ☐ No ☐
(mental health diagnosis or symptoms, negative thoughts, self esteem, current mood, history of suicidal thoughts/acts, self-harm)

Mental Health treatment need Yes ☐ No ☐

Current services involved ....

Ever experienced overdose? Yes ☐ No ☐ Accidental ☐ Deliberate ☐

Date & Drugs involved

Treatment received

Has Naloxone been offered (please circle) **Accepted / Refused**

IMMEDIATE RISK IDENTIFIED: Individual

Prompts: Suicidal / Self-harm / Mental health / Domestic violence
Trigger: Where DV is identified complete Multi Agency Domestic Violence Risk Identification Form when appropriate

Level of risk: No risk ☐ Low ☐ Medium ☐ High ☐

Likelihood of occurrence No risk ☐ Low ☐ Medium ☐ High ☐

ACTION(S):

IMMEDIATE RISK IDENTIFIED: Personal Safety / Self Neglect

Prompts: Reliant on others / Difficulty in coping with everyday domestic tasks / Homeless/vulnerably housed / Recent threat (s) from others /Financial Vulnerability

Level of risk: No risk ☐ Low ☐ Medium ☐ High ☐

Likelihood of occurrence No risk ☐ Low ☐ Medium ☐ High ☐

DETAILS:

ACTION(S):

For peer review only - http://bmjopen.bmj.com/site/about/guidelines.xhtml

Client initials: DoB:

PARENTAL STATUS

Do you have any contact with children under 18yrs?: Yes ☐ No ☐

Children/Partner's Children: Yes ☐ No ☐ **Sole Carer:** Yes ☐ No ☐ **Other** ....

Do all/some of the children live with you?: All of the time ☐ Some of the time ☐ No ☐

No. of children: **Ages:**

Are any of the client's children (biological, step, foster, adoptive, guardian) or any of the children receiving early help or are they in contact with Children's Social Care?:

Child in need ☐ Early help ☐ Has a child protection plan ☐ Looked after child ☐ None ☐

Social Care Services Involved: Current ☐ Recent past ☐ Past ☐ None ☐

Further details

Is client or partner pregnant: Yes ☐ No ☐ **Due Date:**

Referred to Specialist Midwife Yes ☐ No ☐ Previous ☐

IMMEDIATE RISK IDENTIFIED: Child Care

Prompts: Currently pregnant / Responsible for any child(ren) / Intoxicated while solely responsible for child(ren)
Trigger: Also complete "Childcare & Family support Form" in line with guidance notes if required

Level of risk: No risk ☐ Low ☐ Medium ☐ High ☐

Likelihood of occurrence No risk ☐ Low ☐ Medium ☐ High ☐

ACTION(S):

Safe storage box issued Yes ☐ No ☐ Refused ☐

Childcare & Family Support Form completed Yes ☐ No ☐

Family/Carer Support Offered: Yes ☐ No ☐ Type..

For peer review only

FAMILY & RELATIONSHIPS

(family health and significant relationships, child protection, care issues, vulnerable adults, support networks)

DOMESTIC VIOLENCE

Have you ever been a victim or perpetrator of domestic violence?

Survivor ☐
Perpetrator ☐
Declined to answer ☐

Current ☐
Recent past ☐
Past ☐
None ☐

IMMEDIATE RISK IDENTIFIED: Domestic Violence

Where DV is identified complete Multi Agency Domestic Violence Risk Identification Form when appropriate

Level of risk:	No risk ☐	Low ☐	Medium ☐	High ☐
Likelihood of occurrence	No risk ☐	Low ☐	Medium ☐	High ☐

ACTION(S):

Black DV card issued ☐ **DASH form completed** ☐ **MARAC referral made** ☐

For peer review only - http://bmjopen.bmj.com/site/about/guidelines.xhtml

CRIMINAL JUSTICE/OFFENDING HISTORY

Current Criminal Justice Status (tick all that apply)

Community Order	☐	DRR	☐	RAR Days	☐
Licence	☐	ATR	☐	Suspended Sentence	☐
Sex Offender Registration	☐	Prolific Offender	☐	ROB	☐
MAPPA	☐	Paying Fines	☐	Schedule 1 Offender	☐

Further details of current criminal justice status:
(length of orders, name of workers involved)

Details of past criminal justice / offending history
(offences committed, length of sentences, targets of violence)

Any record of violent offences?
Further details:

IMMEDIATE RISK IDENTIFIED: Harm to Others

Prompts: Violence to others / Aggression to others / ever used a weapon in assault? / Ever made any preparations to commit harm to others? / Criminal record / Violence/ Domestic Violence/ Aggression towards professionals

Trigger: Where DV is identified complete Multi Agency Domestic Violence Risk Identification Form when appropriate

Level of risk:	No risk ☐	Low ☐	Medium ☐	High ☐
Likelihood of occurrence	No risk ☐	Low ☐	Medium ☐	High ☐

ACTION(S):

For peer review only - http://bmjopen.bmj.com/site/about/guidelines.xhtml

EXISTING RECOVERY CAPITAL

(Including information around housing, financial, education, employment, cultural issues, offending as well as drug & alcohol use)

For peer review only

DVLA / Occupational Concerns / Other

For peer review only - http://bmjopen.bmj.com/site/about/guidelines.xhtml

PERCEPTION OF ONGOING NEEDS & ACTIONS

Signposted / referred	☐ Debt advice	☐ CGL Jigsaw (ExFam)	☐ GP
	☐ Health Shop	☐ Housing Aid	☐ Housing Crisis
	☐ Smoking cessation	☐ Street Outreach	☐ Wellness in Mind
	☐ Other (please state)		

Next appointment date and time: Location: Worker:

Keyworker signature: Date:

Print Name:

DVLA Guidelines for people receiving treatment for Alcohol and/or Drug related difficulties.

You have stated that you hold a current driving licence and continue to drive. As such you have been issued with guidance relating to Alcohol &/or Drug and driving as identified above. Please sign to acknowledge your responsibility to act according to this guidance.

Signed: Date:

Print Name:

For peer review only - http://bmjopen.bmj.com/site/about/guidelines.xhtml

For peer review only

SP-Definitions

The following definitions are relevant to the KLIFAD trial:

Recovery Definition
For the KLIFAD trial we adopted the following definition of "Recovery"

"A period of sustained abstinence from alcohol creating a deeply personal, unique process of change, a way of living a satisfying, hopeful and contributing life even with limitations caused by illness. A process involving the development of new meaning or purpose in one's life which maximises health and wellbeing and participation in the rights, roles and responsibilities of society"[1-4].

Recovery story
A story told by a person about their journey of recovery.

In KLIFAD we are using recovery stories which are primarily first-person lived experience accounts, which include elements of both adversity/struggle and of strength/success/survival related to AUD, and which refer to events or actions over a period. Some stories will include brief fragments presenting clinical perspectives on a case, provided by a clinician who worked with the narrator[5].

Story narrator
The person telling their own recovery story.

Story recipient
The person viewing, reading or listening to someone else's recovery story.

KLIFAD Library
A collection of recovery stories intended for use in the KLIFAD feasibility trial.

Alcohol misuse
The National Institute on Alcohol Abuse and Alcoholism (NIAAA) define alcohol misuse as "alcohol consumption that puts individuals at increased risk for adverse health and social consequences"[6]

Alcohol use disorders
The NIAAA define AUD as "a chronic relapsing brain disorder characterized by an impaired ability to stop or control alcohol use despite adverse social, occupational, or health consequences" [6].

For peer review only - http://bmjopen.bmj.com/site/about/guidelines.xhtml

SP-Focus Group Guide WP1 V2.0

Focus group Guide participants
Work Package 1 (WP1)
Version 2.0 Date:14/10/2020

***Study title:* Does knowledge of liver fibrosis affect high-risk drinking behaviour (KLIFAD)? A feasibility randomised controlled trial**

To begin

Welcome to the focus group session. Thanks for taking the time to join us to talk about liver disease screening.

You were invited here today because you attended a liver scan appointment and were given your level of risk for liver disease using a Fibroscan machine. We would like to understand how to provide the best experience for patients undergoing the scan. This includes how the person operating the Fibroscan machine discusses the scan itself and then delivers the results of the scan to patients. We will ask you to read through a script we have prepared to help operators talk through the scan and also a document that provides patients with their results.

Everyone's risk of liver disease may be different. Because everyone has very different life experiences, there are no wrong answers to these questions, but rather different points of view. Please feel free to share your point of view even if it differs from what others have said. Keep in mind that we're just as interested in negative comments as positive comments, and at times the negative comments are the most helpful.

Logistics

- Focus group will last about 2 hours
- Feel free to move around
- Where is the bathroom? Exit?
- Help yourself to refreshments

Ground Rules

- Hope that everyone feels comfortable enough to participate.
- Information provided in the focus group must be kept confidential
- Stay with the group and please don't have side conversations
- Turn off mobile phones if possible
- This is an opportunity to help contribute to the treatment of liver disease!

You've probably noticed the microphone. I'm tape recording the session because I don't want to miss any of your comments. People often say very helpful things in these discussions and I can't write fast enough to get them all down.

For peer review only - http://bmjopen.bmj.com/site/about/guidelines.xhtml

For peer review only

If you talk about anyone else during the focus group by name (such as a friend or member of staff) – then we will keep their name anonymous when we write up the results by providing them with a false name. Likewise (the participant) we will also keep your identity anonymous during the write-up by giving you a false name in any reports resulting from this study

Are you okay with this? Do you have any questions?

- Answer any questions they have
- If they do not want to participate, thank them for their time and escort them out of the venue. If they have participated via telephone or over video conferencing – finish the call.

Beginning the focus group

Start recording the interview on the Dictaphone.

Firstly, I want you to think back to your liver scan appointment.

1. Did you understand why you were undergoing a fibroscan and what the scan involved?
2. What was your experience of the scan? Was there anything about the way the operator conducted the scan or talked to you about the scan that you liked/disliked/found helpful?
3. After the scan, what information were you provided with? Including your results, any feedback from the scan operator, and any other information about liver disease?
   a. Was any of this difficult to understand? What information did you find most helpful?
4. Did the scan and/or scan results prompt you to make some changes to improve your liver health?
   a. If you received normal scan results, did you still want to make lifestyle changes?

Now I'd like us to spend the rest of the session today reviewing the documents in front of you. Please take some time to read through these documents and write any thoughts you have about the wording or how the information is presented on the document.

Provide participants with pens
Give participants approximately 10-15 minutes to read through script and fibroscan results

Let's review the operator script. Imagine you were receiving this information from a fibroscan operator.

1. Do you understand the information presented in the script?
   a. What did you like/dislike about the script? What information was helpful/unhelpful? Was anything unclear?
2. Was there any information you felt was missing or that you think would make a useful addition to the script?

For peer review only - http://bmjopen.bmj.com/site/about/guidelines.xhtml

a. Do you have any suggested changes or improvements to the script?

Now let's review the fibroscan result documents. There are three different results a patient can receive, depending on their liver stiffness. Imagine you were receiving this information from a fibroscan operator.

1. Do you think the results made sense for each level of liver disease stiffness?
    a. Did you understand the information? What information was helpful/unhelpful? Was anything unclear?
2. How did the documents make you feel?
    a. Did anyone have a negative reaction/positive reaction?
3. Did you like the way the results were presented (e.g. graphically, visually)?
    a. What would you change? Would you prefer the results to be presented as a value, on a scale, on a graph etc.?
4. Would you feel confident knowing what your result was and how to go about making lifestyle changes from this information?
    a. If not, why and what could we include that would help improve your confidence? Do you think the results documents would need explaining further by the operator?
5. Does anyone have additional thoughts about a specific result document (normal, likely fibrosis, likely cirrhosis)?
    a. Do you think the information reflects the level of risk and need for behaviour change?
6. Is there any other information we should include in the results document?
    a. Do you have any suggested changes or improvements to the results?

Close

Okay, that reaches the end of the questions I wanted to ask today. Is there anything else you wanted to add or talk about that we didn't talk about today?

If you're okay to end the focus group there, I'll switch the Dictaphone off, thank you!

Debriefing

- Thank you for speaking to us.
- Provide participants with a sheet which outlines the range of services etc, go through it with them. If there is any particular service/resource that they have expressed an interest in – then signpost them to it.
    - If they have participated via telephone– a state that they can be sent this via email if this wish or it can be read out to them.
- Thank them again, and ask if they are feeling okay to leave the building/ or hang up/exit the call.

For peer review only - http://bmjopen.bmj.com/site/about/guidelines.xhtml

SP-Change model questionnaire (CMQ) V1.0

Change model questionnaire

Work package 1 (WP1) V1.0 26/10/2020

Study title: Does knowledge of liver fibrosis affect high-risk drinking behaviour (KLIFAD)? A feasibility randomised controlled trial

Your doctor may have asked you to cut down how much alcohol you are drinking. Please find the statement that best describes the way you feel right now about cutting down your alcohol use to the amount the research team recommends

- ☐ I am continuing to drink at the same level and right now I am not considering reducing how much I drink
- ☐ I am continuing to drink at the same level but and right now I am considering reducing how much I drink
- ☐ I am continuing to drink at the same level but I am planning to reduce how much I drink
- ☐ Right now I have reduced how much alcohol I drink, and have maintained this for less than six months
- ☐ Right now I have reduced how much alcohol I drink, and have maintained this for more than six months

For peer review only - http://bmjopen.bmj.com/site/about/guidelines.xhtml

SP- Qualitative interview guide

Qualitative interview Guide

Work package 3 (WP 3) Feasibility RCT

Study title: **Does knowledge of liver fibrosis affect high-risk drinking behaviour (KLIFAD)? A feasibility randomised controlled trial**

To begin

- Go over the study information again with the participant:
  - Thank you for coming to/agreeing to take part in the interview today…
  - Explain what will happen:
  - 'You'll be asked brief questions about your experience of taking part in the KLIFAD study and some questions about how you felt about taking part in this study and how it might have had an impact on you".
  - There are no 'right' or 'wrong' answers – I am not here to judge you, but to listen to your experiences as everyone's experience is valuable.
  - You can tell us as little or as much information as you want to during this interview, it is kept confidential in the research team. We may use a transcription service, but they are required to sign a confidentiality agreement and identifiers are removed from the typed-up transcript.
  - You can pause or stop the interview at any time if you want a break, you feel uncomfortable or don't want to continue with the interview.
  - After the interview, I will provide you with information about services and resources – that you may find useful if you have any concerns about what you have told us.
- Are you okay with all this? Do you have any questions?
  - Answer any questions they have
  - If they do not want to participate, thank them for their time and escort them out of the venue. If they have participated via telephone or over video conferencing – finish the call.

- Note: We will ask our PPI group about whether to include clarification of specific terms at this point. For example, relapse or lapse or teetotal/sober etc to ensure we ask questions in the participant's preferred way of talking about their alcohol use.
- If you talk about anyone else during the interview by name (such as a friend or member of staff) – then we will keep their name anonymous when we write up the results by providing them with a false name. Likewise (the participant) we will also keep your identity anonymous during the write-up by giving you a false name in any reports resulting from this study
- If you are satisfied with this, please confirm that you still consent to take part.
  - They will have already consented to take part when they signed up. Check you have received this consent (if was by e-mail or post).
  - If unsatisfied and does not want to take part – thank them for their time and guide them out of the venue/end the call.

For peer review only - http://bmjopen.bmj.com/site/about/guidelines.xhtml

Beginning the interview

Start recording the interview on the Dictaphone.

Here we can ask an introductory question to establish some rapport.

Your experience of the KLIFAD study

Q. Have you ever taken part in a research study before?

Q. Can you take me through what you remember about the KLIFAD study? (If they get into specifics of the results…. We'll touch on that later, for now, I'd like you to think about your experience of the scan process as a whole, for example how you felt about the scan or the staff who scanned you.)

Q. Overall, how do you feel about taking part in the KLIFAD study?

Follow up questions: If positive feedback: What did you particularly like?

If negative feedback: What did you not like/thought could be different?

Q. In regard to the fibroscan, did you understand why you were invited to have this scan? Did the staff give you enough information about the scan? Was there anything about the whole process you liked/didn't like?

Q. Where did you watch the stories? Did you watch it with anyone else? What was your response to them?

Your feelings about getting the KLIFAD study

Q. Can you tell me what you remember about your fibroscan scan result?

Follow-up questions: Can you remember the specific value, scale, what the value meant (potential liver disease etc)? Was the result explained clearly, did you understand it? Can

For peer review only - http://bmjopen.bmj.com/site/about/guidelines.xhtml

you think of ways to improve how we give people their scan results? Is there anything else you think would be helpful to know when you receive your scan result?

Q. Do you remember how you felt when you first got your fibroscan result? Explore their thoughts and feelings here by using reflection 'So, I'm hearing that you felt confused and a bit frightened'. Also can use follow-up questions if appropriate e.g., Can you talk a bit more about why you felt scared? Can you describe your feeling of relief? Etc.

Q. What did it feel like watch stories describing other people's experiences of receiving a fibroscan? Follow up questions: Which stories can you remember accessing? Can you describe any ways in which these made an immediate impact on you? Can you describe any ways in which these have made a longer-tem impact on you? Did you learn anything from the stories?

Q. Did you discuss the KLIFAD study with anyone?

Follow up questions: What part did you talk about? (Scan/story/both?). Who did you talk to about it? How did they feel about it? If they didn't talk to anyone about it, ask why they didn't

Q. Now that a bit of time has passed, how do you feel about taking part in the KLIFAD study?

Your use of alcohol since you took part in the KLIFAD study

Q. Can you talk about your use of alcohol at a few different time points? It may be hard to remember this far back so sometimes it's helpful to look at a calendar and plot out some key dates (e.g. birthdays, trips away) that can help you remember.

1. Your use of alcohol (if any) just before you had your fibroscan result
2. Your use of alcohol (if any) on the day or days after you had your fibroscan result
3. Your use of alcohol (if any) two weeks after you had your result
4. Your use of alcohol (if any) over the last month

Q. Do you think the KLIFAD study changed your use of alcohol in any way?

If yes: explore, how, why do they think it affected it. If no: invite them to talk about that.

Explore if they sought out additional supports e.g. AA

Follow-up: Had you thought about changing before taking part in this study?

Q. If yes to changes, what were your main reasons for making these changes?

For peer review only - http://bmjopen.bmj.com/site/about/guidelines.xhtml

Q. If no, tell me more about why you didn't want to or didn't feel able to make changes at that time.

Follow-up questions: Was there anything that helped you make the changes? Was there anything that was a barrier to making changes?

Close

Okay that reaches the end of the questions I wanted to ask you. Is there anything else you wanted to add or talk about that we didn't talk about today?

If you're okay to end the interview there, I'll switch the Dictaphone off, thank you!

Debriefing

- Thank you for speaking to us.
- How are you feeling – is there anything in the interview has troubled you or upset you?
- Provide participant with sheet which outlines range of services etc, go through it with them. If there is any particular service/resource that they have expressed an interest in – then signpost them to it.
    - If they have participated via telephone– state that they can be sent this via email if this wish or it can be read out to them.
- Thank them again, and ask if they are feeling okay to leave the building/ or hang up/exit the call.

For peer review only

For peer review only - http://bmjopen.bmj.com/site/about/guidelines.xhtml

1. Anthony WA. Recovery from mental illness: The guiding vision of the mental health service system in the 1990s. *Psychosocial Rehabilitation Journal.* 1993;16(4):11-23.
2. Rennick-Egglestone S, Morgan K, Llewellyn-Beardsley J, et al. Mental Health Recovery Narratives and Their Impact on Recipients: Systematic Review and Narrative Synthesis. *Canadian journal of psychiatry Revue canadienne de psychiatrie.* 2019;64(10):669-679.
3. Witkiewitz K, Wilson AD, Pearson MR, et al. Profiles of recovery from alcohol use disorder at three years following treatment: can the definition of recovery be extended to include high functioning heavy drinkers? *Addiction (Abingdon, England).* 2019;114(1):69-80.
4. <Policy report - A vision of recovery_ UKDPC recovery consensus group.pdf>.
5. Llewellyn-Beardsley J, Rennick-Egglestone S, Callard F, et al. Characteristics of mental health recovery narratives: Systematic review and narrative synthesis. *PloS one.* 2019;14(3):e0214678.
6. Alcoholism NIoAAa. Helping patients who drink too much: a clinician's guide, updated 2005 edition. Rockville: National Institutes of Health. 2005.

For peer review only

For peer review only - http://bmjopen.bmj.com/site/about/guidelines.xhtml

Reporting checklist for protocol of a clinical trial.

Based on the SPIRIT guidelines.

Instructions to authors

Complete this checklist by entering the page numbers from your manuscript where readers will find each of the items listed below.

Your article may not currently address all the items on the checklist. Please modify your text to include the missing information. If you are certain that an item does not apply, please write "n/a" and provide a short explanation.

Upload your completed checklist as an extra file when you submit to a journal.

In your methods section, say that you used the SPIRITreporting guidelines, and cite them as:

Chan A-W, Tetzlaff JM, Gøtzsche PC, Altman DG, Mann H, Berlin J, Dickersin K, Hróbjartsson A, Schulz KF, Parulekar WR, Krleža-Jerić K, Laupacis A, Moher D. SPIRIT 2013 Explanation and Elaboration: Guidance for protocols of clinical trials. BMJ. 2013;346:e7586

		Reporting Item	Page Number
**Administrative information**			
Title	#1	Descriptive title identifying the study design, population, interventions, and, if applicable, trial acronym	1
Trial registration	#2a	Trial identifier and registry name. If not yet registered, name of intended registry	1
Trial registration: data set	#2b	All items from the World Health Organization Trial Registration Data Set	1
Protocol version	#3	Date and version identifier	1
Funding	#4	Sources and types of financial, material, and other support	1
Roles and responsibilities: contributorship	#5a	Names, affiliations, and roles of protocol contributors	1, 16

For peer review only

Roles and responsibilities: sponsor contact information	#5b	Name and contact information for the trial sponsor	1
Roles and responsibilities: sponsor and funder	#5c	Role of study sponsor and funders, if any, in study design; collection, management, analysis, and interpretation of data; writing of the report; and the decision to submit the report for publication, including whether they will have ultimate authority over any of these activities	11.12
Roles and responsibilities: committees	#5d	Composition, roles, and responsibilities of the coordinating centre, steering committee, endpoint adjudication committee, data management team, and other individuals or groups overseeing the trial, if applicable (see Item 21a for data monitoring committee)	11,12
**Introduction**			
Background and rationale	#6a	Description of research question and justification for undertaking the trial, including summary of relevant studies (published and unpublished) examining benefits and harms for each intervention	5
Background and rationale: choice of comparators	#6b	Explanation for choice of comparators	5,6
Objectives	#7	Specific objectives or hypotheses	6
Trial design	#8	Description of trial design including type of trial (eg, parallel group, crossover, factorial, single group), allocation ratio, and framework (eg, superiority, equivalence, non-inferiority, exploratory)	6
**Methods: Participants, interventions, and outcomes**			
Study setting	#9	Description of study settings (eg, community clinic, academic hospital) and list of countries where data will be collected. Reference to where list of study sites can be obtained	6
Eligibility criteria	#10	Inclusion and exclusion criteria for participants. If applicable, eligibility criteria for study centres and individuals who will	Table 1

For peer review only

		perform the interventions (eg, surgeons, psychotherapists)	
Interventions: description	#11a	Interventions for each group with sufficient detail to allow replication, including how and when they will be administered	6,7,8
Interventions: modifications	#11b	Criteria for discontinuing or modifying allocated interventions for a given trial participant (eg, drug dose change in response to harms, participant request, or improving / worsening disease)	N/A
Interventions: adherance	#11c	Strategies to improve adherence to intervention protocols, and any procedures for monitoring adherence (eg, drug tablet return; laboratory tests)	11,12
Interventions: concomitant care	#11d	Relevant concomitant care and interventions that are permitted or prohibited during the trial	N/A
Outcomes	#12	Primary, secondary, and other outcomes, including the specific measurement variable (eg, systolic blood pressure), analysis metric (eg, change from baseline, final value, time to event), method of aggregation (eg, median, proportion), and time point for each outcome. Explanation of the clinical relevance of chosen efficacy and harm outcomes is strongly recommended	10, 11
Participant timeline	#13	Time schedule of enrolment, interventions (including any run-ins and washouts), assessments, and visits for participants. A schematic diagram is highly recommended (see Figure)	9
Sample size	#14	Estimated number of participants needed to achieve study objectives and how it was determined, including clinical and statistical assumptions supporting any sample size calculations	9
Recruitment	#15	Strategies for achieving adequate participant enrolment to reach target sample size	6,7,8,9
**Methods: Assignment of interventions (for controlled trials)**			
Allocation: sequence generation	#16a	Method of generating the allocation sequence (eg, computer-generated random numbers), and list of any factors for stratification. To reduce predictability of a random sequence, details of any planned restriction (eg, blocking) should be provided in a separate document that is unavailable to those who enrol participants or assign interventions	9

For peer review only

Allocation concealment mechanism	#16b	Mechanism of implementing the allocation sequence (eg, central telephone; sequentially numbered, opaque, sealed envelopes), describing any steps to conceal the sequence until interventions are assigned	9
Allocation: implementation	#16c	Who will generate the allocation sequence, who will enrol participants, and who will assign participants to interventions	9
Blinding (masking)	#17a	Who will be blinded after assignment to interventions (eg, trial participants, care providers, outcome assessors, data analysts), and how	N/A
Blinding (masking): emergency unblinding	#17b	If blinded, circumstances under which unblinding is permissible, and procedure for revealing a participant's allocated intervention during the trial	N/A
**Methods: Data collection, management, and analysis**			
Data collection plan	#18a	Plans for assessment and collection of outcome, baseline, and other trial data, including any related processes to promote data quality (eg, duplicate measurements, training of assessors) and a description of study instruments (eg, questionnaires, laboratory tests) along with their reliability and validity, if known. Reference to where data collection forms can be found, if not in the protocol	9
Data collection plan: retention	#18b	Plans to promote participant retention and complete follow-up, including list of any outcome data to be collected for participants who discontinue or deviate from intervention protocols	9,10,11
Data management	#19	Plans for data entry, coding, security, and storage, including any related processes to promote data quality (eg, double data entry; range checks for data values). Reference to where details of data management procedures can be found, if not in the protocol	11,12,13
Statistics: outcomes	#20a	Statistical methods for analysing primary and secondary outcomes. Reference to where other details of the statistical analysis plan can be found, if not in the protocol	11
Statistics: additional analyses	#20b	Methods for any additional analyses (eg, subgroup and adjusted analyses)	11

For peer review only

Statistics: analysis population and missing data	#20c	Definition of analysis population relating to protocol non-adherence (eg, as randomised analysis), and any statistical methods to handle missing data (eg, multiple imputation)	11
**Methods: Monitoring**			
Data monitoring: formal committee	#21a	Composition of data monitoring committee (DMC); summary of its role and reporting structure; statement of whether it is independent from the sponsor and competing interests; and reference to where further details about its charter can be found, if not in the protocol. Alternatively, an explanation of why a DMC is not needed	12,13
Data monitoring: interim analysis	#21b	Description of any interim analyses and stopping guidelines, including who will have access to these interim results and make the final decision to terminate the trial	12,13
Harms	#22	Plans for collecting, assessing, reporting, and managing solicited and spontaneously reported adverse events and other unintended effects of trial interventions or trial conduct	12
Auditing	#23	Frequency and procedures for auditing trial conduct, if any, and whether the process will be independent from investigators and the sponsor	11,12,13
**Ethics and dissemination**			
Research ethics approval	#24	Plans for seeking research ethics committee / institutional review board (REC / IRB) approval	1
Protocol amendments	#25	Plans for communicating important protocol modifications (eg, changes to eligibility criteria, outcomes, analyses) to relevant parties (eg, investigators, REC / IRBs, trial participants, trial registries, journals, regulators)	11,12,13
Consent or assent	#26a	Who will obtain informed consent or assent from potential trial participants or authorised surrogates, and how (see Item 32)	11,12,13
Consent or assent: ancillary studies	#26b	Additional consent provisions for collection and use of participant data and biological specimens in ancillary studies, if applicable	N/A
Confidentiality	#27	How personal information about potential and enrolled participants will be collected, shared, and maintained in order to protect	11,12,13

For peer review only

		confidentiality before, during, and after the trial	
Declaration of interests	#28	Financial and other competing interests for principal investigators for the overall trial and each study site	16
Data access	#29	Statement of who will have access to the final trial dataset, and disclosure of contractual agreements that limit such access for investigators	16
Ancillary and post trial care	#30	Provisions, if any, for ancillary and post-trial care, and for compensation to those who suffer harm from trial participation	13
Dissemination policy: trial results	#31a	Plans for investigators and sponsor to communicate trial results to participants, healthcare professionals, the public, and other relevant groups (eg, via publication, reporting in results databases, or other data sharing arrangements), including any publication restrictions	13
Dissemination policy: authorship	#31b	Authorship eligibility guidelines and any intended use of professional writers	13
Dissemination policy: reproducible research	#31c	Plans, if any, for granting public access to the full protocol, participant-level dataset, and statistical code	13
**Appendices**			
Informed consent materials	#32	Model consent form and other related documentation given to participants and authorised surrogates	11-20
Biological specimens	#33	Plans for collection, laboratory evaluation, and storage of biological specimens for genetic or molecular analysis in the current trial and for future use in ancillary studies, if applicable	N/A

The SPIRIT Explanation and Elaboration paper is distributed under the terms of the Creative Commons Attribution License CC-BY-NC. This checklist was completed on 28. June 2021 using https://www.goodreports.org/, a tool made by the EQUATOR Network in collaboration with Penelope.ai

For peer review only
